# α-Phenylthioaldehydes for the effective generation of acyl azolium and azolium enolate intermediates†

**DOI:** 10.1039/d3sc06879j

**Published:** 2024-05-14

**Authors:** Paul M. D. A. Ewing, Pankaj Kumar Majhi, Callum Prentice, Claire M. Young, Karlotta van Rees, Polly L. Arnold, Eli Zysman-Colman, Andrew D. Smith

**Affiliations:** a EaStCHEM, School of Chemistry, University of St Andrews St Andrews, Fife KY16 9ST UK ads10@st-andrews.ac.uk eli.zysman-colman@st-andrews.ac.uk; b EaStCHEM, School of Chemistry, University of Edinburgh EH9 3JF UK; c Dept of Chemistry, University of California Berkeley CA 94720 USA pla@berkeley.edu; d Chemical Sciences Division, Lawrence Berkeley National Laboratory Berkeley CA 94720 USA

## Abstract

α-Phenylthioaldehydes are readily prepared using a simple multi-step procedure and herein are introduced as a new precursor for the NHC-catalysed generation of acyl azolium and azolium enolate intermediates that are of widespread synthetic interest and utility. Treatment of α-phenylthioaldehydes with an NHC precatalyst and base produces an efficient redox rearrangement *via* a Breslow intermediate, elimination of thiophenolate, and subsequent rebound addition to the generated acyl azolium to give the corresponding thiol ester. In the presence of an external alcohol, competition between redox rearrangement and redox esterification can be controlled through judicious choice of the *N*-aryl substituent within the NHC precatalyst and the base used in the reaction. With NEt_3_ as base, NHCs bearing electron-withdrawing (*N*-C_6_F_5_ or *N*-C_6_H_2_Cl_3_) substituents favour redox rearrangement, while triazolium precatalysts with electron-rich *N*-aryl substituents (*N*-Ph, *N*-Mes) result in preferential redox esterification. Using DBU, redox esterification is preferred due to transesterification of the initially formed thiol ester product. Additionally, α-phenylthioaldehyde-derived azolium enolates have been used in enantioselective formal [4 + 2]-cycloaddition reactions to access dihydropyridinone heterocycles with high enantioselectivity (up to >95 : 5 dr, 98 : 2 er).

## Abstract 2

Readily prepared α-phenylthioaldehydes are introduced as acyl azolium and azolium enolate precursors for a range of NHC-mediated catalytic processes incorporating redox rearrangements, redox esterifications and enantioselective [4 + 2]-cycloadditions.

## Graphical abstract

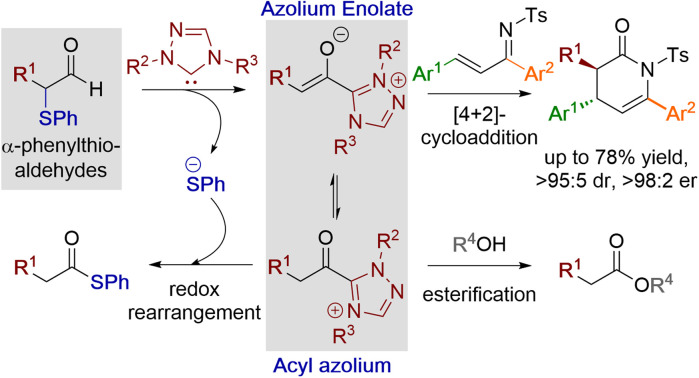

## 1. Introduction

**Fig. 1:**
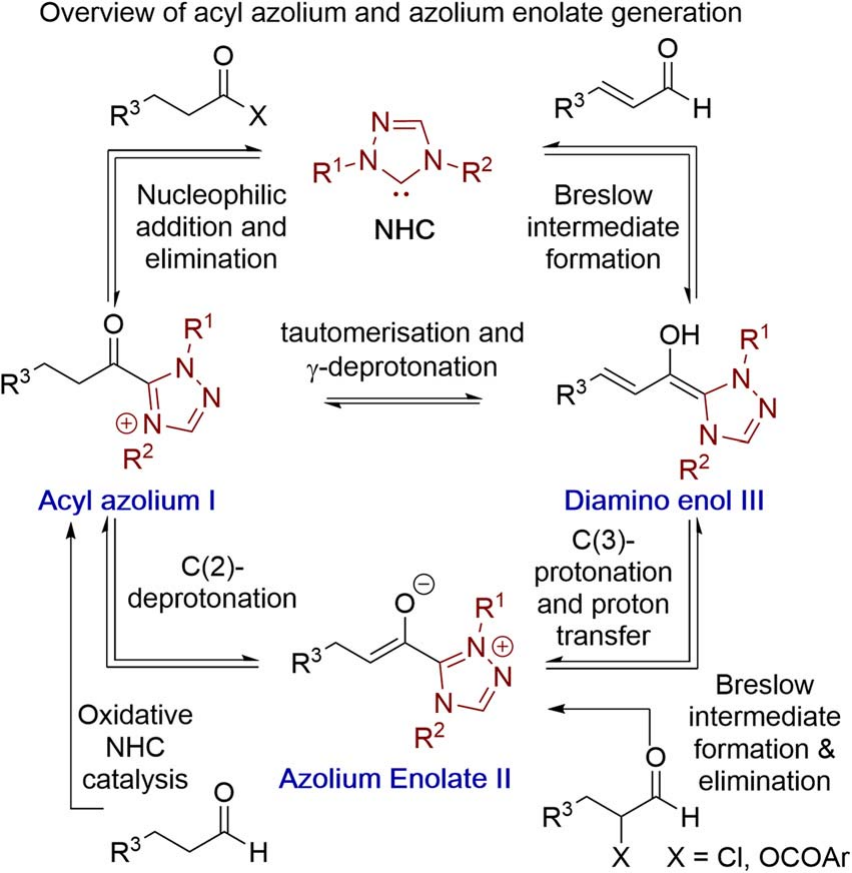
Established access to acyl azolium I and azolium enolate II intermediates.

**Fig. 2:**
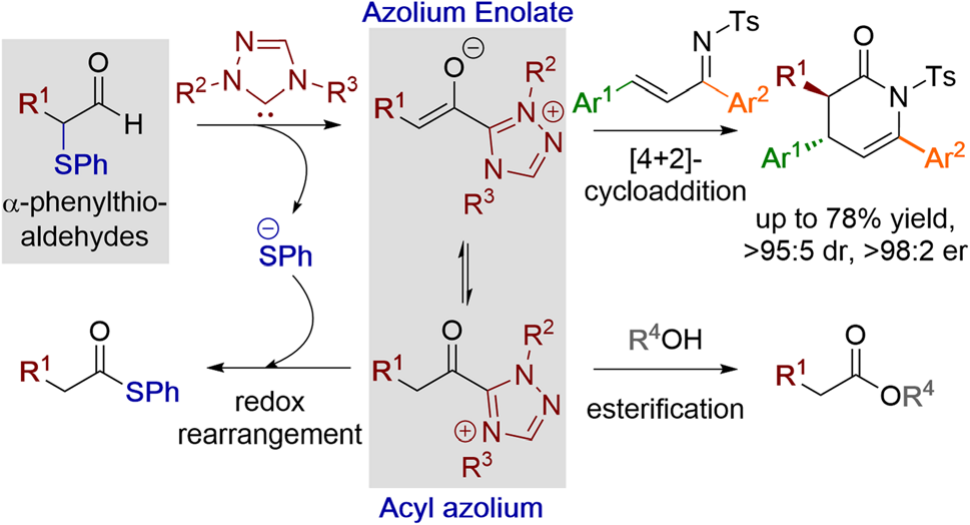
α-Phenylthioaldehydes as acyl azolium and azolium enolate precursors.

Acyl azolium and azolium enolate intermediates have been harnessed as key species within a range of enantioselective reactions using N-heterocyclic carbenes (NHCs).^1^ For example, a variety of effective kinetic resolution and dynamic kinetic resolution processes have been developed that use chiral NHCs as catalysts and exploit *in situ* acyl azolium generation.^1^ Similarly, azolium enolate intermediates have been widely utilised to access important heterocyclic scaffolds *via* formal [2 + 2], [3 + 2], [4 + 2] and higher cycloadditions in an enantioselective manner.^1^ A variety of strategies have been developed to access these reactive species, with an overview of the current methods, as well as their interconnectivity, given in Fig. 1. Acyl azolium intermediates I can be generated directly from the nucleophilic addition–elimination reaction between an NHC and a carboxylic acid derivative such as an ester, anhydride or acid chloride.^2,3^ Alternatively, they can be prepared from the stoichiometric oxidation of an *in situ* generated Breslow intermediate from an aldehyde using so called “oxidative” NHC catalysis, with a range of either transition metal or organic oxidants compatible with this process.^4^ C(2)-deprotonation of an acyl azolium species I leads to the azolium enolate II, while in the reverse direction the selective protonation of an azolium enolate II leads to the generation of an acyl azolium species I.^5^ First demonstrated independently by Scheidt^6^ and Bode,^7^ the generation of acyl azolium species I from enals has also been developed. C(3)-protonation of an enal-derived Breslow intermediate diamino enol III and proton transfer furnishes the azolium enolate II, which undergoes tautomerisation to form the acyl azolium species I.^8,9^ An alternative and direct method to generate the azolium enolate *via* addition of an NHC to a disubstituted ketene was first demonstrated by Ye^10^ and Smith^11^ through formal [2 + 2]-cycloaddition reactions. However, due to limited synthetic diversity of reactive ketene substrates alternative processes to allow the generation of azolium enolates have been developed. For example, Chi and co-workers showed that 4-nitrophenyl esters could be used as azolium enolate precursors,^12^ while the most common process uses α-functionalised aldehydes as precursors.^13^ In this area, elimination of an α-leaving group from an *in situ* generated Breslow intermediate provides the desired enolate. Despite being prone to decomposition, the most common starting materials are α-chloroaldehydes, that have been used to access enantioenriched heterocycles.^14,15^ Furthermore, while α-alkyl-α-chloro-substituted aldehydes are generally tolerated, only limited precedent for the use of α-unsubstituted or α-aryl substituted precursors have been developed.^16^ In previous work, we introduced bench stable α-aroyloxyaldehydes as alternative acyl azolium and azolium enolate precursors.^13*a*^ They have since been applied in [2 + 2], [3 + 2] and [4 + 2] cycloadditions,^17^ redox α-aminations,^18^ as well as kinetic resolutions and desymmetrisations,^19^ but again α-unsubstituted or α-aryl substituted derivatives were not tolerated.^13*a*^ Building upon these prior demonstrations, in this manuscript the synthesis and reactivity of α-phenylthioaldehydes as acyl azolium and azolium enolate intermediate precursors is described (Fig. 2). Notably, in this work thiophenolate eliminated during azolium enolate formation is harnessed as an *in situ* nucleophile to turnover the NHC catalyst from an acyl azolium intermediate, leading to thiol ester products resulting from redox rearrangement. This process is tolerant of both α-unsubstituted and α-aryl substituted derivatives. In the presence of an external alcohol, competition between redox rearrangement and redox esterification can be controlled through judicious choice of the *N*-aryl substituents within the NHC precatalyst and the base used in the reaction. In addition, α-phenylthioaldehyde-derived azolium enolates have been used in enantioselective formal [4 + 2]-cycloaddition reactions to access dihydropyridinone heterocyclic products with high enantioselectivity (up to >95 : 5 dr, 98 : 2 er).

## 2. Results and discussion

### 2.1 Synthesis of α-phenylthioaldehydes

**Scheme 1:**
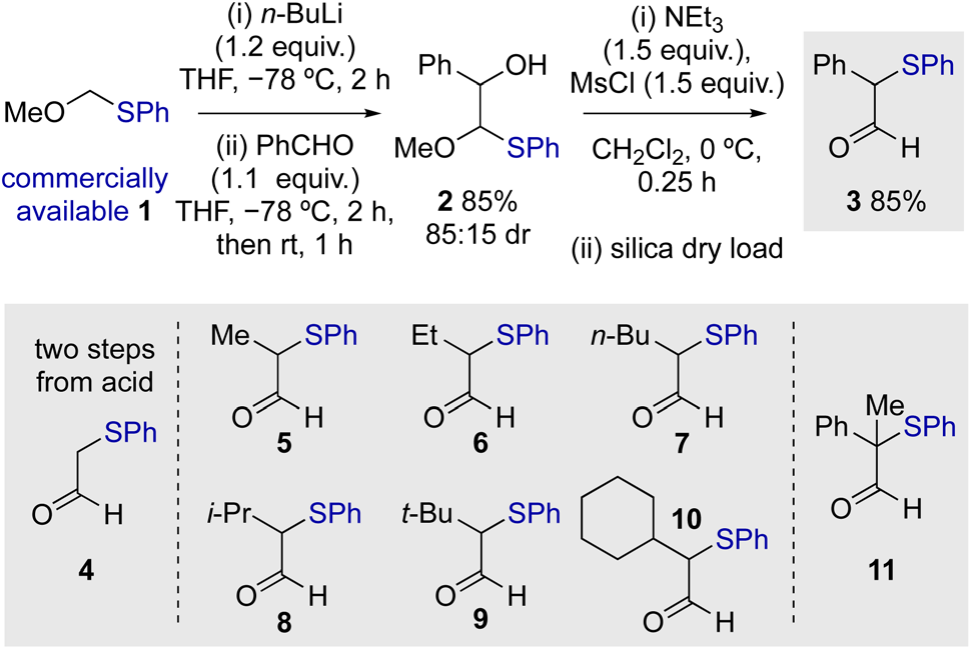
Synthesis of α-phenylthioaldehydes.

Initial studies commenced by demonstrating a viable route to a model starting material. α-Phenylthioaldehyde 3 bearing an α-phenyl substituent was synthesised by modification of a two-step procedure first reported by Nozaki and co-workers (Scheme 1).^20^ Starting from commercially available methoxymethyl(phenylsulfide) 1, deprotonation and subsequent aldol reaction gave 2 in 85% yield (85 : 15 dr), with mesylation promoting rearrangement to give 3 in 85% yield. Modification of the reported procedure for the second step, including the use of dichloromethane as solvent and dry loading on silica, resulted in reproducible product yields and simple purification (see ESI† for details). Using this general approach, a range of differently substituted α-phenylthioaldehydes 5–11 were prepared, incorporating α-alkyl as well as an α,α-disubstituted aldehyde. The unsubstituted aldehyde 4 was prepared by an alternative two-step procedure from the corresponding acid involving methyl ester formation and selective reduction with DIBAL (see ESI† for further details).

### 2.2 NHC-catalysed redox rearrangement

Studies subsequently investigated the proposed NHC-catalysed internal redox rearrangement of α-phenyl-α-phenylthioaldehyde 3 to give the corresponding thiol ester 12 as a model substrate for reaction development. Aldehyde 3 was treated with a variety of triazolium NHC precatalysts with different *N*-aryl substituents (13–16) and bases.^21^ In the presence of *N*-mesityl triazolium precatalyst 13 (10 mol%) and Cs_2_CO_3_ as a base, thiol ester 12 was formed in 97% yield in 16 hours (Table 1, entry 1). Lowering the catalyst loading to 5 mol% resulted in full conversion of the aldehyde but required an extended reaction time of 24 hours (Table 1, entry 2). Changing the base from Cs_2_CO_3_ to the organic bases NEt_3_ or DBU gave comparable reaction yields (Table 1, entry 3 and 4). The effect of alteration of the *N*-aryl substituent within the precatalyst was next explored using NEt_3_ as the base. *N*-Phenyl substituted NHC precatalyst 14 (10 mol%) gave 95% conversion to the thiol ester 12, while both *N*-pentafluorophenyl precatalyst 15 and *N*-2,4,6-trichlorophenyl precatalyst 16 (10 mol%) gave high product yield with a significantly shorter reaction time of only 15 minutes (Table 1, entries 5 and 6). Using NHC precatalyst 16 at 5 mol% loading led to good product conversion within 2 hours (Table 1, entry 8). Control reactions in the absence of NHC precatalyst gave no rearrangement product or degradation of the aldehyde (Table 1, entry 9).

**Table 1:** Optimisation of NHC-catalysed redox rearrangement^a^

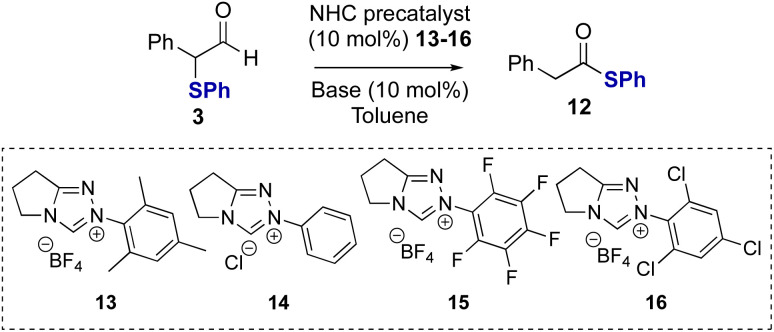			
Entry	Conditions	Yield^b^^,^^c^ (%)	Time^d^ (h)
1	13, Cs_2_CO_3_	>95 (97)	16
2^e^	13, Cs_2_CO_3_	>95	24
3	13, NEt_3_	>95 (97)	16
4	13, DBU	>95	16
5	14, NEt_3_	>95	16
6	15, NEt_3_	>95 (97)	0.25
7	16, NEt_3_	>95 (98)	0.25
8^e^	16, NEt_3_	>95 (95)	2
9	NEt_3_	0	24

a All reactions conducted in a flame-dried Schlenk flask under a nitrogen atmosphere using aldehyde 3 (0.166 mmol), base (10 mol%), NHC precatalyst 13-16 (10 mol%).

b Conversion was determined by ^1^H NMR spectroscopy of the crude reaction mixtures.

c Yields in parentheses correspond to isolated yields after chromatographic purification.

d Reaction times refer to aliquots taken from the reaction mixture at various time points followed by ^1^H NMR spectroscopic analysis.

e 5 mol% catalyst was used.

**Scheme 2:**
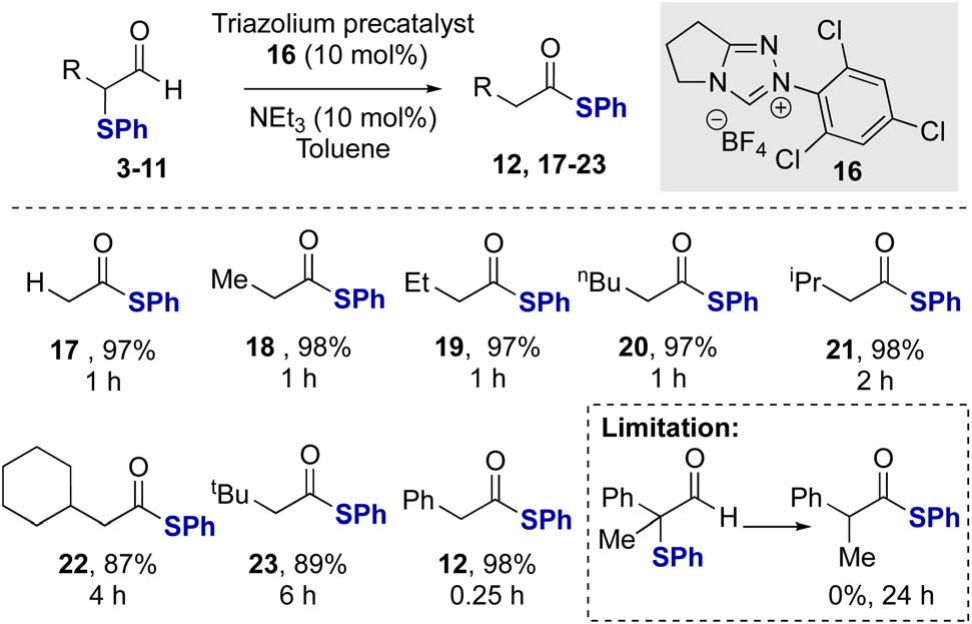
Redox rearrangement of α-phenylthioaldehydes.^a^ All reactions were conducted in a flame-dried Schlenk flask under a nitrogen atmosphere using 0.166 mmol of aldehyde 3–11, 10 mol% NEt_3_.

After demonstrating the feasibility of this redox rearrangement process, its scope and limitations were established using *N*-2,4,6-trichlorophenyl precatalyst 16 and NEt_3_ as base. The unsubstituted aldehyde 4 gave the corresponding thiol ester 17 in 97% isolated yield (Scheme 2). α-Phenylthioaldehydes bearing α-alkyl substituents were tolerated in this redox rearrangement although the rate of rearrangement, and thus required reaction time, is significantly affected by the steric bulk of the α-substituent. For example, using linear Me, Et and *n*-Bu α-substituents, the rearrangement was complete within 1 hour. The incorporation of branched α-substituents required longer reaction times for effective rearrangement, with α-i-Pr aldehyde 8 requiring two hours to give 21 at full conversion. Similarly, the α-cyclohexyl and α-*t*-Bu aldehydes 9 and 10 required 4 and 6 hours, respectively, for full conversion to the corresponding products 22 and 23.^22^ These results contrast the efficient rearrangement of the α-aryl substituted derivative 3, where full conversion to the product 12 was observed within just 15 minutes. This is hypothesized to reflect the ability of the conjugating α-phenyl substituent to stabilize the azolium enol/enolate intermediate within this process.^23^ A limitation of this methodology showed that α-phenylthioaldehyde 11 bearing a tertiary centre was unsuccessful in this catalytic rearrangement, presumably due to steric hindrance inhibiting NHC addition to the aldehyde.

### 2.3 NHC-catalysed redox esterification

Further investigation explored α-phenylthioaldehydes in NHC-catalysed redox esterification reactions using benzyl alcohol. Using α-phenyl-α-phenylthioaldehyde 3 as a model, at the onset the potential for mixtures of benzyl ester 24 and thiol ester 12 products, arising either directly from competition between nucleophiles, or from transesterification, was considered (Table 2). The use of NEt_3_ as base was initially probed, with *N*-mesityl substituted triazolium precatalyst 13 leading exclusively to benzyl ester product 24 (entry 1). Using *N*-phenyl precatalyst 14 gave a 75 : 25 mixture of benzyl ester 24 : thiol ester 12 products, with benzyl ester 24 isolated in 70% yield (entry 2). The use of *N*-pentafluorophenyl precatalyst 15 gave a 50 : 50 ratio of benzyl ester 24 to thiol ester 12 (entry 3), while *N*-2,4,6-trichlorophenyl precatalyst 16 led to a 10 : 90 ratio of benzyl ester 24 to thiol ester 12 (entry 4). Further studies considered the effect of the base on product selectivity. Using DBU, *N*-mesityl precatalyst 13 again gave the benzyl ester 24 as the major product (entry 5). However, using *N*-2,4,6-trichlorophenyl triazolium precatalyst 16 and DBU, the benzyl ester product 24 was generated exclusively, indicating a switch in product selectivity with base for precatalyst 16 (entry 6). Similarly, the use of benzylamine in this redox transformation using precatalyst 13 and DBU led to productive amide bond formation in 91% yield (see ESI† for further details).

**Table 2:** A Redox esterification optimisation^a^

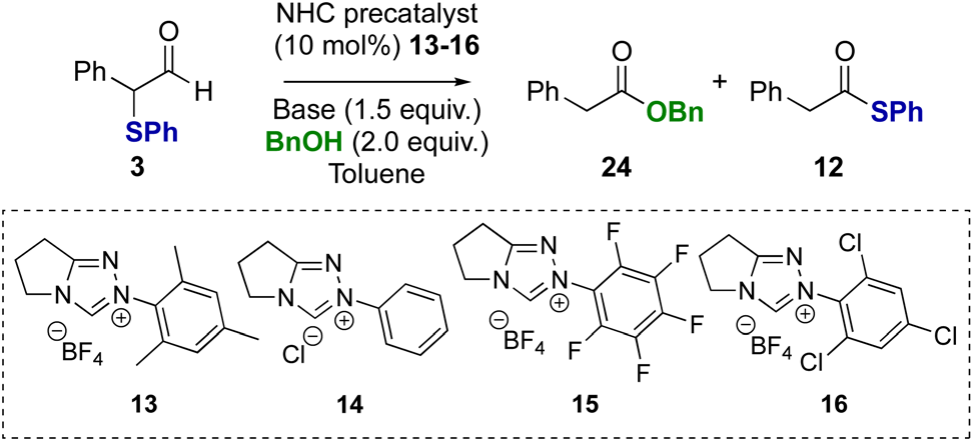				
Entry^a^	Precat, base	Conv.^b^	24^b^^,^^c^ (%)	12^b^^,^^c^ (%)
1	13, NEt_3_	>95	>95 (97)	<5
2	14, NEt_3_	>95	76 (70)	23 (21)
3	15, NEt_3_	>95	50 (46)	50 (47)
4	16, NEt_3_	>95	10 (5)	90 (87)
5	13, DBU	>95	>95 (95)	<5
6	16, DBU	>95	>95 (98)	<5

a All reactions conducted using aldehyde 3 (0.166 mmol), base (1.5 equiv.), NHC precatalyst 13–16 (10 mol%), BnOH (2 equiv.).

b Conversion, as well as ratio of ester and thiol ester products, determined by ^1^H NMR spectroscopic analysis of the crude reaction product.

c Parentheses indicate isolated yields after chromatographic purification.

**Scheme 3:**
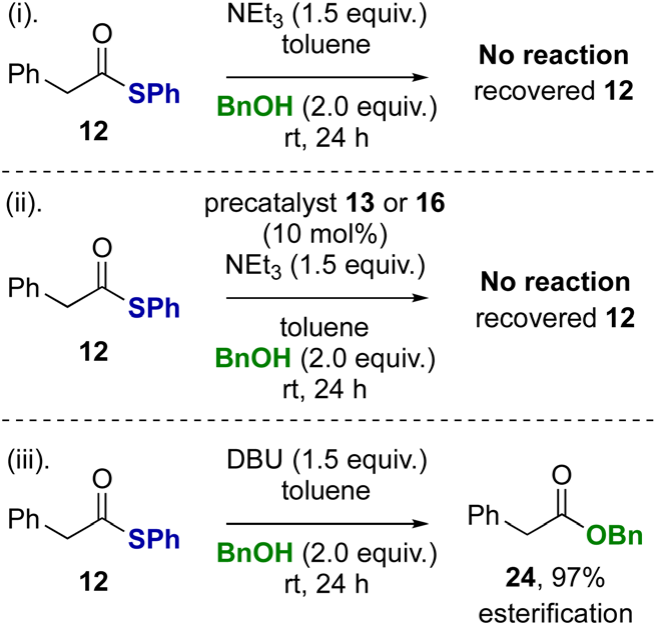
Control studies: esterification of thiol ester.

Control reactions were subsequently performed to rationalise the product distributions observed (Scheme 3). Potential transesterification to account for the interconversion between thiol ester 12 and benzyl ester 24 during the reaction was tested (reactions i–iii). Treatment of thiol ester 12 with BnOH (2 equiv.) and NEt_3_ (reaction i) or NEt_3_ in the presence NHC precatalysts 13 or 16 (reaction ii) returned exclusively starting material, with no transesterification observed. However, treatment of thiol ester 12 with DBU and benzyl alcohol (2 equiv.) showed complete transesterification to benzyl ester 24 within 24 hours (reaction iii).

To probe if these results were unique to α-phenyl-α-phenylthioaldehyde, α-methyl substituted substrate 5 was tested in the redox esterification reaction (Table 3). Using NHC precatalysts 13 or 16 and either NEt_3_ or DBU, similar trends in product distributions were observed; using NEt_3_ a reduced rate of product formation was observed, with *N*-mesityl precatalyst 13 favouring benzyl ester 25 (Table 3, entry 1) and *N*-2,4,6-trichlorophenyl precatalyst 16 favouring thiol ester 26 (Table 3, entry 2). The use of DBU promoted full conversion selectively to the benzyl ester 25 irrespective of NHC precatalyst (Table 3, entries 3 and 4). Changing from benzyl alcohol to methanol with DBU as the base led to selective formation of the methyl ester 27 (entries 5 and 6).

**Table 3:** Redox esterification: probing generality with alcohol and substrate^a^

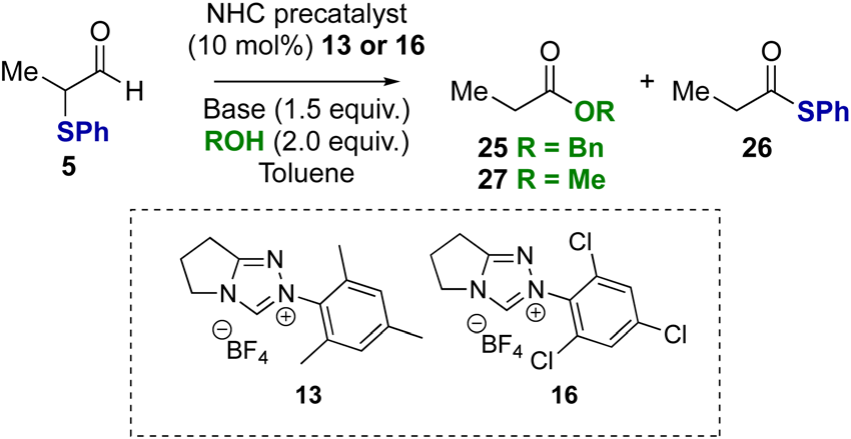					
Entry^a^	R	Precat, base	Conv.^b^	Ester^b^^,^^c^ (%)	26^b^^,^^c^ (%)
1	Bn	13, NEt_3_	40	80	20
2	Bn	16, NEt_3_	>95	43 (40)	57 (55)
3	Bn	13, DBU	>95	>95 (97)	<5
4	Bn	16, DBU	>95	>95 (98)	<5
5	Me	13, DBU	45	>95	<5
6	Me	16, DBU	>95	>95 (95)	<5

a All reactions conducted using aldehyde 5 (0.166 mmol), base (1.5 equiv.), NHC precatalyst 13 or 16 (10 mol%), ROH (2 equiv.).

b Conversion, as well as ratio of ester and thiol ester products, determined by ^1^H NMR spectroscopic analysis of the crude reaction product.

c Parentheses indicate isolated yields after chromatographic purification.

### 2.5 Interpretation of experimental results and mechanistic construct

These experimental results are consistent with the electronic nature of the *N*-aryl substituent within the NHC and the base as the main factors influencing selectivity in these processes. Firstly, with NEt_3_, NHCs derived from triazolium precatalysts 15 and 16 bearing electron-withdrawing *N*-aryl substituents favour redox rearrangement, consistent with the observation of enhanced conversion rate to the thiol ester in comparison to triazolium precatalysts 15 and 16. Switching to triazolium precatalysts with more electron-rich *N*-aryl substituents (triazoliums 13 and 14), results in preferential redox esterification in the presence of BnOH. The choice of base also influences product selectivity as the presence of DBU overrides the intrinsic selectivity of the triazolium precatalyst due to transesterification of any thiol ester product. These observations can be explained by considering mechanistic observations reported by Bode^24^ and Berkessel.^5*a*^ Bode showed that triazolylidenes bearing electron-donating *N*-aryl substituents (such as *N*-mesityl) favour azolium enolate formation, while *N*-aryl groups bearing electron-withdrawing substituents (C_6_F_5_ or C_6_H_2_Cl_3_) favour acyl azolium intermediates.^25^ A detailed mechanistic study by Berkessel and co-workers has recently shown that NHC-catalysed redox esterification of aldehydes with imidazolium based NHC catalysts proceeds through an azolium enolate intermediate.^5*a*^

**Fig. 3:**
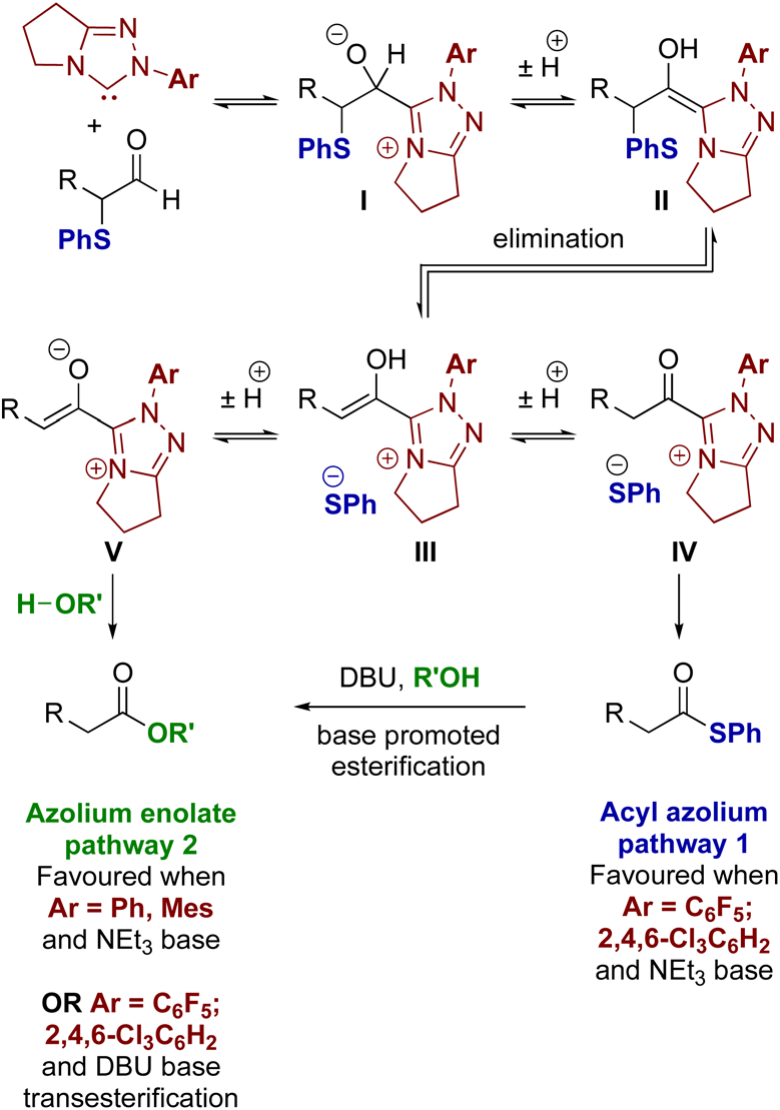
Proposed mechanism for acyl azolium and azolium enolate generation.

With these principles in mind, a plausible mechanism for the processes reported here is outlined in Fig. 3. Initially, the triazolium precatalyst is deprotonated by base to generate the corresponding NHC. Addition to the α-phenylthioaldehyde generates the corresponding tetrahedral adduct I, which after proton transfer gives the corresponding Breslow intermediate II. Elimination of thiophenolate gives the azolium enol intermediate ion pair III. For triazolium precatalysts with electron-withdrawing *N*-aryl substituents (15 and 16) tautomerisation to the acyl azolium IV is preferred, and reaction of IV with the thiophenolate counterion generates the thiol ester. For triazolium precatalysts bearing electron donating *N*-aryl substituents such as 13 and 14 (when NEt_3_ is used as base), the azolium enolate intermediate V is preferred. Reaction of V with alcohols (R′OH) *via* a concerted deprotonation-nucleophilic attack as outlined by Berkessel forms the ester product. In the presence of DBU, the thiol ester can be converted to the corresponding ester product.

### 2.6 NHC-catalysed formal [4 + 2] cycloadditions

**Scheme 4:**
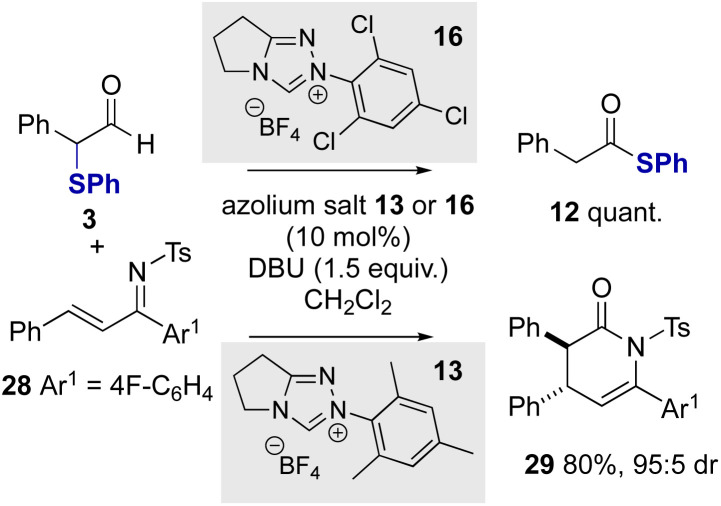
Evaluation of azolium enolate generation with NHC catalysts.

Further work sought to employ α-phenylthioaldehydes in an enantioselective C–C bond forming reaction between azolium enolates and α,β-unsaturated tosyl imines.^12*a*^ Given the trends in reactivity observed with variation of the *N*-aryl substituent of the NHC, initial studies probed the effect of the catalyst *N*-aryl substituent on product distribution in the racemic series using α-phenylthioaldehyde 3 and α,β-unsaturated tosyl imine 28 as substrates (Scheme 4). Consistent with the previous observations, *N*-2,4,6-trichlorophenyl triazolium precatalyst 16 gave exclusively thiophenyl ester 12 in quantitative yield with no dihydropyridone product observed. However, when using *N*-mesityl precatalyst 13 in the presence of DBU as base the product from preferential azolium enolate formation was observed, giving the desired (±)-cycloadduct 29 in 80% yield as a single diastereoisomer (>95 : 5 dr).

**Scheme 5:**
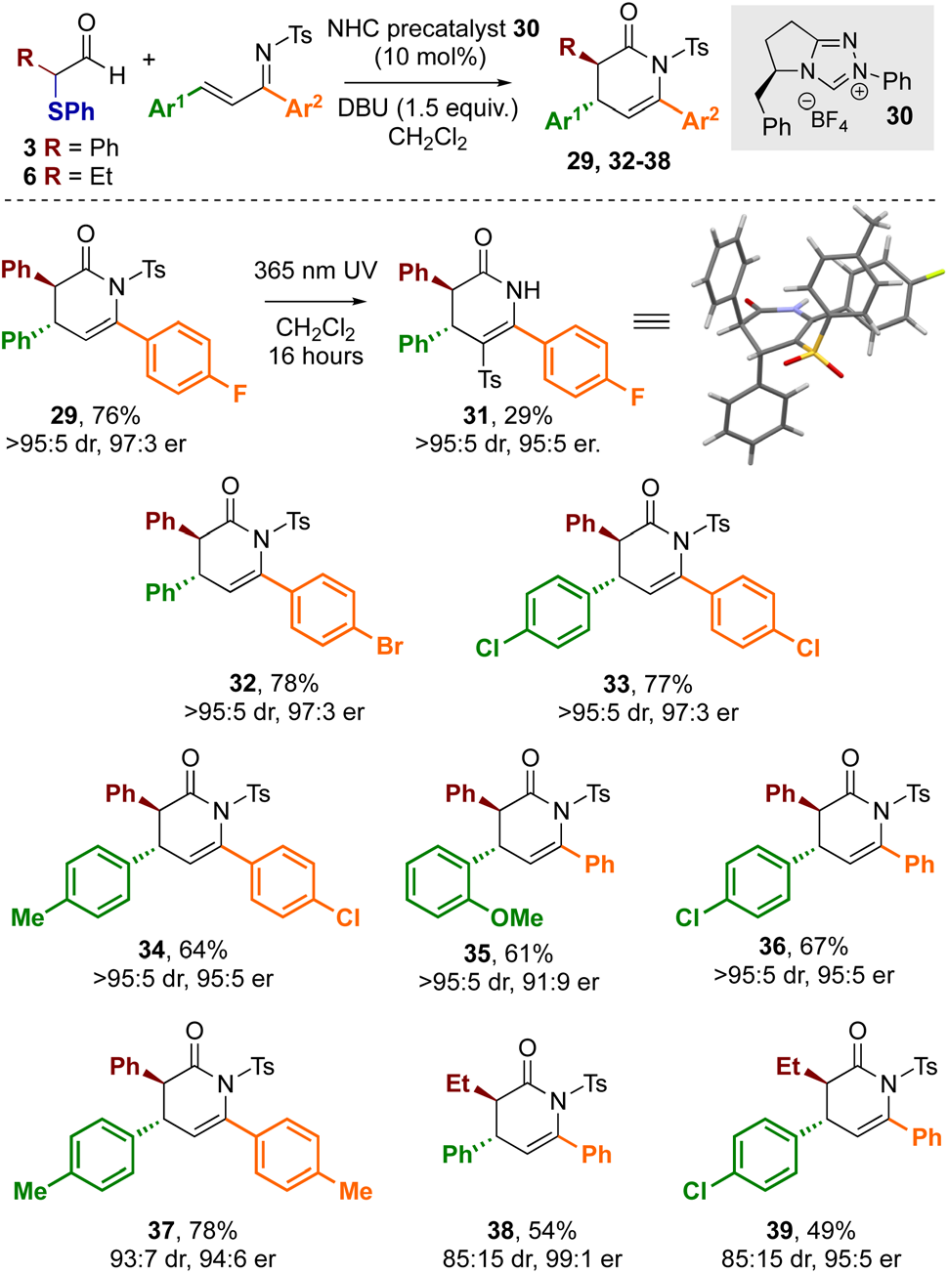
Scope of formal [4 + 2] cycloadditions.^a^ All reactions were conducted in a flame dried Schlenk flask under a nitrogen atmosphere using 0.4 mmol of 3, 0.2 mmol of the corresponding imine.^b^ Yields are isolated yields ^c^ dr was determined by ^1^H NMR spectroscopy of the crude reaction mixtures, er of purified product was determined by HPLC analysis on a chiral stationary phase. X-ray representation; blue = N; red = O, dark grey = C, light grey = H, yellow = S, green = F.

Building on these observations, the use of enantiopure NHC precatalyst 30 bearing an electron rich *N*-Ph substituent was investgated for enantioselective cycloaddition reactions. Using 30 with DBU as the base and α-phenylthioaldehyde 3 with α,β-unsaturated tosyl imine 28 gave the desired dihydropyridinone product 29 in 76% yield in excellent stereoselectivity (>95 : 5 dr, 97 : 3 er, Scheme 5). The relative and absolute configuration of 29 was unambiguously determined by X-ray crystallographic analysis of derivative 31,^26^ derived from facile N- to C-sulfonyl transfer of the product,^27^ and is consistent with the selectivity observed in previous NHC-catalysed [4 + 2] cycloadditions (Scheme 4).^12*a*^ Using aldehyde 3 a variety of substrates were explored in this protocol through variation of the aromatic substituents within the α,β-unsaturated tosyl imine reactant. In all cases, the corresponding dihydropyridinones 32–37 were isolated in good yields (61–78%) and in excellent stereoselectivity (typically >95 : 5 dr and ∼95 : 5 er), demonstrating the use of α-phenylthioaldehyde 3 as an effective azolium enolate precursor. Furthermore, the demonstration of this process on a preparative laboratory scale was investigated, allowing the preparation of 36 using the corresponding imine (1.5 mmol scale rather than 0.2 mmol), giving 36 in 57% yield (438 mg, 95 : 5 dr, 95 : 5 er). To further develop the scope of this process, extension to aliphatic aldehyde 6 as the azolium enolate precursor was applied, giving dihydropyridinones 38 and 39 in 54 and 49% yield respectively and in excellent stereoselectivity (85 : 15 dr, 99 : 1 er and 85 : 15, 95 : 5 er respectively).

## 3. Conclusions

In summary, α-phenylthioaldehydes were prepared by a simple two-step synthesis from commercially available reagents. They can act as acyl azolium and azolium enolate precursors for a range of NHC-mediated catalytic processes. Due to the efficient leaving group ability and nucleophilicity of thiophenolate,^28^ a variety of thiol esters can be prepared from these species *via* redox rearrangement. In the presence of an external alcohol, competition between redox rearrangement and redox esterification can be controlled through judicious choice of the *N*-aryl substituent within the NHC precatalyst and the base used in the reaction. With NEt_3_ as base, NHCs bearing electron withdrawing (*N*-C_6_F_5_ or *N*-C_6_H_2_Cl_3_) substituents favour redox rearrangement, while triazolium precatalysts with electron-rich *N*-aryl substituents (*N*-Mes) result in preferential redox esterification. Using DBU, redox esterification is preferred due to transesterification. α-Phenylthioaldehyde-derived azolium enolates are also used for enantio- and diastereoselective [4 + 2]-cycloaddition reactions with α,β-unsaturated tosyl imines, giving dihydropyridinone derivatives with excellent stereocontrol (up to >95 : 5 dr, >98 : 2 er).

## Data availability

All data (experimental procedures and characterization) that support the findings of this study are available within the article and its ESI.† Crystallographic data for compound 31 has been deposited with the Cambridge Crystallographic Data Centre under deposition numbers 2310270. The research data supporting this publication can be accessed from “α-phenylthioaldehydes for the effective generation of acyl azolium and azolium enolate intermediates”. University of St Andrews Research Portal. DOI: https://doi.org/10.17630/b0dfa038-03b4-4054-a2a3-72a4e1593b16. PURE ID: 302087534.

## Author contributions

ADS conceived the project; PE and PKM carried out experimental studies in consultation with CMY. CP carried out preliminary studies. ADS and PKM wrote the manuscript. KvR carried out single crystal X-ray analysis. ADS and PLA raised the funding. All authors reviewed, edited, and agreed on the finalised version of the manuscript.

## Conflicts of interest

There are no conflicts to declare.

## Supplementary Material

SC-015-D3SC06879J-s001

SC-015-D3SC06879J-s002
