# Supplementary material for: α-Phenylthioaldehydes for the effective generation of acyl azolium and azolium enolate intermediates
Source: Chem Sci. 2024 May 14;15(24):9369–75. doi: 10.1039/d3sc06879j (PMC11186317; doi:10.1039/d3sc06879j)
Supplement: SC-015-D3SC06879J-s001 [file SC-015-D3SC06879J-s001.pdf]

Electronic Supplementary Material (ESI) for Chemical Science.
This journal is © The Royal Society of Chemistry 2024

α-Phenylthioaldehydes for the effective generation of acyl azolium and azolium enolate intermediates

Paul M. D. A. Ewing,[a,b] Pankaj Kumar Majhi,[a] Callum Prentice,[a] Claire M. Young,[a] Karlotta van Rees,[b] Polly L. Arnold,[c,d]* Eli Zysman-Colman[a]* and Andrew D. Smith[a]*

Supporting Information

Table of Contents

1. General Information……………………………………………………….S2
2. General Procedures……………………………………………………….. S4
3. Characterisation of compounds……………………………………………S6
4. Crystallography Details……………………………………………………S24
5. References…………………………………………………………………S25
6. NMR Spectra………………………………………………………………S27
7. HPLC Spectra………………………………………………………………S50

1. General Information

1.1 Materials

Reactions involving moisture sensitive reagents were carried out in flame-dried glassware under a nitrogen atmosphere using standard vacuum line techniques and using anhydrous solvents. Anhydrous toluene, CH_2Cl_2, THF and Et_2O were obtained from an anhydrous solvent system (purified using an alumina column, Mbraun SPS-800). All other solvents and commercial reagents were used as supplied without further purification.

1.2 Methods

Room temperature (rt) refers to 20-25 ˚C. Temperatures of 0 ˚C and −78 ˚C were obtained using ice/water and CO_2(s)/acetone baths, respectively. Reactions that required heating were performed using a DrySyn on a stirrer hotplate equipped with a contact thermocouple. *in vacuo* refers to the use either a Büchi Rotavapor R-200 with a Büchi V-491 heating bath and Büchi V-800 vacuum controller; a Büchi Rotavapor R-210 with a Büchi V-491 heating bath and Büchi V850 vacuum controller; a Heidolph Laborota 4001 with vacuum controller; an IKA RV10 rotary evaporator with an IKA HB10 heating bath and ILMVAC vacuum controller; or an IKA RV10 rotary evaporator with an IKA HB10 heating bath and Vacuubrand CVC3000 vacuum controller. Rotary evaporator condensers are fitted to Julabo FL601 Recirculating Coolers filled with ethylene glycol and set to –5 ˚C.

Analytical thin layer chromatography was performed on pre-coated aluminium plates (Kieselgel 60 F254 silica). TLC visualisation was carried out with ultraviolet light (254 nm), followed by staining with a 1% aqueous $KMnO_4$ solution. Flash column chromatography was performed on Kieselgel 60 silica (0.040-0.063 mm pore size; 230-400 mesh ASTM) in the solvent system stated.

Melting points were recorded on an Electrothermal 9100 melting point apparatus and are uncorrected.

HPLC analyses were obtained using either a Shimadzu HPLC consisting of a DGU-20A5 degassing unit, LC-20AT liquid chromatography pump, SIL-20AHT autosampler, CMB-20A communications bus module, SPD-M20A diode array detector and a CTO-20A column oven; or a Shimadzu HPLC consisting of a DGU-20A5R degassing unit, LC-20AD liquid chromatography pump, SIL-20AHT autosampler, SPD-20A UV/Vis detector and a CTO-20A column oven. Separation was achieved using DAICEL CHIRALCEL OD-H column or

DAICEL CHIRALPAK AD-H column using the S3 method stated. HPLC traces of enantiomerically enriched compounds were compared with spectra of authentic racemic samples.

^{1}H and $^{13}C\{^{1}H\}$ nuclear magnetic resonance (NMR) spectra were acquired on either a Bruker Avance II 400 (1H 400 MHz) or a Bruker Avance II 500 (^{1}H 500 MHz; $^{13}C\{^{1}H\}$ 126 MHz) spectrometer at ambient temperature in the deuterated solvent stated. All chemical shifts are quoted in parts per million (ppm) and referenced to the residual solvent peak. All coupling constants, J, are quoted in Hz. Multiplicities are indicated by: s (singlet), d (doublet), t (triplet), q (quartet), sept. (septet), dd (doublet of doublets), dq (doublet of quartets), ddd (doublet of doublet of doublets), and m (multiplet). The abbreviation Ar is used to denote aromatic, Ph to denote phenyl, br to denote broad and app to denote apparent. NMR peak assignments were confirmed using 2D ^{1}H–^{13}C heteronuclear single quantum coherence (HSQC) and 2D ^{1}H–^{13}C heteronuclear multiple-bond correlation spectroscopy (HMBC) where necessary.

Infrared spectra were recorded on a Shimadzu IRAffinity-1 Fourier transform IR spectrophotometer fitted with a Specac Quest ATR accessory (diamond puck). Spectra were recorded of either thin films or solids, with characteristic absorption wavenumbers (ν_{max}) reported in cm^{-1}.

Mass spectrometry (m/z) data were acquired by electrospray ionization (ESI) or nanospray ionization (NSI) at the University of St Andrews.

2. General procedures

2.1 Synthesis of Triazolium Precatalysts

The triazolium precatalysts according to the reported literature procedures.[1]

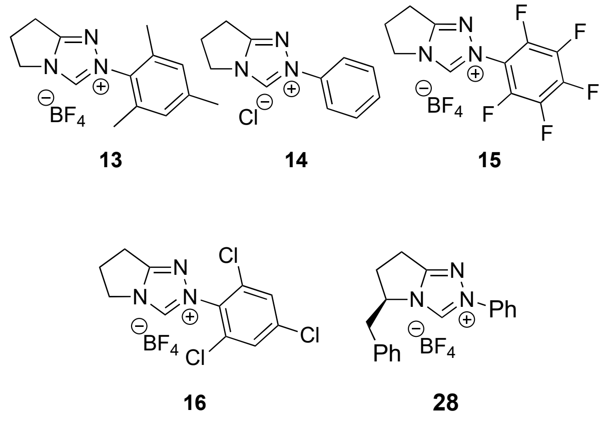

Figure 1: Triazolium precatalysts.

2.2 General procedure: Synthesis of α–Phenylthioalcohols

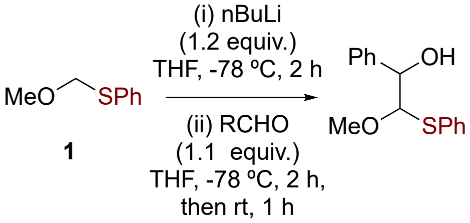

Using a modification of the procedure reported by Nozaki.[2] To a flame dried two-neck round bottom flask under N_2 was added (methoxymethyl)(phenyl) sulfide (0.69 mL, 4.70 mmol, 1 equiv.) in anhydrous THF (10 mL) at –78 ˚C. A solution of n-butyl lithium 2.5 M in hexanes (2.26 mL, 5.64 mmol, 1.2 equiv.) was then added dropwise and the reaction was stirred at –78 ˚C for two hours. The selected aldehyde or ketone (5.17 mmol, 1.1 equiv.) was subsequently added dropwise, and the reaction was stirred at –78 ˚C for one hour. The reaction was allowed to warm to room temperature and was stirred for a further one hour, before being quenched by the addition of deionised water (20 mL) under a flow of N_2. The mixture was extracted with EtOAc (2 × 20 mL). The combined organic phases were dried over $MgSO_4$ and concentrated under reduced pressure. Purification using silica column chromatography gave the title products with spectroscopic data in accordance with literature or fully characterised if novel.

2.3 General procedure: Synthesis of α-phenylthioaldehydes

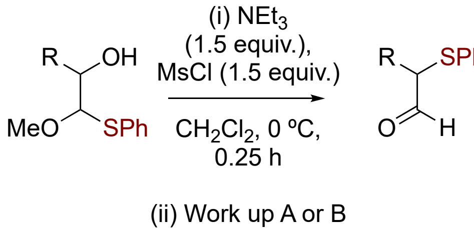

Using a modification of the procedure reported by Nozaki,[2] triethylamine (0.42 mL, 3 mmol, 1.5 equiv.) was added to a solution of α-phenylthioalcohol (2 mmol, 1.0 equiv.) in Solvent (10 mL, 0.2 M) in a flame dried flask under N_2 at 0 ˚C and stirred for five minutes. Methanesulfonyl chloride (0.23 mL, 3 mmol, 1.5 equiv.) was added dropwise under a flow of N_2, and the reaction was stirred for between 1 - 12 hours depending on the substrate (R = aryl 1 hr, R = alkyl 12 hours). Silica (Kieselgel 60, 15 equiv.) was then added to the solution.

A) Original work-up) The mixture was stirred for 15 minutes before the slurry was filtered through a silica plug. The silica plug was washed with toluene and then CH_2Cl_2. The filtrate was concentrated at reduced pressure and the residue was purified by column chromatography eluting with EtOAc:hexane.

B) Modified work-up) The solvent was then carefully removed at reduced pressure until the silica was free flowing and dry. The silica was then dry loaded onto a prepacked column fitted with a frit and eluted with EtOAc:hexane. **Recommended work-up, gives cleaner products and higher isolated yields.**

2.4 General procedure: NHC-catalysed redox rearrangement.

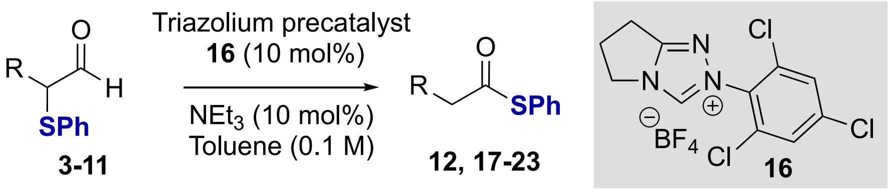

To a flame dried Schlenk tube under N_2 was added α-phenylthioaldehyde (1 equiv.), NHC precatalyst (0.1 equiv.) and anhydrous toluene (0.1 M). The reaction was stirred for five minutes before triethylamine (0.1 equiv.) was added via Hamiltonian syringe. The reaction was stirred for the appropriate reaction time under a nitrogen atmosphere. The solvent was removed under reduced pressure and the crude residue was purified using flash column chromatography eluting with hexane:EtOAc to give the thiolester products.

2.5 General procedure: NHC-catalysed redox esterification

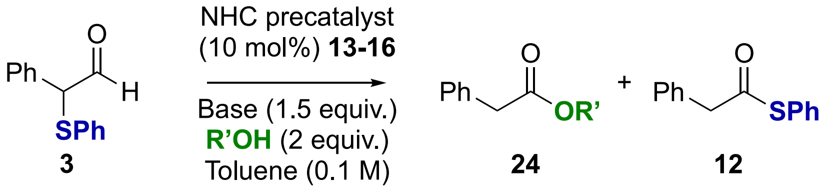

To a flame dried Schlenk tube under N_2 was added α-phenylthioaldehyde (1 equiv.), NHC precatalyst (0.1 equiv.), alcohol (2 equiv.) and anhydrous toluene (0.1 M). The reaction was stirred for five minutes before base (1.5 equiv.) was added via Hamiltonian syringe. The reaction was stirred for 16 h under a nitrogen atmosphere. The reaction was washed with 0.1 M HCl, followed by brine and the organics were dried over $MgSO_4$. The solvent was removed under reduced pressure and the crude residue was purified using column chromatography eluting with hexane:EtOAc to give the ester products.

2.6 General procedure: NHC-catalysed redox amidation

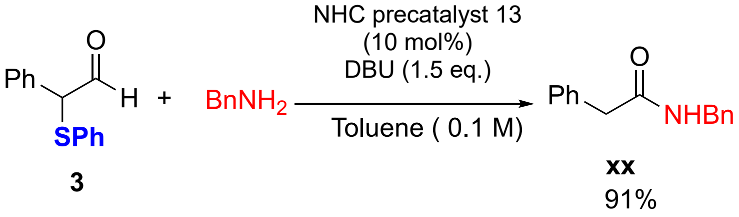

To a flame dried Schlenk tube under N_2 was added a-thiophenylaldehyde (1 equiv.), NHC precatalyst (0.1 equiv.) and anhydrous toluene (0.1 M) and benzylamine (2 equiv.). The reaction was stirred for five minutes before DBU (1.5 eq) was added via Hamiltonian syringe. The reaction was stirred for 16 h under a nitrogen atmosphere. The reaction was washed with 0.1 M HCl, followed by brine and the organics were dried over $MgSO_4$. The solvent was removed under reduced pressure and the crude residue was purified using column chromatography eluting with hexane:EtOAc to give the amide products.

2.6 General procedure: NHC-catalysed formal [4+2] cycloaddition

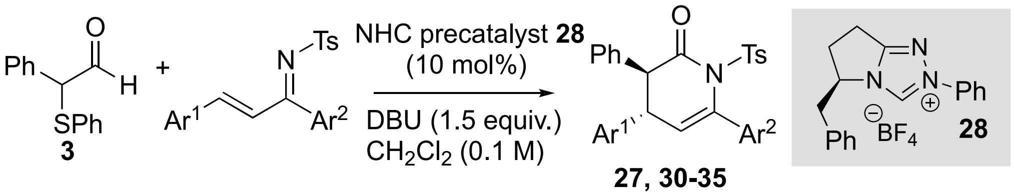

To a flame dried Schlenk flask under N_2 was added α-phenylthioaldehyde (1.5 equiv.), α,β-unsaturated tosyl imine (1 equiv.), NHC precatalyst (0.2 equiv.) and anhydrous CH_2Cl_2 (0.1 M). The reaction was stirred for 5 minutes before DBU (1.5 equiv.) was added. The reaction was stirred under nitrogen for 24 h. The reaction was diluted with CH_2Cl_2 and was washed with 0.1 M HCl. The organic layer was collected and dried over $MgSO_4$ and then concentrated at reduced pressure. The residue was purified by column chromatography (EtOAc:hexane) followed by recrystallisation from EtOAc.

3. Characterisation of compounds.

3.1 α-Phenylthioalcohols

2-methoxy-1-phenyl-2-(phenylthio)ethan-1-ol (2a)

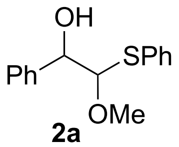

2a

Using general procedure **2.2** with benzaldehyde (0.53 mL, 5.17 mmol) as the selected aldehyde, gave after purification by column chromatography (80:20 hexane:EtOAc) the title compound (as a mixture of both diastereoisomers), (1.04 g, 85%) as a colourless oil. **dr** = 85-15; **IR** ν_{max} (neat); 3300 (OH stretch), 2833, 1181. Spectroscopic data for major diastereomer; ^{1}**H NMR** (400 MHz, $CDCl_3$) $\delta_{H:}$ 3.12 (1H, br s, OH), 3.59 (3H, s, OC*H*$_3$), 4.61 (1H, d, 7.9, C*H*), 4.73 (1 H, d, *J* 7.9, C*H*), 7.20-7.26 (5H, m, Ph*H*), 7.30-7.41 (5H, m, SPh*H*); Selected ^{1}**H NMR** for minor diastereomer; 3.41 (3H, s, O*CH$_3$*), 4.56 (1H, d, *J* 7.09, C*H*), 4.62 (1H, d, *J* 7.11, C*H*) ppm; **HRMS (ESI$^+$)** $C_{15}H_{16}NaO_2S$ $[M+Na]^+$ found 283.0756, 283.0763 required (– 2.5 ppm).

1-methoxy-1-(phenylthio)propan-2-ol (2b)

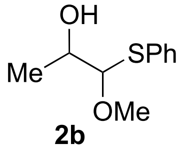

2b

Using general procedure **2.2** with acetaldehyde (0.29 mL, 5.17 mmol) as the selected aldehyde, gave after purification by column chromatography (90:10 hexane:EtOAc) the title compound (as a mixture of both diastereoisomers) (703 mg, 85%) as a colourless oil with data in accordance with literature.[3] **dr** = 60-40; Spectroscopic data for major diastereomer: ^{1}**H NMR**

(400 MHz, $CDCl_3$) δ_H: 1.32 (3H, d, *J* 6.3, *CH_3*), 2.59 (1H, br s, O*H*), 3.55 (3H, s, O*CH_3*), 3.69-3.83 (1H, m, C*H*), 4.33 (1H, d, *J* 7.5, C*H*), 7.18-7.42 (3H, m, SPh*H*), 7.45-7.59 (2H, m, SPh*H*); Selected **^{1}H NMR** for minor diastereomer: 1.29 (3H, d, *J* 6.2, *CH_3*), 3.52 (3H, s, O*CH_3*), 3.69-3.83 (1H, m, C*H*), 4.40 (1H, d, *J* 6.6, C*H*) ppm

1-methoxy-1-(phenylthio)butan-2-ol (2c)

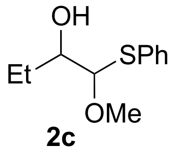

2c

Using general procedure **2.2** with propionaldehyde (0.37 mL, 5.17 mmol) as the selected aldehyde, gave after purification by column chromatography (90:10 hexane:EtOAc) the title compound (as a mixture of both diastereoisomers) (758 mg, 76%) as a colourless oil with data in accordance with literature.[4] **dr** = 56-44; Spectroscopic data for major diastereomer: **^{1}H NMR** (400 MHz, $CDCl_3$) $\delta_{H:}$ 0.98 (3H, t, *J* 7.4, *CH_3*), 1.42-1.61 (2H, m, *CH_2*), 1.91 (1H, dqd, *J* 13.9, 7.5, 3.3, C*H*), 2.39 (1H, br s, O*H*), 3.54 (3H, s, O*CH_3*), 4.43 (1H, d, *J* 7.1, CH), 7.28-7.33 (3H, m, SPh*H*), 7.44-7.55 (2H, m, SPh*H*); Selected **^{1}H NMR** for minor diastereomer: 1.79 (1H, dqd, *J* 13.9, 7.5, 3.3, C*H*), 3.51 (3H, s, O*CH_3*), 4.46 (1H, d, *J* 6.8, C*H*) ppm.

1-methoxy-3-methyl-1-(phenylthio)butan-2-ol (2d)

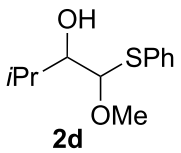

2d

Using general procedure **2.2** with isobutyraldehyde (0.47 mL, 5.17 mmol) as the selected aldehyde, gave after purification by column chromatography (90:10 hexane:EtOAc) the title compound (as a mixture of both diastereoisomers) (660 mg, 62%) as a colourless oil with data in accordance with literature.[5] **dr** = 67-33; Spectroscopic data for major diastereomer; **^{1}H NMR** (400 MHz, $CDCl_3$) δ_H: 0.91 (3H, d, *J* 6.80, CH(*CH_3*)$_2$), 0.95 (3H, d, *J* 7.0, CH(*CH_3*)$_2$), 2.20 (1H, pd, *J* 6.8, 4.0, C*H*(CH_3)$_2$), 2.41(1H, br s, O*H*), 3.44 (1H, dd, *J* 6.9, 4.1, C*H*), 3.55 (3H, s, O*CH_3*), 4.53 (1H, d, *J* 6.90, CH), 7.27-7.34 (3H, m, SPh*H*), 7.48-7.51 (2H, m, SPh*H*); Selected **^{1}H NMR** for minor diastereomer: 0.92 (3H, d, *J* 6.79, CH(*CH_3*)$_2$), 0.96 (3H, d, *J* 6.79,

CH(CH_3)$_2$), 2.04 (1H, pd, *J* 6.8, 4.0, C*H*(CH_3)$_2$), 3.36 (1H, dd, *J* 7.5, 3.8, C*H*), 3.52 (3H, s, OCH_3), 4.54 (1H, d, *J* 7.57, C*H*) ppm

1-methoxy-3,3-dimethyl-1-(phenylthio)butan-2-ol (2e)

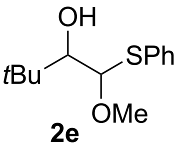

2e

Using general procedure **2.2** with pivaldehyde (0.57 mL, 5.17 mmol) as the selected aldehyde, gave after purification by column chromatography (90:10 hexane:Et_2O) the title compound (as a mixture of both diastereoisomers) (791 mg, 70%) as a colourless oil with data in accordance with literature.[6] **dr** = 67-33; Spectroscopic data for major diastereomer; **^{1}H NMR** (400 MHz, $CDCl_3$) δ_H: 0.96 (9H, s, C(CH_3)$_3$), 2.36 (1H, br s, O*H*), 3.51 (3H, s, OCH_3), 3.56-3.63 (1H, m, C*H*), 4.80-4.86 (1H, m, C*H*), 7.27-7.39 (3H, m, SPh*H*), 7.54 (2H, dt, *J* 7.9, 1.4, SPh*H*); Selected **^{1}H NMR** for minor diastereomer; 0.99 (9H, s, C(CH_3)$_3$), 2.78 (1H, br s, O*H*), 3.18 (1H, dd, *J* 8.0, 1.5, C*H*), 3.53 (3H, s, OCH_3), 4.61 (1H, d, *J* 8.0, CH), 7.25 -7.40 (3H, m, SPh*H*), 7.47-7.57 (2H, m, SPh*H*).

1-methoxy-1-(phenylthio)hexan-2-ol (2f)

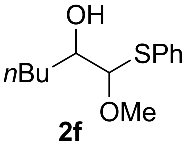

2f

Using general procedure **2.2** with valeraldehyde (0.55 mL, 5.17 mmol) as the selected aldehyde, gave after purification by column chromatography (90:10 hexane:EtOAc) the title compound (as a mixture of both diastereoisomers), (791 mg, 70%) as a colourless oil. **dr** = 57:43; **IR** ν_{max} (neat); 3300 (OH stretch) 1423, 2860. Spectroscopic data for major diastereoisomer; **^{1}H NMR** (400 MHz, $CDCl_3$) δ_H: 0.92 (3H, t, *J* 7.20, CH_3), 1.29-1.37 (2H, m, CH_2), 1.43 -1.55 (2H, m, CH_2), 1.70-1.80 (1H, m, CH_2), 1.84-1.92 (1H, m, CH_2), 2.54 (1H, brs, O*H*), 3.53 (3H, s, OCH_3), 3.63 (1H, ddd, *J* 8.7, 7.0, 3.0, C*H*), 4.41 (1H, d, *J* 7.1, C*H*), 7.26-7.33 (3H, m, SPh*H*), 7.45-7.53 (2H, m, SPh*H*); Selected **^{1}H NMR** for minor diastereomer; 3.50 (3H, s, OCH_3), 3.57 (1H, ddd, *J* 8.5, 6.5, 3.1, C*H*), 4.46 (1H, d, *J* 6.5, C*H*) ppm; **HRMS (ESI$^+$)** $C_{13}H_{20}NaO_2S$ [M+Na]$^+$ found 263.1069, 263.1082 required (-4.9 ppm).

1-cyclohexyl-2-methoxy-2-(phenylthio)ethan-1-ol (2g)

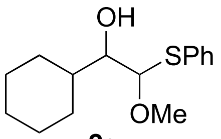

2g

Using general procedure **2.2** with cyclohexanecarboxaldehyde (0.63 mL, 5.17 mmol) as the selected aldehyde, gave after purification by column chromatography (90:10 hexane:EtOAc) the title compound (as a mixture of both diastereoisomers) (939 mg, 75%) as a colourless oil with data in accordance with literature.[3] **dr** = 83:17; Spectroscopic data for major diastereomer; **^{1}H NMR** (400 MHz, $CDCl_3$) δ_H: 1.10-1.30 (5H, m, cyclohexyl C*H*), 1.60-1.79 (6H, m, cyclohexyl C*H*), 2.66 (1H, brs, O*H*), 3.47 (1H, dt, *J* 6.6, 4.1, C*H*), 3.54 (3H, s, O*CH$_3$*), 4.59 (1H, d, *J* 6.2 Hz, C*H*), 7.27-7.34 (3H, m, SPh*H*), 7.46-7.52 (2H, m, SPh*H*); Selected **^{1}H NMR** for minor diastereomer 3.52 (3H, s, O*CH$_3$*) ppm.

1-methoxy-2-phenyl-1-(phenylthio)propan-2-ol (2h)

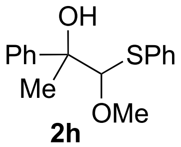

2h

Using general procedure **2.2** with acetophenone (0.60 mL, 5.17 mmol) as the selected ketone, gave after purification by column chromatography (90:10 hexane:EtOAc) the title compound (774 mg, 60%) as a colourless oil with data in accordance with literature[7] **^{1}H NMR** (400 MHz, $CDCl_3$) *δ*H: 1.76 (3H, s, CH_3), 3.19 (1H, brs, O*H*), 3.48 (3H, s, O*CH$_3$*), 4.78 (1H, s, CH), 7.27 (5H, s, Ar*H*), 7.30 - 7.42 (3H, m, SPh*H*), 7.62-7.68 (2H, m, SPh*H*) ppm.

3.2 α-Phenylthioaldehydes

2-phenyl-2-(phenylthio)acetaldehyde (3)

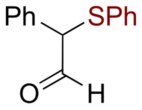

3

Using general procedure **2.3**, work-up B) and α-phenylthioalcohol **2a** (521 mg, 2 mmol), gave the title compound was obtained as a yellow oil (388 mg, 85%). **^{1}H NMR** (400 MHz, $CDCl_3$)

$\delta_{H:}$ 4.78 (1H, d, *J* 4.6, C*H*), 7.19-7.61 (10H, m, Ar*H*), 9.60 (1H, d, *J* 4.6 Hz, C*H*O) data in accordance with literature.[8]

3.2.1 Synthesis of unsubstituted α-phenylthioaldehyde 4

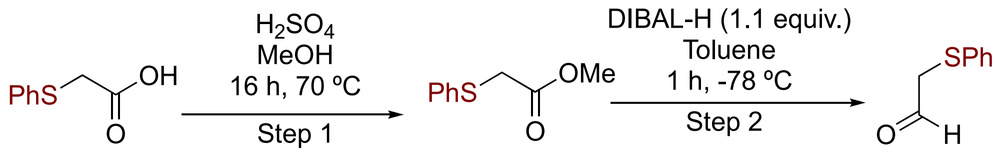

4a **4b** **4**

Step 1: Following a modification of the procedure outlined by Heavner[9], to a flame dried round bottom flask was added carboxylic acid **4a** (1.50 g, 8.93 mmol), methanol (50 mL) and concentrated sulfuric acid (5 mL). The flask was fitted with a reflux condenser and heated to 70 ˚C for 16 h. The reaction was allowed to cool to rt and was diluted with CH_2Cl_2 (50 mL). The solution was washed with $NaHCO_3$ (2 × 50 mL) and then with deionised water (100 mL). The organic layers were combined and dried over $MgSO_4$ and the solvent was concentrated at reduced pressure to give the methyl ester **4b** as a colourless oil (1.18g, 6.48 mmol, 73%), which was used without further purification.

methyl 2-(phenylthio)acetate (4b)

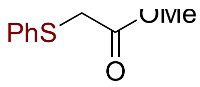

4b

^{1}H NMR (400 MHz, $CDCl_3$) $\delta_{H:}$ 3.68 (2H, s, CH_2), 3.74 (3H, s, OCH_3), 7.21-7.30 (1H, m, ArH), 7.31-7.35 (2H, m, ArH), 7.39-7.48 (2H, m, ArH). Data in accordance with literature.[10]

Step 2: To a flame dried two-neck round bottom flask under N_2 was added methyl ester **4.125** (1.0 g, 5.40 mmol), and anhydrous toluene (40 mL). The reaction was cooled to -78 ˚C using a dry ice/acetone bath. Dropwise DIBAL-H (1 M in hexanes, 3.78 mL, 7.56 mmol) was added via syringe. The reaction was stirred at -78 ˚C for one hour. The reaction was quenched by the addition of anhydrous methanol (4 mL) before a saturated solution of Rochelle's salt was added (40 mL). The mixture was stirred vigorously for 15 minutes. The mixture was extracted with Et_2O (3 × 30 mL). The organic layers were washed with brine and dried over $MgSO_4$. The solvent was removed at reduced pressure to give the phenylthioaldehyde **4** as a yellowish oil.

The compound was purified using flash column chromatography eluting with EtOAc:Pet ether (5:95). Product **4** obtained as a colourless oil (450 mg, 2.96 mmol, 55%).

2-(phenylthio)acetaldehyde (4)

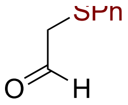

4

^{1}H NMR (400 MHz, $CDCl_3$) $\delta_{H:}$ 3.62 (2H, d, *J* 3.2, CH_2), 7.24-7.40 (5H, m, SPh*H*), 9.57 (1H, t, *J* 3.2, C*H*O) data in accordance with the literature[11]

2-(phenylthio)propanal (5)

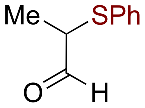

5

Using general procedure **2.3**, work-up A) and α-phenylthioalcohol **2b** (397 mg, 2 mmol), gave the title compound a colourless oil (133 mg, 40%). **^{1}H NMR** (400 MHz, $CDCl_3$) $\delta_{H:}$ 1.42 (3H, d, *J* 7.0, CH_3), 3.66 (1H, qd, *J* 7.0, 3.2, C*H*), 7.27-7.37 (3H, m, SPh*H*), 7.39-7.47 (2H, m, SPh*H*), 9.47 (1H, d, *J* 3.2 Hz, C*H*O) data in accordance with literature.[12]

2-(phenylthio)butanal (6)

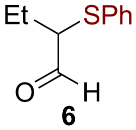

6

Using general procedure **2.3**, work-up A) and α-phenylthioalcohol **2c** (425 mg, 2 mmol), gave the title compound as a yellow oil (169 mg, 47%). **^{1}H NMR** (400 MHz, $CDCl_3$) $\delta_{H:}$ 1.12 (3H, t, *J* 7.4, *CH_3*), 1.66-1.81 (1H, m, *CH_2*), 1.81-1.97 (1H, m, *CH_2*), 3.48 (1H, ddd, *J* 7.7, 6.9, 4.1, C*H*), 7.24-7.37 (3H, m, SPh*H*), 7.37-7.46 (2H, m, SPh*H*), 9.42 (1H, d, *J* 4.1, C*H*O), data in accordance with the literature.[13]

2-(phenylthio)hexanal (7)

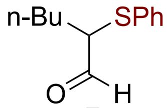

7

Using general procedure **2.3**, work-up a) and α-phenylthioalcohol **2f** (480 mg, 2 mmol), gave the title compound was obtained as a yellow oil (192 mg, 46%). **^{1}H NMR** (400 MHz, $CDCl_3$) $\delta_{H:}$ 0.94 (3H, t, *J* 7.2, CH_3), 1.34-1.60 (4H, m, 2 × CH_2), 1.64-1.76 (1H, m, CH_2), 1.77-1.91 (1H, m, CH_2), 3.54 (1H, ddd, *J* 7.9, 6.9, 4.3, CH), 7.29-7.35 (3H, m, SPh*H*), 7.36-7. 42 (2H, m, SPh*H*), 9.39 (1H, d, *J* 4.3, C*H*O). data in accordance with literature.[14]

3-methyl-2-(phenylthio)butanal (8)

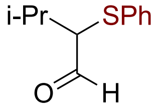

8

Using general procedure **2.3**, work-up a) and α-phenylthioalcohol **2d** (453 mg, 2 mmol), gave the title compound was obtained as a colourless oil (323 mg, 83%). **^{1}H NMR** (400 MHz, $CDCl_3$) $\delta_{H:}$ 1.12 (3H, d, *J* 6.8, CH_3) 1.22 (3H, d, *J* 6.7, CH_3), 2.13 (1H, dh, *J* 8.6, 6.7, C*H*), 3.31 (1H, dd, *J* 8.5, 5.4, C*H*), 7.26-7.35 (3H, m, SPh*H*), 7.38 -7.44 (2H, m, SPh*H*), 9.37 (1H, d, *J* 5.4, C*H*O). Data in accordance with literature.[13]

3,3-dimethyl-2-(phenylthio)butanal (9)

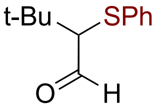

9

Using general procedure **2.3**, work-up a) and α-phenylthioalcohol **2e** (481 mg, 2 mmol), gave the title compound was obtained as a colourless oil (313 mg, 75%). **^{1}H NMR** (400 MHz, $CDCl_3$) $\delta_{H:}$ 1.19 (9H, s, $C(CH_3)_3$), 3.27 (1H, d, *J* 6.5, CH), 7.22-7.36 (3H, m, SPh*H*), 7.37-7.44 (2H, m, SPh*H*), 9.49 (1H, d, *J* 6.5, CHO). data in accordance with literature.[15]

2-cyclohexyl-2-(phenylthio)acetaldehyde (10)

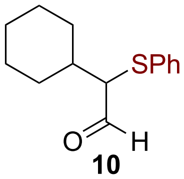

10

Using general procedure **2.3**, work-up a) and α-phenylthioalcohol **2g** (533 mg, 2 mmol), gave the title compound was obtained as a colourless oil (70.3 mg, 15%). **^{1}H NMR** (400 MHz, $CDCl_3$) $\delta_{H:}$ 1.09-1.43 (5H, m, cyclohexyl H), 1.64-1.91 (5H, m, cyclohexyl H), 2.12-2.17 (1H, m, C*H*), 3.37 (1H, dd, *J* 8.70, 5.60, C*H*), 7.23-7.43 (5H, m, SPh*H*), 9.34 (1H, d, *J* 5.60, C*H*O) ppm. Data in accordance with literature.[8]

2-phenyl-2-(phenylthio)propanal (11)

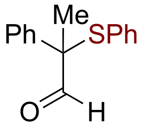

11

Using general procedure **2.3**, work-up a) and α-phenylthioalcohol **2h** (545 mg, 2 mmol), the title compound was obtained as a yellow oil (388 mg, 80%). **^{1}H NMR** (400 MHz, $CDCl_3$) $\delta_{H:}$ 1.59 (3H, s, CH_3), 7.29-7.48 (8H, m, ArH), 7.50-7.56 (2H, m, ArH), 9.70 (1H, s, C*H*O) data in accordance with literature[5]

3.3 Thiolesters

***S*-phenyl 2-phenylethanethioate (12)**

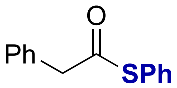

12

Following the general **2.4**, α-phenylthioaldehyde **2** (114 mg, 0.5 mmol), NHC precatalyst **16** (18.8 mg, 0.05 mmol), triethylamine (6.9 µL, 0.05 mmol) and toluene (5 mL) with a reaction time of 0.25 h, gave the thiolester **12** as a yellow residue which was purified by column chromatography (2:98 EtOAc:Hexane) to give the title compound as a colourless oil (112 mg, 98%). **^{1}H NMR** (400 MHz, $CDCl_3$) $\delta_{H:}$ 3.93 (2H, s, CH_2), 7.31-7.40 (10H, m, ArH) spectroscopic data in accordance with literature[16]

***S*-phenyl ethanethioate (17)**

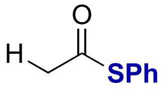

17

Following the general procedure **2.4**, α-phenylthioaldehyde **4** (76.1 mg, 0.5 mmol), NHC precatalyst **16** (18.8 mg, 0.05 mmol), triethylamine (6.9 μL, 0.05 mmol) and toluene (5 mL) with a reaction time of 1 h gave the thiolester **17** as a yellow residue which was purified by flash column chromatography (2:98 EtOAc:Hexane) to give the title compound as a colourless oil (73.8 mg, 97%). **^{1}H NMR** (400 MHz, $CDCl_3$) $\delta_{H:}$ 2.45 (3H, s, CH_3), 7.45 (5H, s, SPh*H*) data in accordance with literature[17]

***S*-phenyl propanethioate (18)**

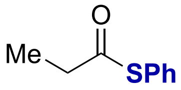

18

Following the general procedure **2.4**, α-phenylthioaldehyde **5** (30.0 mg, 0.18 mmol), NHC precatalyst **16** (6.79 mg, 0.018 mmol), triethylamine (2.5 μL, 0.018 mmol) and toluene (1.8 mL) with a reaction time 1 h, gave the thiolester **18** as a yellow residue which was purified by column chromatography (2:98 EtOAc:Hexane) to give the title compound as a colourless oil (29 mg, 97%). **^{1}H NMR** (400 MHz, $CDCl_3$) $\delta_{H:}$ 1.25 (3H, t, *J* 7.5, CH_3), 2.71 (2H, q, *J* 7.5, CH_2), 7.43(5H, s, SPh*H*). Spectroscopic data in accordance with literature.[18]

***S*-phenyl butanethioate (19)**

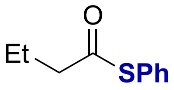

19

Following the general procedure **2.4**, α-phenylthioaldehyde **6** **(**90.1 mg, 0.5 mmol), NHC precatalyst **16** (18.8 mg, 0.05 mmol), triethylamine (6.9 μL, 0.05 mmol) and toluene (5 mL) with a reaction time of 1 h gave the thiolester **19** as a yellow residue which was purified by flash column chromatography (2:98 EtOAc:Hexane) to give the title compound as a colourless oil (86.5 mg, 96 %). **^{1}H NMR** (400 MHz, $CDCl_3$) $\delta_{H:}$ 1.03 (3H, d, *J* 7.4 Hz, CH_3), 1.78 (2H, h,

J 7.40 Hz, CH_2), 2.67 (2H, dd, J 7.7, 7.1 Hz, CH_2), 7.34-7.48 (5H, s, SPh*H*). Spectroscopic data in accordance with literature.[19]

***S*-phenyl hexanethioate (20)**

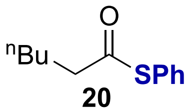

20

Following the general procedure **2.4**, α-phenylthioaldehyde **7** (104 mg, 0.5 mmol), NHC precatalyst **16** (18.8 mg, 0.05 mmol), triethylamine (6.9 µL, 0.05 mmol) and toluene (5 mL) with a reaction time of 1 h gave the thiolester **20** as a yellow residue which was purified by flash column chromatography (2:98 EtOAc:Hexane) to give the title compound as a colourless oil (100.1 mg, 97%). ^{1}**H NMR** (400 MHz, $CDCl_3$) $\delta_{H:}$ 0.91 (3H, app t, CH_3), 1.31-1.42 (4H, m, 2 × CH_2), 1.68-1.76 (2H, m, CH_2), 2.65 (2H, app t, CH_2), 7.41 (5H, s, SPh*H*). Spectroscopic data in accordance with the literature.[20]

***S*-phenyl 3-methylbutanethioate (21)**

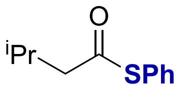

21

Following the general procedure **2.4** α-phenylthioaldehyde **8** (82.0 mg, 0.42 mmol), NHC precatalyst **D** (15.8 mg, 0.042 mmol), triethylamine (5.9 µL, 0.042 mmol) and toluene (4.2 mL) with a reaction time 2 h, gave the thiolester **21** as a yellow residue which was purified by flash column chromatography (2:98 EtOAc:Hexane) to give the title compound as a colourless oil (80.4 mg, 98 %). ^{1}**H NMR** (400 MHz, $CDCl_3$) $\delta_{H:}$ 1.01 (6H, d, J 6.65, 2 × $CH(CH_3)_2$) 2.22 (1H, h, J 6.75, $CH(CH_3)_2$), 2.54 (2H, d, J 7.10, CH_2), 7.41 (5H, s, SPh*H*). Spectroscopic data in accordance with literature.[21]

***S*-phenyl 2-cyclohexylethanethioate (22)**

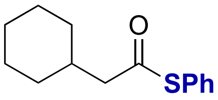

22

Following the general procedure **2.4**, α-phenylthioaldehyde **10** (100 mg, 0.43 mmol), NHC precatalyst **16** (16.0 mg, 0.043 mmol), triethylamine (6.0 μL, 0.043 mmol) and toluene (4.3 mL) with a reaction time of 4 h gave the thiolester **22** as a yellow residue which was purified by flash column chromatography (2:98 EtOAc:Hexane) to give the title compound as a colourless oil (87.0 mg, 87%). **^{1}H NMR** (400 MHz, $CDCl_3$) $\delta_{H:}$ 1.04 (2H, qd, *J* 12.3, 3.4, CH_2), 1.18 (1H, qt, *J* 12.7, 3.3, CH), 1.30 (2H, qt, *J* 12.4, 3.3, CH_2), 1.64-1.78 (3H, m, CH), 1.78-1.85 (2H, m, CH_2), 1.92 (1H, ttt, *J* 10.8, 7.0, 3.5, CH), 2.56 (2H, d, *J* 7.0, CH_2), 7.43 (5H, s, SPh*H*). Spectroscopic data in accordance with the literature;[22] **HRMS** ESI$^+$ $[M+Na]^+$ $C_{14}H_{18}OSNa$ 257.0976 required, 257.0966 found (-3.89 ppm).

***S*-phenyl 3,3-dimethylbutanethioate (23)**

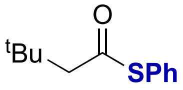

23

Following the general procedure **2.4**, α-phenylthioaldehyde **9** (120 mg, 0.58 mmol), NHC precatalyst **16** (21.6 mg, 0.058 mmol), triethylamine (8.0 μL, 0.058 mmol) and toluene (5.8 mL) with a reaction time of 6 h gave the thiolester **23** as a yellow residue which was purified by flash column chromatography (2:98 EtOAc:Hexane) to give the title compound as a colourless oil (116 mg, 97%). **^{1}H NMR** (400 MHz, $CDCl_3$) $\delta_{H:}$ 1.08 (9H, s, $C(CH_3)_3$), 2.55 (2H, s, CH_2), 7.41 (5H, s, SPh*H*). Spectroscopic data in accordance with literature.[20]

3.4 Esters

benzyl 2-phenylacetate (24)

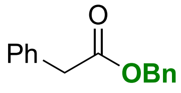

24

Following general procedure **2.5**, α-phenylthioaldehyde **3** (68.5 mg, 0.3 mmol), NHC precatalyst **13** (9.51 mg, 0.03 mmol), NEt_3 (62.7 μL, 0.45 mmol), benzyl alcohol (62.1 μL, 0.6 mmol) and anhydrous toluene (3 mL) after column chromatography (Hexane:EtOAc 90:10) gave the title compound **24** as a colourless oil (65.8 mg, 97%) with spectroscopic data in accordance with the literature.[23] **^{1}H NMR** (400 MHz, $CDCl_3$) δ_H: 3.72 (2H, s, CH_2), 5.18 (2H, s, CH_2), 7.23-7.41 (10H, m, Ar*H*).

benzyl 2-phenylacetate (24)

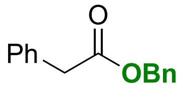

24

Following general procedure **2.5**, α-phenylthioaldehyde **3** (68.5 mg, 0.3 mmol), NHC precatalyst **16** (11.3 mg, 0.03 mmol), DBU (67.3μL, 0.45 mmol), benzyl alcohol (62.1 μL, 0.6 mmol) and anhydrous toluene (3 mL) after column chromatography (Hexane:EtOAc 90:10) gave the title compound as a colourless oil (66.5 mg, 98%) with spectroscopic data in accordance with the literature.[23] **^{1}H NMR** (400 MHz, $CDCl_3$) δ_H: 3.72 (2H, s, CH_2), 5.18 (2H, s, CH_2), 7.23-7.41 (10H, m, Ar*H*).

benzyl propionate (25)

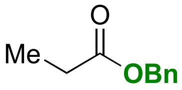

25

Following general procedure **2.5**, α-phenylthioaldehyde **5** (59.5 mg, 0.3 mmol), NHC precatalyst **13** (9.51 mg, 0.03 mmol), DBU (67.3 μL, 0.45 mmol), benzyl alcohol (62.1 μL, 0.6 mmol) and anhydrous toluene (3 mL) after column chromatography (Hexane:EtOAc 90:10) gave the title compound **25** as a colourless oil (46.3 mg, 94%) with spectroscopic data in accordance with the literature.[24] **^{1}H NMR** (400 MHz, $CDCl_3$) $\delta_{H:}$ 1.20 (3H, t, *J* 7.60, CH_3), 2.42 (2H, q, *J* 7.6, CH_2), 5.16 (2H, s, CH_2), 7.38 (5H, s, ArH).

methyl propionate (27)

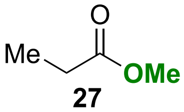

27

Following general procedure **2.5**, α-phenylthioaldehyde **5** (59.5 mg, 0.3 mmol), NHC precatalyst **13** (9.51 mg, 0.03 mmol), DBU (67.3 μL, 0.45 mmol), MeOH (μL, 0.6 mmol) and anhydrous toluene (3 mL) after column chromatography (Hexane:EtOAc 90:10) gave the title compound **27** as a colourless oil (25.1 mg, 95%) with spectroscopic data in accordance with the literature.[25]

3.5 Amide

***N*-benzyl-2-phenylacetamide (25a)**

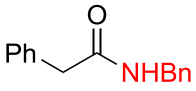

25a

Following general procedure **2.6**, a-thiophenylaldehyde **4.98** (45.7 mg, 0.2 mmol), NHC precatalyst **13** (7.53 mg, 0.02 mmol), DBU (44.9 µL, 0.3 mmol), benzylamine (43.7 µL, 0.4 mmol) and anhydrous toluene (2 mL) gave the title compound **25a** as a white solid (41 mg, 91%) with spectroscopic data in accordance with the literature.[26] **^{1}H NMR** (400 MHz, $CDCl_3$) $\delta_{H:}$ 3.65 (2H, s, CH_2), 4.44 (2H, d, *J* 5.8, CH_2), 5.75 (1H, br s, N*H*), 7.20 (2H, d, *J* 6.83, Ar*H*), 7.26-7.39 (8H, m, Ar*H*).

3.5 Dihydropyridinones

(3*S*,4*S*)-6-(4-fluorophenyl)-3,4-diphenyl-1-tosyl-3,4-dihydropyridin-2(1*H*)-one (29)

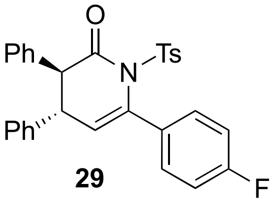

29

Following general procedure **2.6**, α-phenylthioaldehyde **3** (50.0 mg, 0.219 mmol), NHC precatalyst **30** (9.16 mg, 0.0292 mmol), DBU (33 µL, 0.219 mmol), α,β-unsaturated -unsaturated tosyl imine (55.4 mg, 0.146 mmol) and anhydrous toluene (1.5 mL) gave after column chromatography (Hexane:EtOAc 90:10) and recrystallisation (CH_2Cl_2/EtOAc) the title compound **29** as a white solid (55.2 mg, 76%); **mp** 171 -172 ˚C; **$[\alpha]^{20}_D$** = +18.3 (c = 1.5 in $CHCl_3$) **^{1}H NMR** (400 MHz, $CDCl_3$) $\delta_{H:}$ 2.46 (3H, s, CH_3), 3.90 (1H, d, *J* 10.6, CH), 4.02 (1H, dd, *J* 10.6, 4.2, CH), 5.97 (1H, d, *J* 4.2, CH), 6.77 – 6.84 (2H, m, Ar*H*), 7.00 – 7.10 (4H, m, Ar*H*), 7.11 – 7.23 (6H, m, Ar*H*), 7.29 (2H, d, *J* 8.7, Ar*H*), 7.39 (2H, dd, *J* 8.8, 5.5, Ar*H*), 7.83 (2H, d, *J* 8.4, Ar*H*) ppm; **^{13}C NMR {^{1}H}** (101 MHz, $CDCl_3$) δ 21.7 (CH_3), 45.1 (CH), 58.9 (CH), 115.4 (ArC), 115.6 (ArC), 122.7 (CH), 127.2 (ArC), 127.4 (ArC), 127.8 (ArC), 127.9 (ArC), 128.4 (ArC), 128.7 (ArC), 129.2 (ArC), 129.4 (ArC), 133.2 (ArC), 136.3 (ArC), 139.2 (ArC), 139.7 (ArC), 145.2 (ArC), 161.6 (ArC), 164.0 (ArC), 173.0 (C=O) ppm; **^{19}F NMR** (282 MHz, $CDCl_3$) $\delta_{F:}$ -112.8 (ArF); **IR ν_{max} (film)** 1717 (C=O amide); **HRMS (ESI, m/z):** $[M+H]^+$

$C_{30}H_{26}O_3NFS$ 498.1539 required, 498.1534 found (-1.00 ppm) also found $[M+K]^+$ $C_{30}H_{25}O_3NFSK$ 536.1098 required, 536.1090 found (- 1.49 ppm); **Chiral HPLC analysis**: Chiralpak OD-H (20:80 IPA : hexane, flow rate 1 mL/min, 254 nm, 30 ˚C) t_R 8.5 mins (major) and 16.7 (minor) 98:2 e.r.

Note: The racemic compounds were obtained following general procedure **2.6** using achiral triazolium precatalyst **13**. Triazolium precatalyst **16** resulted in formation of the thiolester **12**.

(3*S*,4*S*)-6-(4-fluorophenyl)-3,4-diphenyl-5-tosyl-3,4-dihydropyridin-2(1*H*)-one (31)

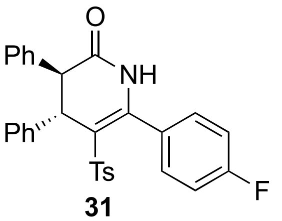

31

A solution of the **(3*S*,4*S*)-6-(4-fluorophenyl)-3,4-diphenyl-1-tosyl-3,4-dihydropyridin-2(1*H*)-one (29)**, (80 mg, 0.17 mmol) in degassed CH_2Cl_2 (0.1M) was irradiated with UV light at 365 nm (internal temperature within the UV chamber recorded as 30 °C) for 16h. The solvent was removed under reduced pressure and the crude reaction mixture purified by column chromatography in the solvent system (CH_2Cl_2/Et_2O, 95:5) gave the title compound **31** (24 mg, 29%, >95:5 dr) as an off white solid. **mp** 200-201°C; $[\alpha]^{20}_D$ = +25.6 (c = 0.7 in $CHCl_3$) **^{1}H NMR** (400 MHz, $CDCl_3$) δ 7.47 – 7.29 (m, 3H, *Ar-H*), 7.19 (brs, 1H, *NH*), 7.02 (t, *J* = 8.4 Hz, 2H, *Ar-H*), 6.81 (d, *J* = 8.0 Hz, 2H, *Ar-H*), 6.65 (d, *J* = 8.2 Hz, 2H, *Ar-H*), 4.81 (d, *J* = 1.5 Hz, 1H, CH), 4.09 (s, 1H, *CH*), 2.28 (s, 3H) ppm; **^{13}C NMR {^{1}H}** (126 MHz, $CDCl_3$) δ 169.2 (C=O), 164.8(Ar*C*), 162.8(Ar*C*), 144.7(Ar*C*), 143.4(Ar*C*), 139.6(Ar*C*), 138.1(Ar*C*), 136.9(Ar*C*), 131.3(Ar*C*), 129.7(Ar*C*), 129.3(Ar*C*), 129.3(Ar*C*), 129.2(Ar*C*), 129.0(Ar*C*), 128.8(Ar*C*), 128.2(Ar*C*), 128.1(Ar*C*), 128.1(Ar*C*), 127.9(Ar*C*), 127.8(Ar*C*), 127.3(Ar*C*), 127.2(Ar*C*), 127.1(Ar*C*), 126.9(Ar*C*), 119.7(Ar*C*), 115.7(Ar*C*), 115.5(Ar*C*), 53.7(*C*H), 47.1(*C*H), 21.5(*C*H_3) ppm; **^{19}F NM** (377 MHz, $CDCl_3$) δ -108.9. (ArF); **IR ν_{max} (film):** 1696 (C=O amide), 3248 (N-H), 1633, 1145; **HRMS (ESI, m/z)**: calcd. for $C_{30}H_{24}FNO_3S$ Na^+ 520.1353, found 520.1349 (-2.72 ppm); **Chiral HPLC analysis**: Chiralpak IA (30:70 IPA: hexane, flow rate 1 mL/min, 211 nm, 30 ˚C) t_R 19.1 min (major) and 17.3 min (minor) 5:95 e.r.

(3*S*,4*S*)-6-(4-bromophenyl)-3,4-diphenyl-1-tosyl-3,4-dihydropyridin-2(1*H*)-one (32)

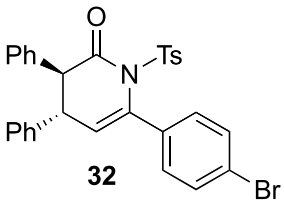

32

Following general procedure **2.6**, α-phenylthioaldehyde **3** (67 mg, 0.3 mmol), NHC precatalyst **30** (14.6 mg, 0.02 mmol), DBU (44.7 μL, 0.3 mmol), corresponding α,β-unsaturated tosyl imine (88 mg , 0.2 mmol) and anhydrous CH_2Cl_2 (2 mL) gave after column chromatography (hexane:EtOAc 90:10) the title compound **32** as a white solid (87 mg, 78%), **mp** 86-88 °C; **$[\alpha]^{20}_D$** = +152.5 (c = 0.3 in $CHCl_3$); **<u>^{1}H NMR</u>** (500 MHz, $CDCl_3$) δ 7.85 (d, *J* = 8.4 Hz, 2H, Ar*H*), 7.51 (d, *J* = 8.4 Hz, 2H, Ar*H*), 7.30 (dd, *J* = 8.2, 6.2 Hz, 2H, Ar*H*), 7.20 – 7.10 (m, 4H, Ar*H*), 7.03 (dd, *J* = 7.8, 1.7 Hz, 6H, Ar*H*), 6.79 (dd, *J* = 6.6, 2.9 Hz, 2H, Ar*H*), 6.02 (d, *J* = 4.3 Hz, 1H, CH), 4.01 (dd, *J* = 10.6, 4.3 Hz, 1H, CH), 3.89 (d, *J* = 10.6 Hz, 1H, CH), 2.47 (s, 3H, C*H*$_3$) ppm; **^{13}C NMR {^{1}H}** (126 MHz, $CDCl_3$) δ 172.8 (C=O), 145.39(Ar*C*), 139.6(Ar*C*), 139.2(Ar*C*), 136.2(Ar*C*), 136.1(Ar*C*), 131.6(Ar*C*), 129.5(Ar*C*), 129.2(Ar*C*), 128.7(Ar*C*), 128.4(Ar*C*), 127.7(Ar*C*), 127.5(Ar*C*), 127.4(Ar*C*), 127.2(Ar*C*), 123.2(Ar*C*), 122.5(=CH), 58.7(CH), 45.0(CH), 21.7(CH_3) ppm; **IR ν_{max} (film)**: 1718 cm^{-1} (C=O amide); **HRMS (ESI, m/z)**: calcd. for $C_{30}H_{24}BrNO_3SH^+$ 558.0734, found 558.0733 (-0.01 ppm); **HPLC analysis**: 95:5 e.r., (CHIRALCEL OD-H column, *n*-hexane/*i*-PrOH = 80/20, flow rate = 1 mL/min, λ = 211 nm, 30 °C, t_{major} = 9.4 min, t_{minor} = 20.9 min)

(3*S*,4*S*)-4,6-bis(4-chlorophenyl)-3-phenyl-1-tosyl-3,4-dihydropyridin-2(1*H*)-one (33)

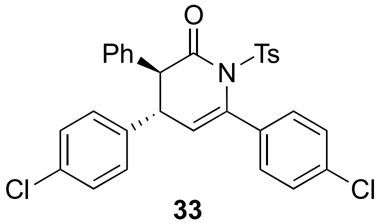

33

Following general procedure **2.6**, α-phenylthioaldehyde **3** (67 mg, 0.3 mmol), NHC precatalyst **30** (14.6 mg, 0.02 mmol), DBU (44.7 μL, 0.3 mmol), corresponding α,β-unsaturated tosyl imine (84 mg , 0.2 mmol) and anhydrous CH_2Cl_2 (2 mL) gave after column chromatography (Heaxane:EtOAc 90:10) the title compound **33** as a white solid (84.3 mg, 77%);**mp** 81-83 °C;

[α]^{20}$_D$ = +53.8 (c = 1.5 in $CHCl_3$); **^{1}H NMR** (500 MHz, $CDCl_3$) δ 7.83 (d, *J* = 8.0 Hz, 2H, Ar*H*), 7.35 (m, 4H, Ar*H*), 7.29 (d, *J* = 8.1 Hz, 2H, Ar*H*), 7.19 – 7.07 (m, 5H, Ar*H*), 6.96 (d, *J* = 8.0 Hz, 2H, Ar*H*), 6.80 (dd, *J* = 6.7, 3.0 Hz, 2H, Ar*H*), 5.95 (d, *J* = 4.3 Hz, 1H, C*H*), 4.01 (dd, *J* = 10.5, 4.3 Hz, 1H, C*H*), 3.86 (d, *J* = 10.4 Hz, 1H, C*H*), 2.47 (s, 3H, C*H₃*) ppm; **^{13}C NMR {^{1}H}** (126 MHz, $CDCl_3$) δ 172.6(C=O), 145.4(Ar*C*), 139.5(Ar*C*), 138.2(Ar*C*) (Ar*C*) (Ar*C*), 136.1(Ar*C*), 135.8(Ar*C*), 135.4(Ar*C*), 134.5(Ar*C*), 133.1(Ar*C*), 129.5(Ar*C*), 129.2(Ar*C*), 129.0(Ar*C*), 128.8(Ar*C*), 128.7(Ar*C*), 128.6(Ar*C*), 127.6(Ar*C*), 127.2(Ar*C*), 122.4(CH), 58.6 (*C*H), 44.4 (CH), 21.7 (CH_3) ppm; **IR ν_{max} (film)**: 1716 cm^{-1} (C=O amide); **HRMS (ESI, m/z)**: calcd. for $C_{30}H_{23}Cl2NO_3SH^+$ 548.0849, found 548.0855(1.19 ppm); **HPLC analysis**: 96:4 e.r., (CHIRALCEL OD-H column, *n*-hexane/*i*-PrOH = 80/20, flow rate = 1 mL/min, λ = 211 nm, 30 °C, t_{major} = 10.1 min, t_{minor} = 23.9 min)

(3*S*,4*S*)-6-(4-chlorophenyl)-3-phenyl-4-(*p*-tolyl)-1-tosyl-3,4-dihydropyridin-2(1*H*)-one (34)

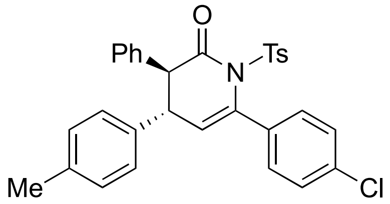

34

Following general procedure **2.6**, α-phenylthioaldehyde **3** (67 mg, 0.3 mmol), NHC precatalyst **30** (14.6 mg, 0.02 mmol), DBU (44.7 μL, 0.3 mmol), corresponding α,β-unsaturated tosyl imine (82 mg , 0.2 mmol) and anhydrous CH_2Cl_2 (2 mL) gave after column chromatography (hexane:EtOAc 90:10) the title compound **34** as a white solid (67.2 mg, 64%); **mp** 80-82 °C **[α]^{20}$_D$** = +45.2 (c = 0.7 in $CHCl_3$); **^{1}H NMR** (500 MHz, $CDCl_3$) δ 7.85 (d, *J* = 7.9 Hz, 2H, Ar*H*), 7.42 – 7.32 (m, 4H, Ar*H*), 7.29 (d, *J* = 8.0 Hz, 2H, Ar*H*), 7.17 – 7.12 (m, 3H, Ar*H*), 6.98 (d, *J* = 7.7 Hz, 2H, Ar*H*), 6.92 (d, *J* = 7.7 Hz, 2H, Ar*H*), 6.81 (dd, *J* = 6.7, 3.0 Hz, 2H, Ar*H*), 5.99 (d, *J* = 4.3 Hz, 1H, C*H*), 3.99 (dd, *J* = 10.4, 4.3 Hz, 1H, C*H*), 3.89 (d, *J* = 10.4 Hz, 1H, C*H*), 2.47 (s, 3H, *C*H_3), 2.25 (s, 3H, *C*H_3) ppm; **^{13}C NMR {^{1}H}** (126 MHz, $CDCl_3$) δ 173.0(C=O), 145.2(Ar*C*), 138.9(Ar*C*), 136.8(Ar*C*), 136.5(Ar*C*), 136.3(Ar*C*), 136.2(Ar*C*), 135.7(Ar*C*), 134.3(Ar*C*), 129.4(Ar*C*), 129.3(Ar*C*), 129.2(Ar*C*), 128.7(Ar*C*), 128.4(Ar*C*), 127.5(Ar*C*), 127.4(Ar*C*), 127.2(Ar*C*), 123.5(*C*H), 58.7(*CH*), 44.5(*CH*), 21.7(*CH_3*), 21.0(*CH_3*) ppm; **IR ν_{max} (film)**: 1717 cm^{-1} (C=O amide); **HRMS (ESI, m/z)**: calcd. for $C_{31}H_{26}NO_3SNa^+$

550.1214, found 550.1218 (0.70 ppm); **HPLC analysis**: 95:5 e.r., (CHIRALCEL OD-H column, *n*-hexane/*i*-PrOH = 80/20, flow rate = 1 mL/min, λ = 211 nm, 30 °C, t_{major} = 9.1 min, t_{minor} = 17.6 min.

(3*S*,4*S*)-4-(2-methoxyphenyl)-3,6-diphenyl-1-tosyl-3,4-dihydropyridin-2(1*H*)-one (35)

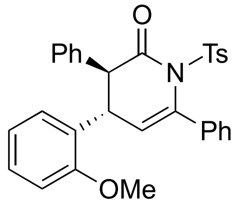

35

Following general procedure **2.6**, α-phenylthioaldehyde **3** (67 mg, 0.3 mmol), NHC precatalyst **30** (14.6 mg, 0.02 mmol), DBU (44.7 µL, 0.3 mmol), corresponding α,β-unsaturated tosyl imine (78 mg , 0.2 mmol) and anhydrous CH_2Cl_2 (2 mL) gave after column chromatography (hexane:EtOAc 90:10) the title compound **35** as a white solid (62.1 mg, 61%); **mp** 86-88 °C; **$[\alpha]^{20}_D$** = +6.2 (c = 0.6 in $CHCl_3$); **^{1}H NMR** (500 MHz, $CDCl_3$) δ 7.84 (d, *J* = 8.0 Hz, 2H, Ar*H*), 7.44 (dd, *J* = 6.7, 2.9 Hz, 2H, Ar*H*), 7.38 – 7.35 (m, 3H, Ar*H*), 7.24 (d, *J* = 7.9 Hz, 2H, Ar*H*), 7.22 – 7.13 (m, 5H, Ar*H*), 7.05 – 6.99 (m, 2H, Ar*H*), 6.86 (t, *J* = 7.4 Hz, 1H, Ar*H*), 6.77 (d, *J* = 8.2 Hz, 1H, Ar*H*), 5.90 (d, *J* = 5.5 Hz, 1H, C*H*), 4.36 (dd, *J* = 7.7, 5.4 Hz, 1H, C*H*), 4.18 (d, *J* = 7.7 Hz, 1H, C*H*), 3.76 (s, 3H, C*H*), 2.44 (s, 3H, CH_3) ppm; **^{13}C NMR {^{1}H}** (126 MHz, $CDCl_3$) δ 173.4(Ar*C*), 157.0(Ar*C*), 144.9(Ar*C*), 140.4(Ar*C*), 137.3(Ar*C*), 137.3(Ar*C*), 136.4(Ar*C*), 129.6(Ar*C*), 129.0(Ar*C*), 128.7(Ar*C*), 128.5(Ar*C*), 128.3(Ar*C*), 128.3(Ar*C*), 128.2(Ar*C*), 127.2(Ar*C*), 127.1(Ar*C*) ,126.0(Ar*C*), 121.5(Ar*C*), 120.6(*CH*), 110.5(Ar*C*), 56.48(*C*H) , 55.26(*C*H) , 39.71(O*C*H_3) , 21.72(*C*H_3) ppm; **HRMS (ESI, m/z)**: calcd. for $C_{31}H_{27}NO_4SNa^+$ 532.1553, found 532.1549 (1.12 ppm); **IR ν_{max} (film)**: 1717 cm^{-1} (C=O amide); **HPLC analysis**: 91:9 e.r., (CHIRALCEL OD-H column, *n*-hexane/*i*-PrOH = 80/20, flow rate = 1 mL/min, λ = 211 nm, 30 °C, t_{major} = 9.1 min, t_{minor} = 11.1min.

(3*S*,4*S*)-4-(4-chlorophenyl)-3,6-diphenyl-1-tosyl-3,4-dihydropyridin-2(1*H*)-one (36)

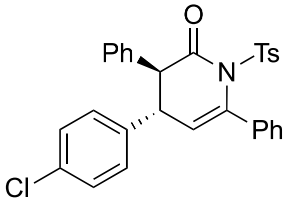

36

Following general procedure **2.6**, α-phenylthioaldehyde **3** (67 mg, 0.3 mmol), NHC precatalyst **30** (14.6 mg, 0.02 mmol), DBU (44.7 µL, 0.3 mmol), corresponding α,β-unsaturated tosyl imine (79 mg , 0.2 mmol) and anhydrous CH_2Cl_2 (2 mL) gave after column chromatography (hexane:EtOAc 90:10) the title compound **36** as a white solid (68 mg, 67%); **mp** 86-90 °C; **$[\alpha]^{20}_D$** = +32.8 (c = 0.9 in $CHCl_3$); **^{1}H NMR** (500 MHz, $CDCl_3$) δ 7.82 (d, *J* = 8.0 Hz, 2H, Ar*H*), 7.38 (tt, *J* = 6.5, 3.9 Hz, 5H, Ar*H*), 7.28 (d, 2H, Ar*H*), 7.18 – 7.11 (m, 5H, Ar*H*), 6.98 (d, *J* = 8.1 Hz, 2H, Ar*H*), 6.82 (dd, *J* = 6.7, 3.0 Hz, 2H, Ar*H*), 5.95 (d, *J* = 4.2 Hz, 1H, CH), 4.03 (dd, *J* = 10.6, 4.3 Hz, 1H,CH), 3.86 (d, *J* = 10.5 Hz, 1H, CH), 2.46 (s, 3H, CH_3) ppm.;**^{13}C NMR {^{1}H}** (126 MHz, $CDCl_3$) δ 172.8(C=O), 145.2(ArC), 140.5(ArC), 138.4(ArC), 136.9(ArC), 136.2(ArC), 136.1(ArC), 132.9(ArC), 129.5(ArC), 129.1(ArC), 128.8(ArC), 128.7(ArC), 128.5(ArC), 128.5(ArC), 127.5(ArC), 126.1(ArC), 122.1(CH), 58.8(CH), 44.5(CH), 21.7(CH3) ppm. **IR ν_{max} (film)**: 1718 cm^{-1} (C=O amide); **HRMS (ESI, m/z)**: calcd. for $C_{30}H_{24}ClNO_3SH^+$ 514.1239, found 514.1236 (-0.43 ppm); **HPLC analysis**: 95:5 e.r., (CHIRALCEL OD-H column, *n*-hexane/*i*-PrOH = 80/20, flow rate = 1 mL/min, λ = 211 nm, 30 °C t_{major} = 12.1 min, t_{minor} = 19.1 min).

(3*S*,4*S*)-3-phenyl-4,6-di-*p*-tolyl-1-tosyl-3,4-dihydropyridin-2(1*H*)-one (37)

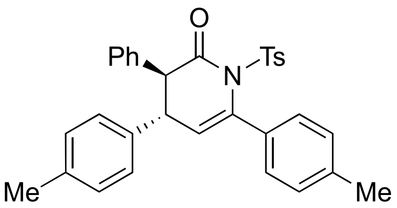

37

Following general procedure **2.6**, α-phenylthioaldehyde **3** (67 mg, 0.3 mmol), NHC precatalyst **30**(14.6 mg, 0.02 mmol), DBU (44.7 µL, 0.3 mmol), corresponding α,β-unsaturated tosyl imine (78 mg , 0.2 mmol) and anhydrous CH_2Cl_2 (2 mL) gave after column chromatography (hexane:EtOAc 90:10) the title compound **37** as a white solid (79.5 mg, 77%); **mp** 76-78°C; **$[\alpha]^{20}_D$** = +47.4 (c = 0.8 in $CHCl_3$); **^{1}H NMR** (500 MHz, $CDCl_3$) δ 7.87 (d, *J* = 8.4 Hz, 2H, Ar*H*), 7.34 (d, *J* = 8.2 Hz, 2H, Ar*H*), 7.31 – 7.27 (m, 2H, Ar*H*), 7.20 (d, *J* = 7.8 Hz, 2H, Ar*H*), 7.17 – 7.09 (m, 3H, Ar*H*), 6.98 (d, *J* = 7.9 Hz, 2H, Ar*H*), 6.93 (d, *J* = 8.2 Hz, 2H, Ar*H*), 6.83 – 6.77 (m, 2H, Ar*H*), 5.97 (d, *J* = 4.3 Hz, 1H, C*H*), 3.99 (dd, *J* = 10.6, 4.3 Hz,

1H, C*H*), 3.89 (d, *J* = 10.5 Hz, 1H, C*H*), 2.47 (s, 3H, CH_3), 2.41 (s, 3H, CH_3), 2.25 (s, 3H, CH_3) ppm.; **^{13}C NMR {^{1}H}** (126 MHz, $CDCl_3$) δ 173.2(C=O), 144.9(Ar*C*), 139.9(Ar*C*), 138.4(Ar*C*), 136.9(Ar*C*), 136.7(Ar*C*), 136.5(Ar*C*), 136.4(Ar*C*), 134.4(Ar*C*), 129.5(Ar*C*), 129.3(Ar*C*), 129.1(Ar*C*), 129.0(Ar*C*), 128.7(Ar*C*), 128.3(Ar*C*), 127.6(Ar*C*), 127.3(Ar*C*), 125.8(Ar*C*), 122.4(CH), 58.9(CH), 44.6(CH), 21.7(CH_3), 21.3(CH_3), 21.0(CH_3) ppm; **IR ν_{max} (film)**: 1715 cm^{-1} (C=O amide); **HRMS(ESI, m/z)**: calcd. for $C_{32}H_{29}NO_3SNa^+$ 530.1760, found 530.1761(0.12 ppm); **HPLC analysis**: 93:7 e.r., (CHIRALCEL OD-H column, *n*-hexane/*i*-PrOH = 80/20, flow rate = 1 mL/min, λ = 211 nm, 30 °C, t_{major} = 9.0 min, t_{minor} = 12.8 min)

(3*R*,4*S*)-3-ethyl-4,6-diphenyl-1-tosyl-3,4-dihydropyridin-2(1*H*)-one (38)

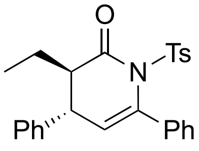

38

Following general procedure **2.6**, a-phenylthioaldehyde **3** (67 mg, 0.3 mmol), NHC precatalyst **30**(14.6 mg, 0.02 mmol), DBU (44.7 µL, 0.3 mmol), corresponding a,b-unsaturated tosyl imine (72.3 mg , 0.2 mmol) and anhydrous CH_2Cl_2 (2 mL) gave after column chromatography (hexane:EtOAc 90:10) the title compound **38** as a colourless liquid (47 mg, 54%); **$[\alpha]^{20}_D$** = +20.0 (c = 0.21 in $CHCl_3$); **^{1}H NMR** (500 MHz, $CDCl_3$) δ 7.83 – 7.77 (m, 2H, Ar-*H*), 7.41 (qd, *J* = 4.1, 1.8 Hz, 2H, Ar-*H*), 7.38 – 7.28 (m, 6H, Ar-*H*), 7.26 – 7.13 (m, 4H, Ar-*H*), 5.87 (d, *J* = 4.4 Hz, 1H, =C*H*), 3.64 (dd, *J* = 10.4, 4.4 Hz, 1H, Ar-C*H*), 2.74 (ddd, J = 10.4, 6.2, 4.2 Hz, 1H, C(O)-C*H*). 2.43 (s, 3H, Ar-CH_3), 1.71-1.61(m, 1H, CH_2), 1.36 (ddd, *J* = 13.8, 7.4, 6.3 Hz, 1H, CH_2), 0.71 (t, *J* = 7.4 Hz, 3H, CH_3).; **^{13}C NMR** (126 MHz, $CDCl_3$) δ 173.8(*C*=O), 144.9 (Ar-*C*), 140.4(Ar-*C*), 139.4(Ar-*C*), 137.3(Ar-*C*), 136.6(Ar-*C*), 129.2(Ar-*C*), 129.1(Ar-*C*), 129.0(Ar-*C*), 128.4(Ar-*C*), 128.4(Ar-*C*) 128.3(Ar-*C*), 127.7(Ar-*C*), 127.3(Ar-*C*), 125.8(Ar-*C*), 123.2(=*CH*), 51.9 (*C*H), 40.9(*C*H), 21.7(*C*H_2), 20.8(*C*H_3), 10.0(*C*H_3).ppm; **IRν_{max}(film)**: 1712(C=O amide), 1598, 1420, 1438, 1327, 1199 cm^{-1} ;**HRMS(ESI, m/z)**: calcd. for $C_{26}H_{25}NO_3SNa^+$ 454.1443, found 454.1447 (-0.96 ppm); **HPLC analysis**: 99:1 e.r. (major diastereomer), (CHIRALCEL AD-H column, *n*-hexane/*i*-PrOH = 90/10, flow rate = 0.5 mL/min, λ = 211 nm, 30 °C, t_{major} = 38.4 min, t_{minor} = 32.0 min); 95:5 e.r. (minor diastereomer),

(CHIRALCEL AD-H column, *n*-hexane/*i*-PrOH = 90/10, flow rate = 0.5 mL/min, λ = 211 nm, 30 °C, t_{major} = 28.2 min, t_{minor} = 36.1 min)

(3*R*,4*S*)-4-(4-chlorophenyl)-3-ethyl-6-phenyl-1-tosyl-3,4-dihydropyridin-2(1*H*)-one (39)

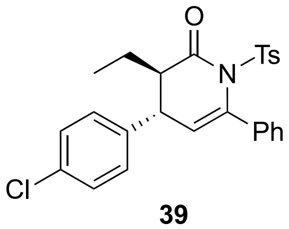

39

Following general procedure **2.6**, a-phenylthioaldehyde **3** (67 mg, 0.3 mmol), NHC precatalyst **30**(14.6 mg, 0.02 mmol), DBU (44.7 µL, 0.3 mmol), corresponding a,b-unsaturated tosyl imine (79.2 mg , 0.2 mmol) and anhydrous CH_2Cl_2 (2 mL) gave after column chromatography (hexane:EtOAc 90:10) the title compound **39** as a white solid (46 mg, 49%); **mp** 60-64°C; **$[\alpha]^{20}_D$** = +38.5 (c = 0.3 in $CHCl_3$); **^{1}H NMR** (500 MHz, $CDCl_3$) δ 7.76 (d, *J* = 8.4 Hz, 2H, Ar-*H*), 7.43 – 7.39 (m, 2H, Ar-*H*), 7.39 – 7.35 (m, 4H, Ar-*H*), 7.24 (dd, *J* = 8.4, 2.3 Hz, 3H, Ar-*H*), 7.16 – 7.12 (m, 2H, Ar-*H*), 5.83 (d, *J* = 4.8 Hz, 1H, =C*H*), 3.62 (dd, *J* = 9.5, 4.8 Hz, 1H, Ar-C*H*), 2.71 (ddd, *J* = 9.4, 6.3, 4.6 Hz, 1H, C(O)-C*H*), 2.43 (s, 3H, Ar-CH_3), 1.61-1.71 (m, 1H, CH_2), 1.40 (dt, *J* = 14.0, 7.0 Hz, 1H, CH_2), 0.75 (t, *J* = 7.4 Hz, 3H, CH_3) ppm **^{13}C NMR** (126 MHz, $CDCl_3$) δ 173.4 (*C*=O), 145.0(Ar-*C*), 139.9 (Ar-*C*), 138.9(Ar-*C*), 137.2(Ar-*C*), 136.4(Ar-*C*), 133.1(Ar-*C*), 129.6(Ar-*C*), 129.5(Ar-*C*), 129.4(Ar-*C*), 129.3(Ar-*C*), 129.2(Ar-*C*), 129.1(Ar-*C*), 129.0(Ar-*C*), 128.5(Ar-*C*), 128.4(Ar-*C*), 125.8(Ar-*C*), 122.0(=*CH*), 51.8(*C*H), 40.2(*C*H), 21.7(*C*H_2), 21.0(*C*H3), 10.1(*C*H3) ppm; **IRν_{max}(film)**: 1718(C=O amide), 1597, 1420, 1491, 1366, 1087 cm^{-1};**HRMS(ESI, m/z)**: calcd. for $C_{26}H_{24}ClNO_3SH^+$ 466.1238, found 466.1242 (0.82 ppm); **HPLC analysis**: 95:5 e.r. (major diastereomer), (CHIRALCEL ID column, *n*-hexane/*i*-PrOH = 90/10, flow rate = 1 mL/min, λ = 254 nm, 30 °C, t_{major} = 29.3 min, t_{minor} = 41.1 min); 96:4 e.r. (minor diastereomer), (CHIRALCEL AD-H column, *n*-hexane/*i*-PrOH = 90/10, flow rate = 0.5 mL/min, λ = 254 nm, 30 °C, t_{major} = 38.6 min, t_{minor} = 32.4 min)

4. Crystallography Details

Using a purified sample of dihydropyridinone **29**, single crystals were obtained from concentrated solutions of **29** in EtOAc, to authenticate the absolute stereochemistry of the major diastereomer. Over a period of 8 weeks single crystals suitable for SCXRD were obtained. The solid-state analysis revealed that the crystals did not represent the title compound **29** but rather **31** was obtained (Figure 2). The SCXRD structure shows that *N-C* tosyl migration had occurred. Crystal data was solved by ShelXT direct methods or intrinsic phasing and refined using a full-matrix least square refinement on $|F|^2$ using ShelXL.1-3 in Olex.[27]

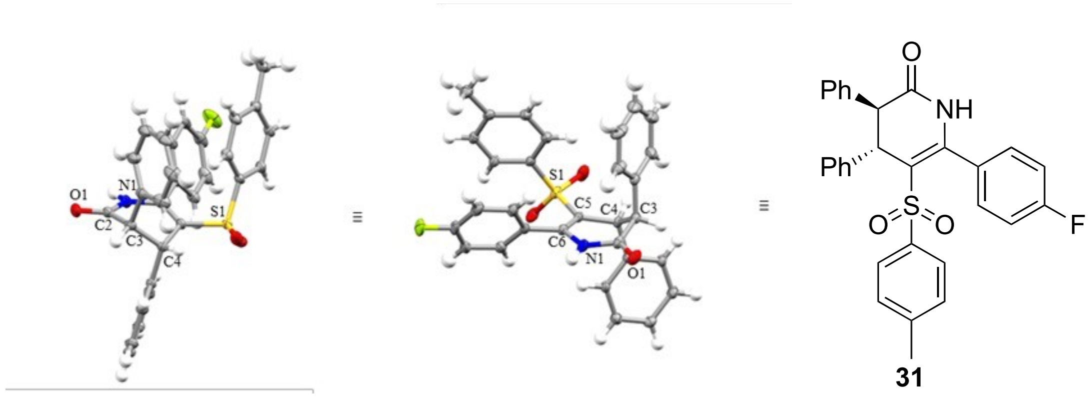

Figure 2: Molecular structure of **29**, all atoms shown at 50% ellipsoid probability, absolute stereochemistry determined for the lactam. Anti- diastereomer and absolute stereochemistry of (3*S*,4*S*) is assigned.

**Crystal data for Compound 31**	
Local Code	PE_JL22002
Chemical formula	$C_{30}H_{24}FNO_3S$
M_r	497.56
CCDC	**2310270**
Crystal system, space group	Orthorhombic, $P2_12_12_1$
Temperature (K)	120
Α, β, γ (°)	90, 90, 90
a, *b*, *c* (Å)	7.4071 (1), 15.8960 (1), 21.0437 (2)
V (Å^3)	2477.75 (4)
Z	4
Radiation type	Cu *K*α
μ (mm^{-1})	1.50
Crystal size (mm)	0.17 × 0.12 × 0.09
**Data collection**	
Diffractometer	SuperNova, Dual, Cu at home/near, Atlas
Absorption correction	Multi-scan<br>*CrysAlis PRO* 1.171.41.99a (Rigaku Oxford Diffraction, 2021) Empirical absorption correction using spherical harmonics, implemented in SCALE3 ABSPACK scaling algorithm.
T_{min}, T_{max}	0.677, 1.000

No. of measured, independent and observed [$I > 2\sigma(I)$] reflections	26615, 5159, 4987
$(\sin \theta/\lambda)_{max}$ (Å^{-1})	0.629
**Refinement**	
$R[F^2 > 2\sigma(F^2)]$, $wR(F^2)$, S	0.045, 0.118, 1.05
No. of reflections	5159
No. of parameters	326
H-atom treatment	H-atom parameters constrained
$\Delta\rho_{max}$, $\Delta\rho_{min}$ (e Å^{-3})	0.30, −0.50
Absolute structure	Flack x determined using 2079 quotients [(I+)-(I-)]/[(I+)+(I-)] (Parsons, Flack and Wagner, Acta Cryst. B69 (2013) 249-259).
Absolute structure parameter	−0.010 (9)

5. References

[1] aM. S. Kerr, J. Read de Alaniz, T. Rovis, *J. Org. Chem.,* **2005**, *70*, 5725-5728; bJ. R. de Alaniz, M. S. Kerr, J. L. Moore, T. Rovis, *J. Org. Chem.,* **2008**, *73*, 2033-2040; cJ. Mahatthananchai, J. W. Bode, *Chem. Sci.* **2012**, *3*, 192-197; dR. S. Massey, C. J. Collett, A. G. Lindsay, A. D. Smith, A. C. O'Donoghue, *J. Am. Chem. Soc.,* **2012**, *134*, 20421-20432.

[2] T. Sato, J. Otera, H. Nozaki, *J. Org. Chem.,* **1990**, *55*, 6116-6121.

[3] D. Craig, N. P. King, A. N. Shaw, *Tetrahedron Lett.,* **1997**, *38*, 8599-8602.

[4] V. H. Rawal, M. Akiba, M. P. Cava, *Synth. Commun.* **1984**, *14*, 1129-1139.

[5] B. J. M. Jansen, R. M. Peperzak, A. de Groot, *Recueil des Travaux Chimiques des Pays-Bas* **1987**, *106*, 489-494.

[6] T. Sato, M. Inoue, S. Kobara, J. Otera, H. Nozaki, *Tetrahedron Letters* **1989**, *30*, 91-94.

[7] B. Jansen, R. Peperzak, A. de Groot, *Recueil des Travaux Chimiques des Pays-Bas* **1987**, *106*, 489-494.

[8] L. A. Paquette, T. M. Mitzel, M. B. Isaac, C. F. Crasto, W. W. Schomer, *The J. Org. Chem.,* **1997**, *62*, 4293-4301.

[9] F. S. Tjoeng, G. A. Heavner, *J. Org. Chem.,* **1983**, *48*, 355-359.

[10] Z. Kong, C. Pan, M. Li, L. Wen, W. Guo, *Green Chemistry* **2021**, *23*, 2773-2777.

[11] A. Ozanne-Beaudenon, S. Quideau, *Tetrahedron Lett.* **2006**, *47*, 5869-5873.

[12] S. Biswas, R. A. Watile, J. S. M. Samec, *Chem. – Eur. J* **2014**, *20*, 2159-2163.

[13] K. Chibale, S. Warren, *J.Chem. Soc., Perkin Transactions 1* **1996**, 1935-1940.

[14] S.-F. Zhou, X.-Q. Pan, Z.-H. Zhou, A. Shoberu, P.-Z. Zhang, J.-P. Zou, *J. Org. Chem.,* **2015**, *80*, 5348-5354.

[15] L. Capella, P. C. Montevecchi, D. Nanni, *J. Org. Chem.,* **1994**, *59*, 7379-7382.

[16] L. Sancineto, C. Tidei, L. Bagnoli, F. Marini, V. Lippolis, M. Arca, E. J. Lenardão, C. Santi, *Eur. J. Org. Chem.,* **2016**, *2016*, 2999-3005.

[17] L. Wang, J. Qiao, J. Wei, Z. Liang, X. Xu, N. Li, *Tetrahedron* **2020**, *76*, 130750.

[18] W. Dan, H. Deng, J. Chen, M. Liu, J. Ding, H. Wu, *Tetrahedron* **2010**, *66*, 7384-7388.

[19] C. Simion, I. Hashimoto, Y. Mitoma, A. M. Simion, N. Egashira, *Phosphorus, Sulfur, and Silicon and the Related Elements* **2010**, *185*, 2480-2488.

[20] T. Böttcher, S. A. Sieber, *Angew. Chem. Int. Ed.,* **2008**, *47*, 4600-4603.

[21] X. Zheng, W. Fu, J. Xiong, J. Xi, X. Ni, T. Tang, *Catalysis Today* **2016**, *264*, 152-157.

[22] B. Chen, X.-F. Wu, *Org. Biomol. Chem.,* **2021**, *19*, 9654-9658.

[23] M. Meurillon, L. Chaloin, C. Périgaud, S. Peyrottes, *Eur. J. Org. Chem.* **2011**, *2011*, 3794-3802.

[24] I. Dhimitruka, J. SantaLucia, *Org. Lett.,* **2006**, *8*, 47-50.
[25] Y.-R. Li, Z.-N. Xu, B. Bai, Z.-Q. Wang, G.-C. Guo, *Chin. J. Chem.* **2019**, *37*, 769-774.
[26] V. A. Ignatenko, N. Deligonul, R. Viswanathan, *Org. Lett.* **2010**, *12*, 3594-3597.

[27] Sheldrick, G., SHELXT -Integrated space-group and crystal-structure determination. *Acta Cryst. A*., **2015**, 71(1), 3-8; Sheldrick, G., Crystal structure refinement with SHELXL. *Acta Cryst. C*., **2015**, 71(1), 3-8.

1. NMR Spectra

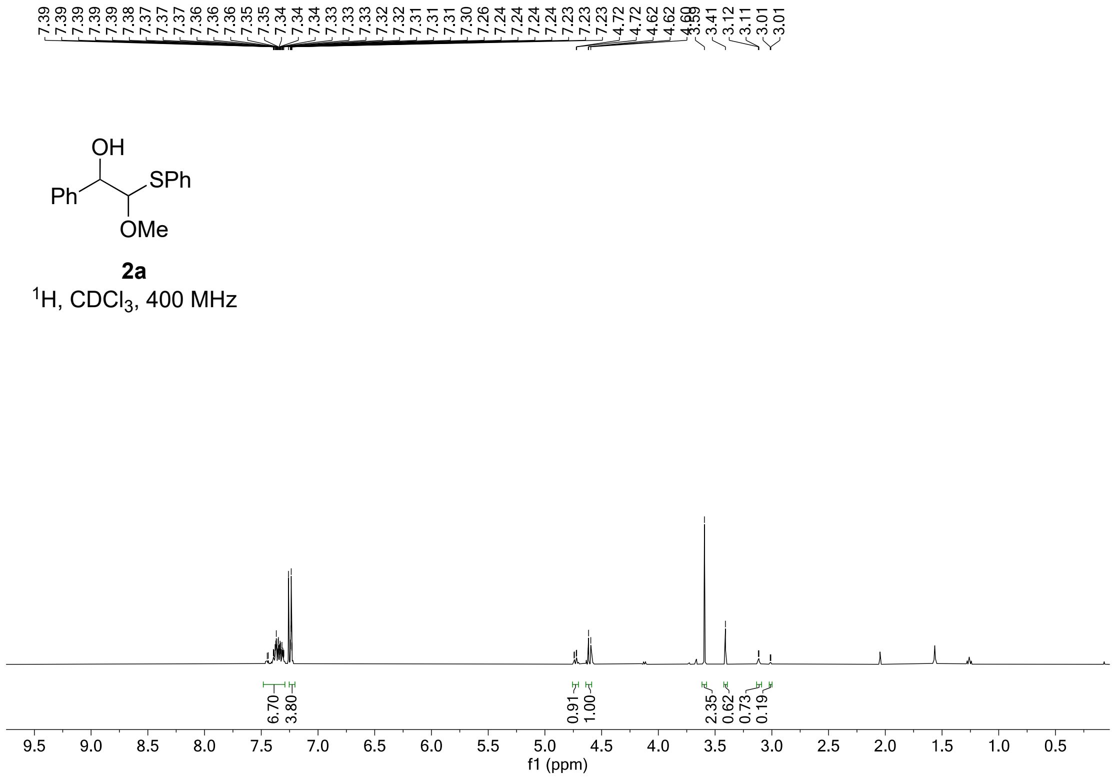

2a
^{1}H, $CDCl_3$, 400 MHz

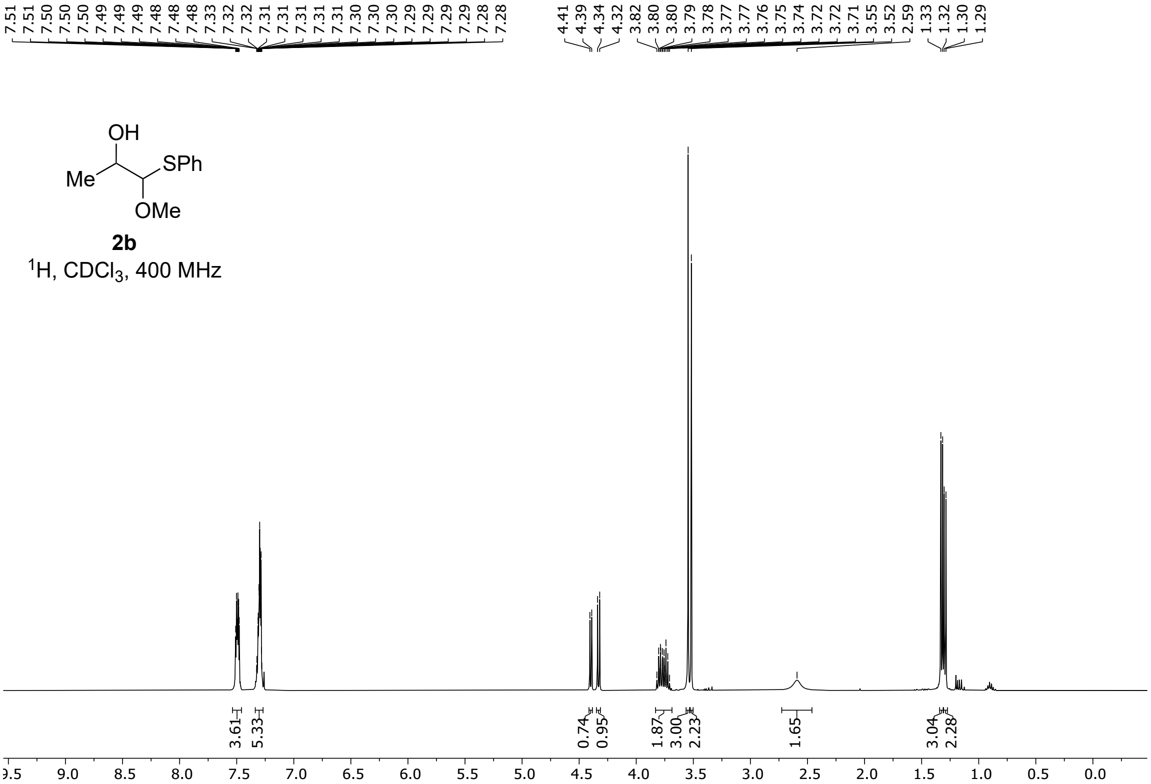

2b
^{1}H, $CDCl_3$, 400 MHz

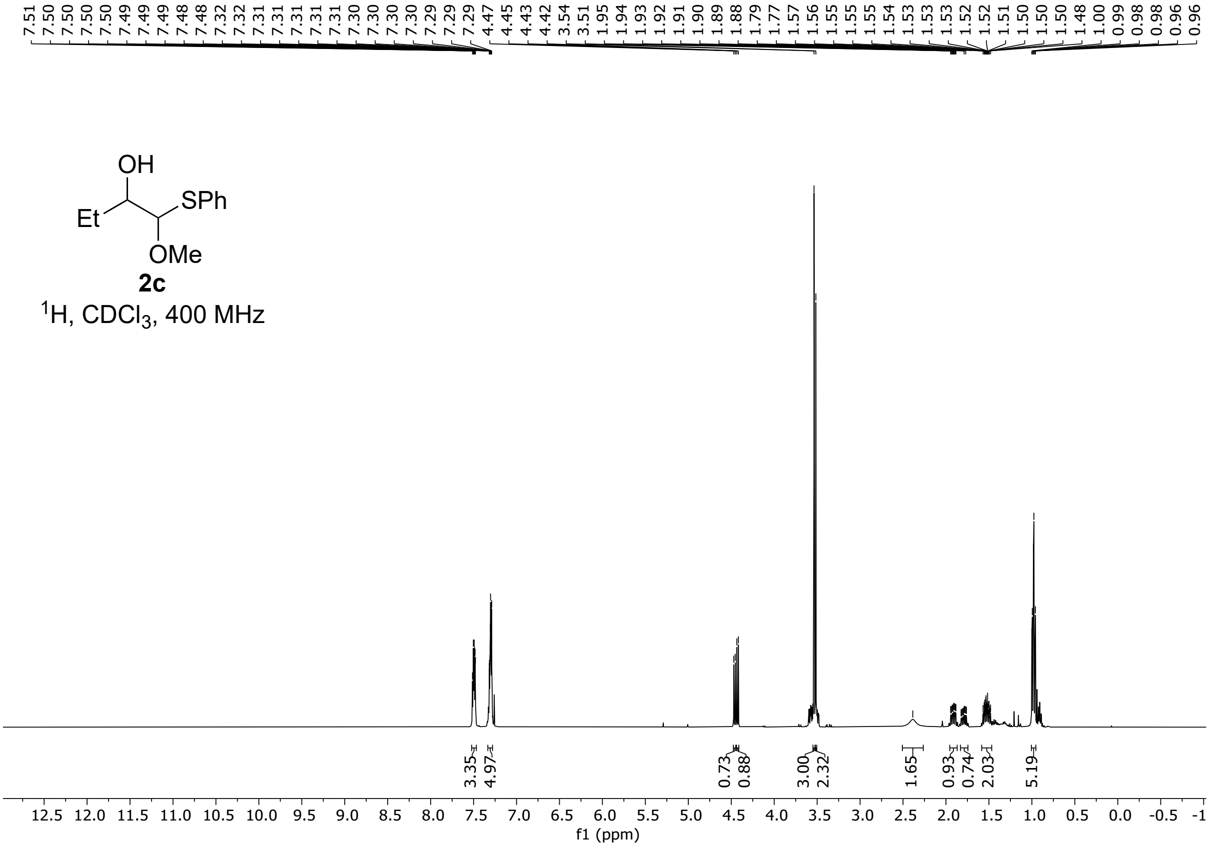

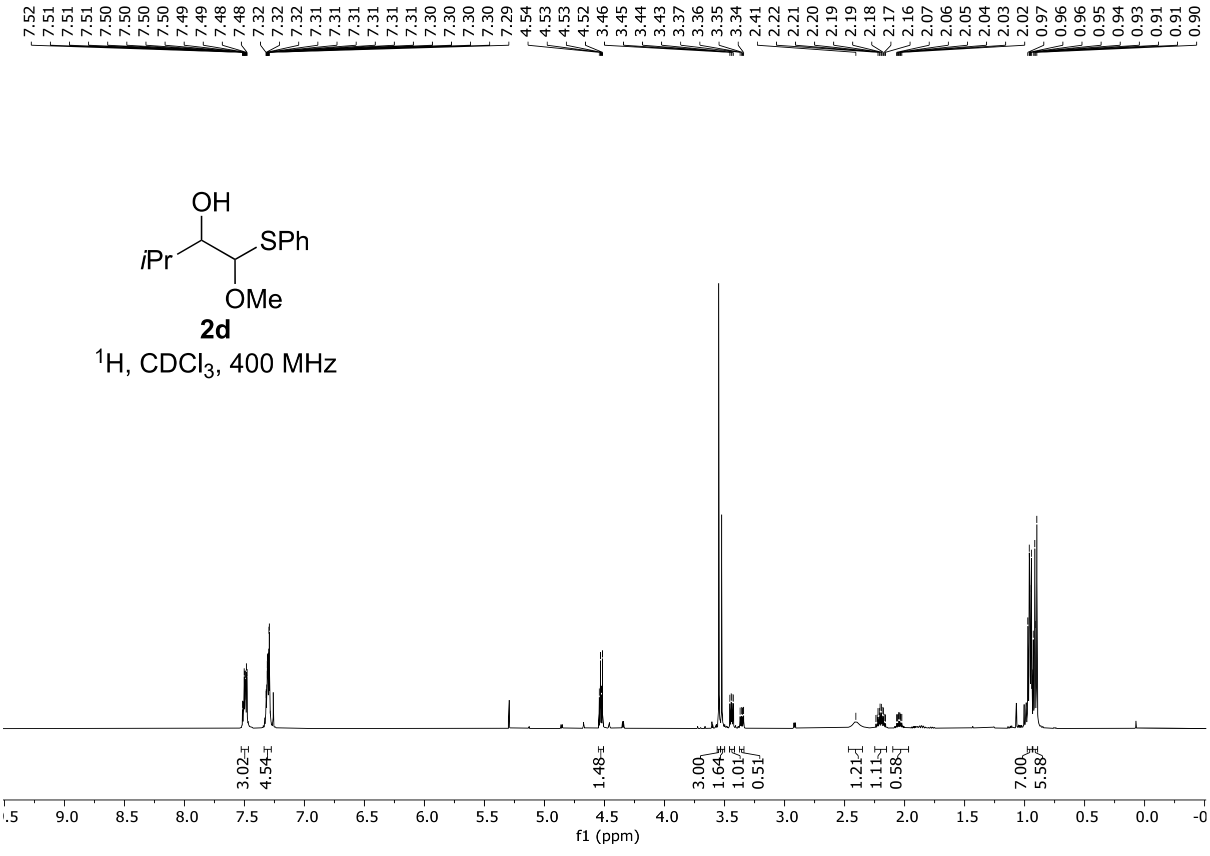

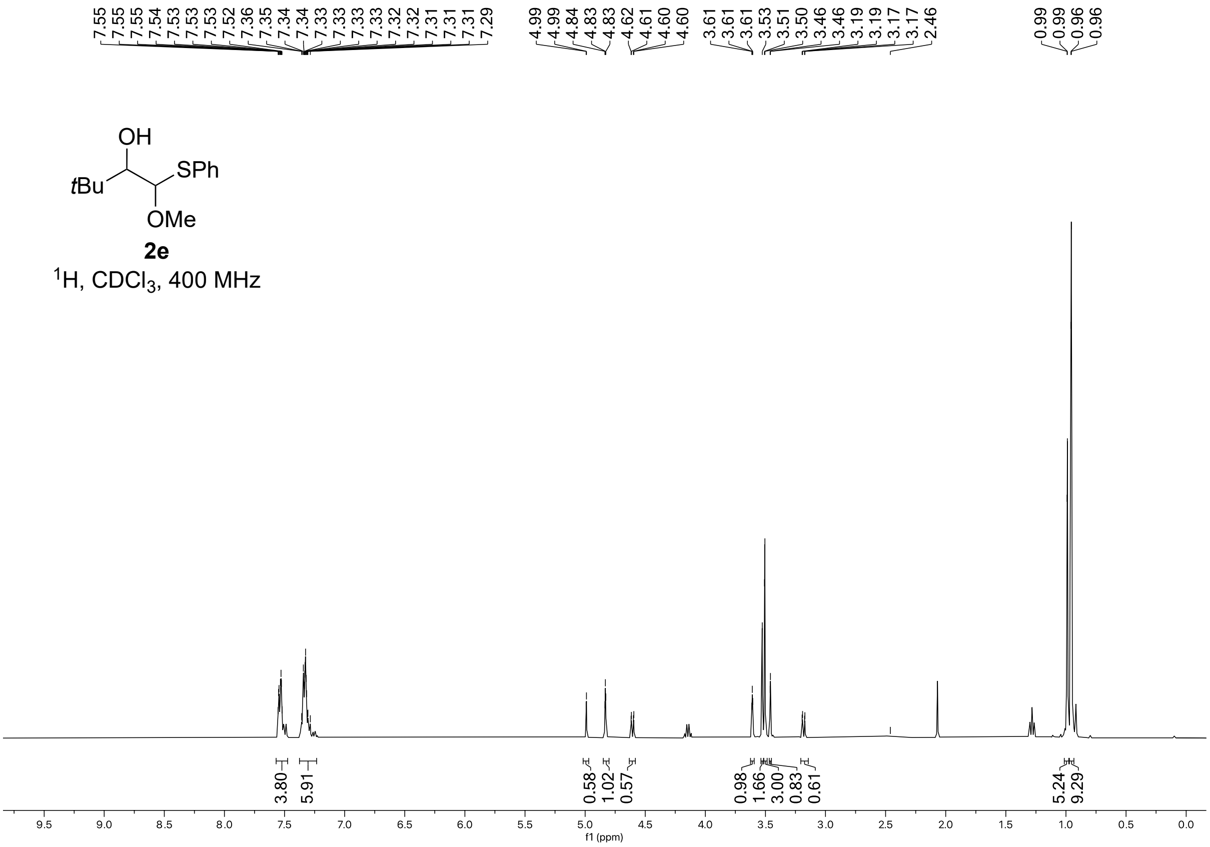

OH
SPh
tBu
OMe
2e
1H, CDCl3, 400 MHz
f1 (ppm)

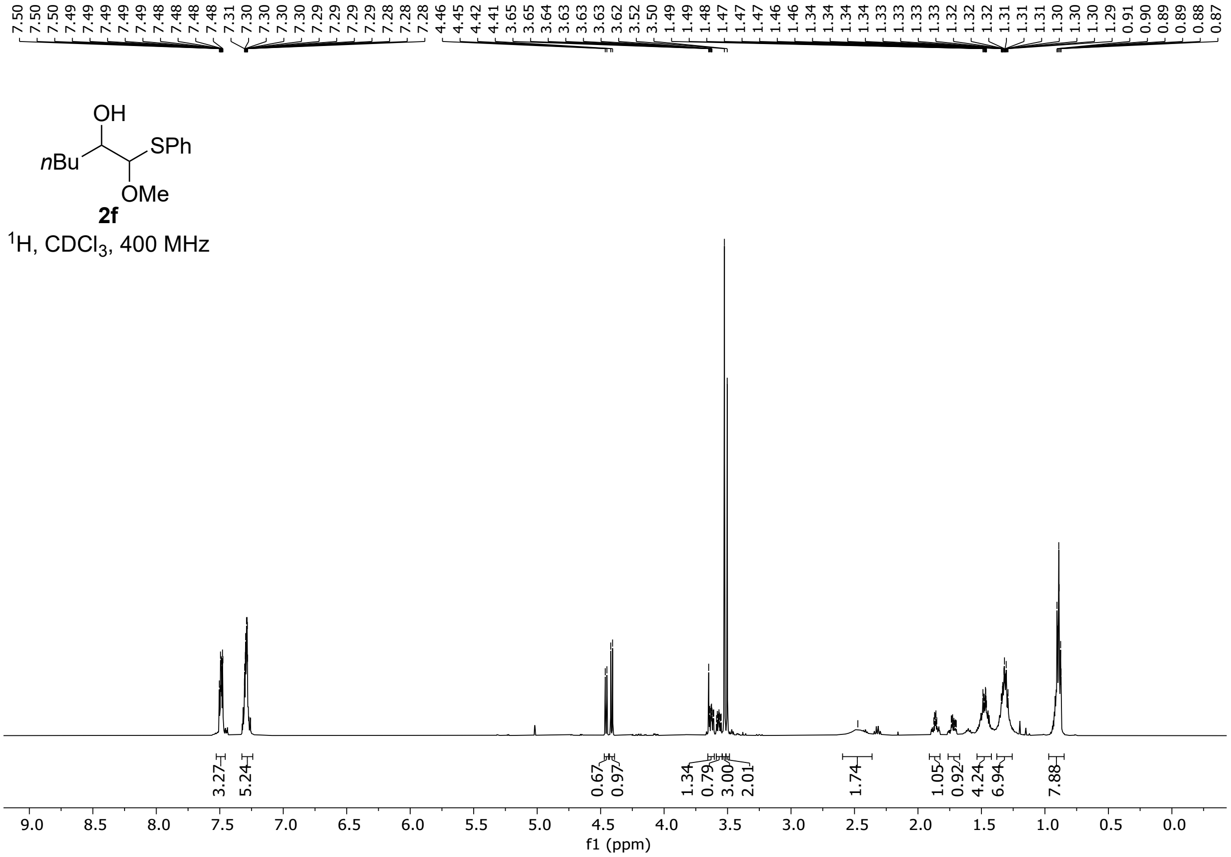

OH
SPh
nBu
OMe
2f
1H, CDCl3, 400 MHz
f1 (ppm)

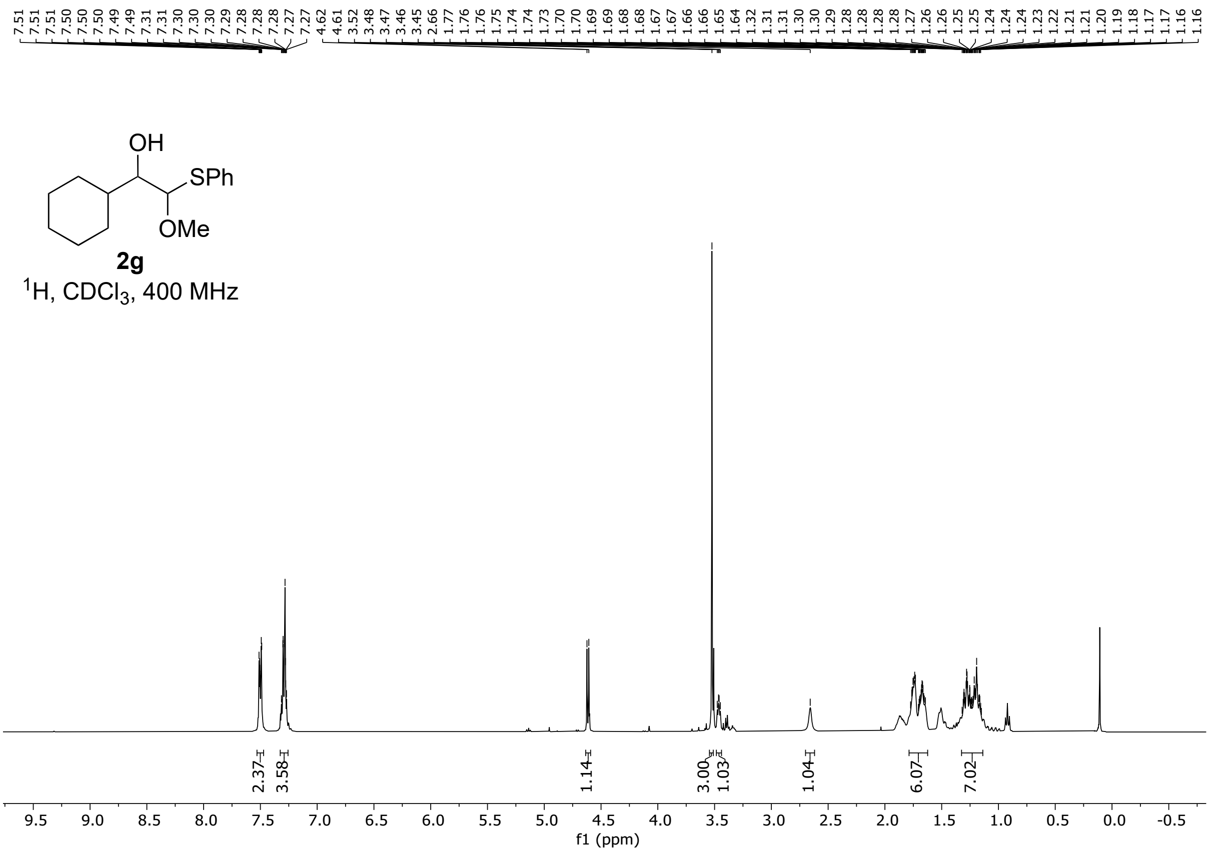
OH
SPh
OMe
2g
1H, CDCl3, 400 MHz
2.37
3.58
1.14
3.00
1.03
1.04
6.07
7.02
f1 (ppm)

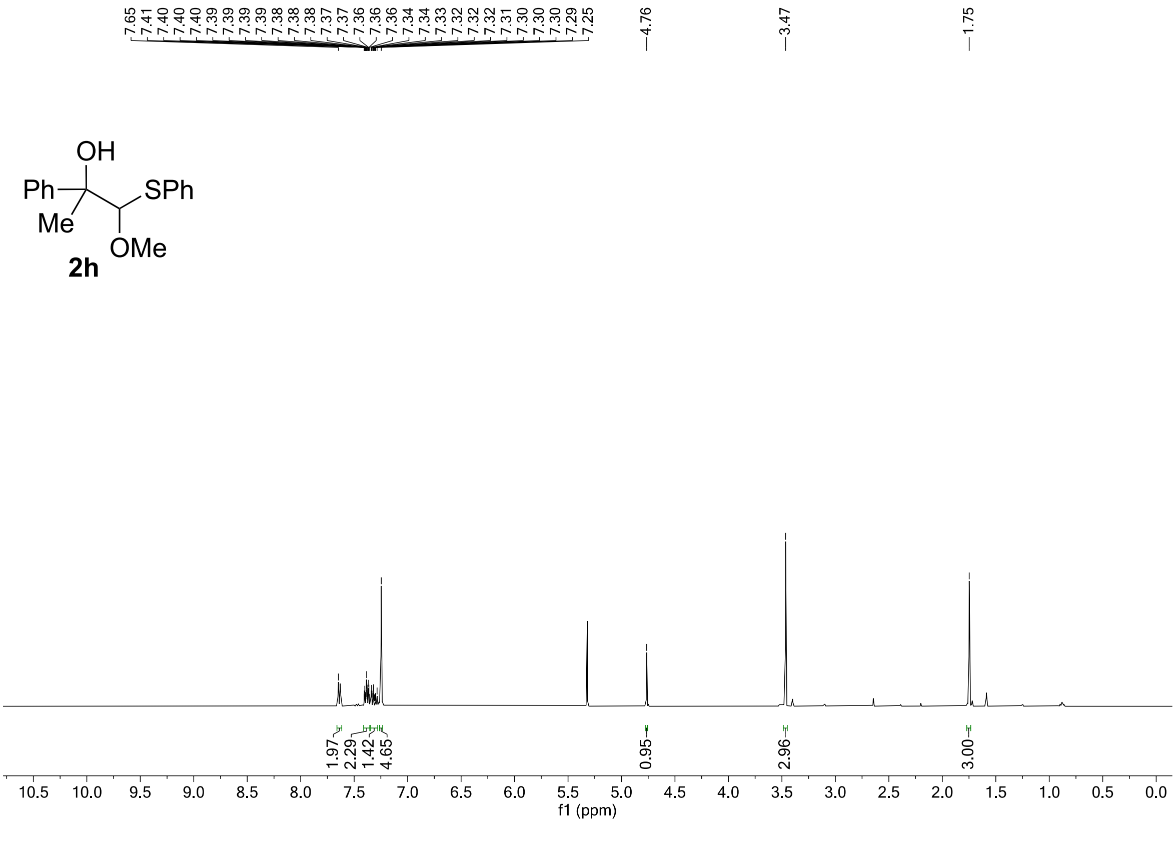
OH
Ph
SPh
Me
OMe
2h
7.65
4.76
3.47
1.75
1.97
2.29
1.42
4.65
0.95
2.96
3.00
f1 (ppm)

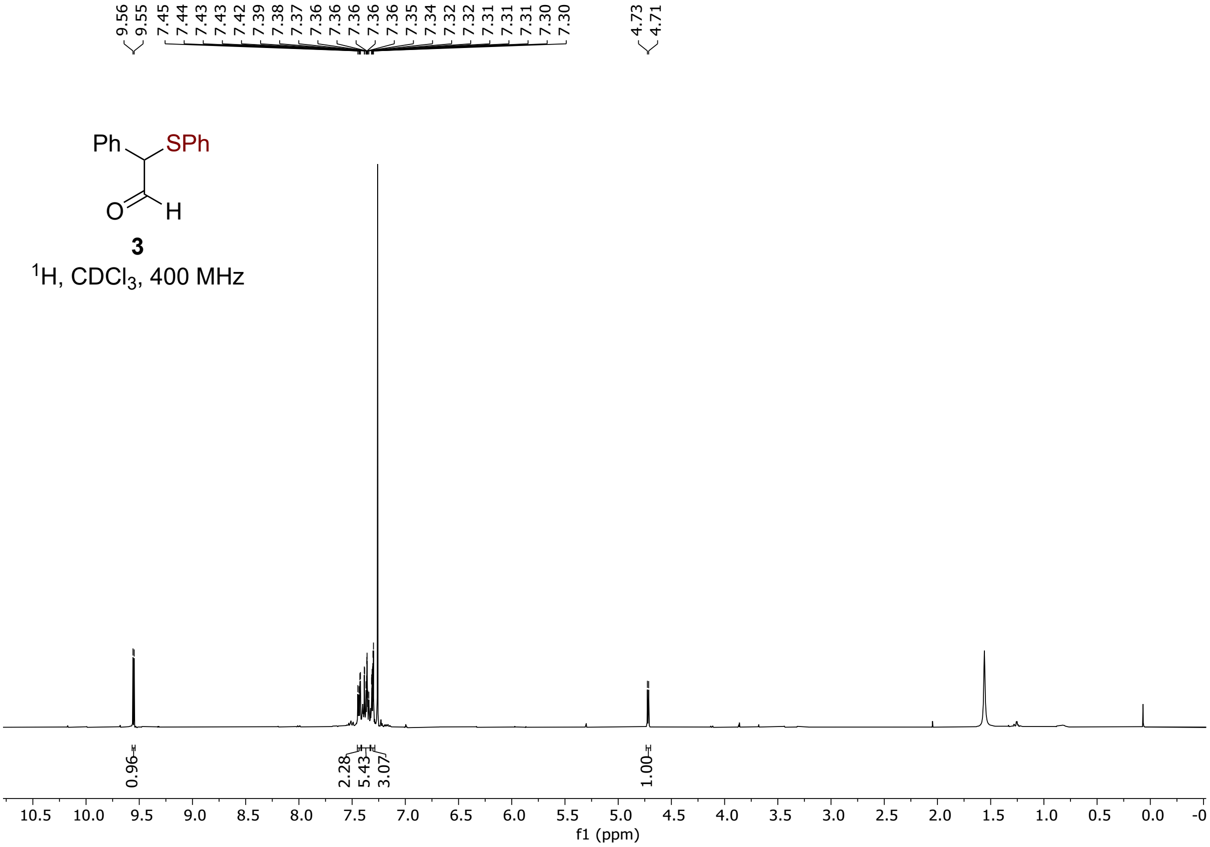
Ph
SPh
O
H
3
^{1}H, $CDCl_3$, 400 MHz
f1 (ppm)

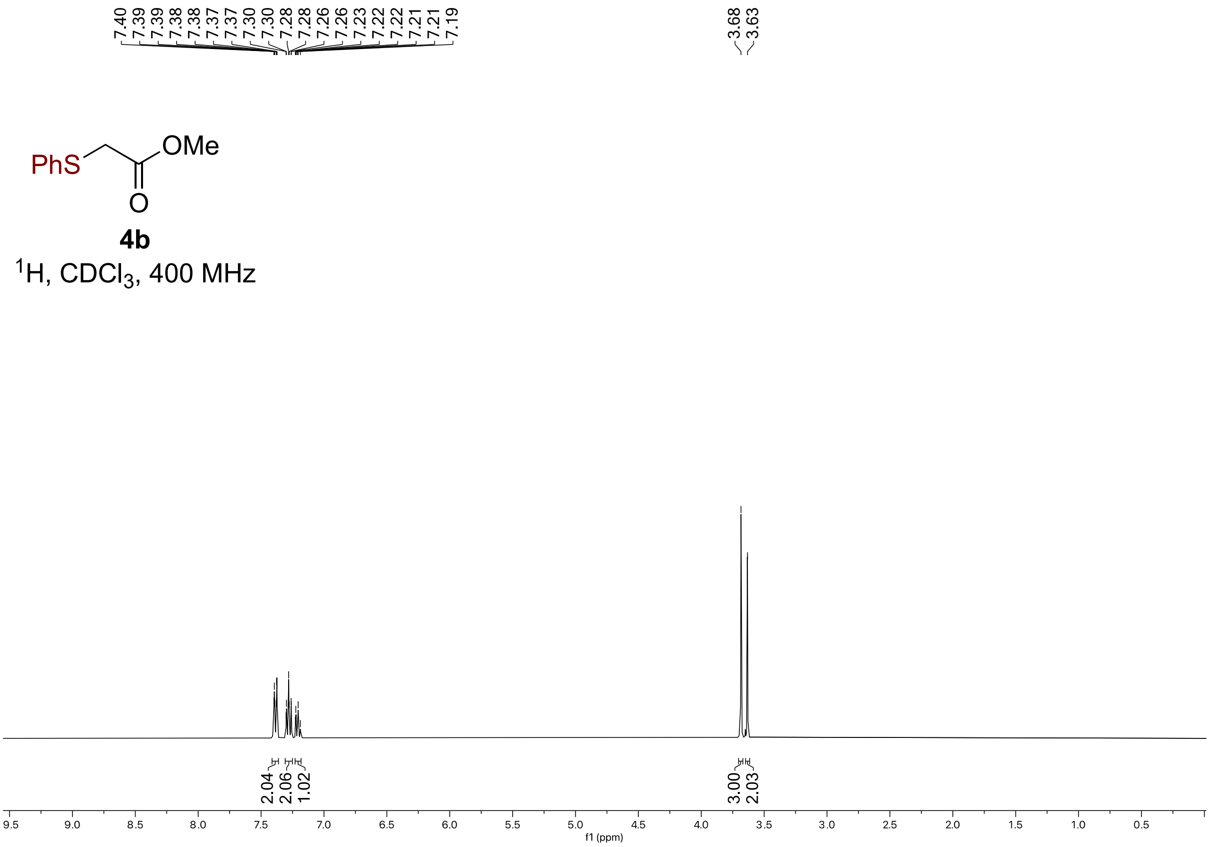
PhS
OMe
O
4b
^{1}H, $CDCl_3$, 400 MHz
f1 (ppm)

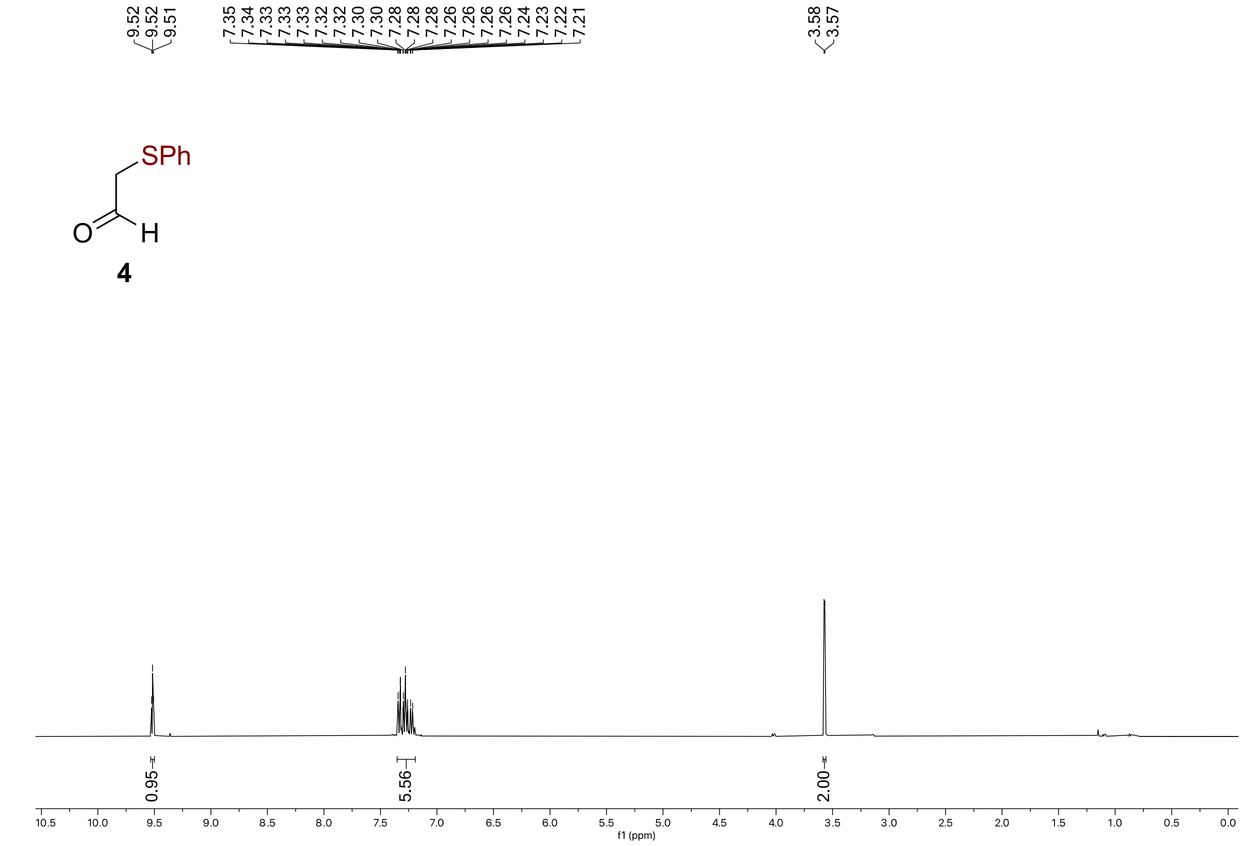
9.52
9.52
9.51
7.35
7.34
7.33
7.33
7.33
7.32
7.32
7.30
7.30
7.28
7.28
7.28
7.26
7.26
7.26
7.24
7.23
7.22
7.21
3.58
3.57
SPh
O
H
4
0.95
5.56
2.00
10.5 10.0 9.5 9.0 8.5 8.0 7.5 7.0 6.5 6.0 5.5 5.0 4.5 4.0 3.5 3.0 2.5 2.0 1.5 1.0 0.5 0.0
f1 (ppm)

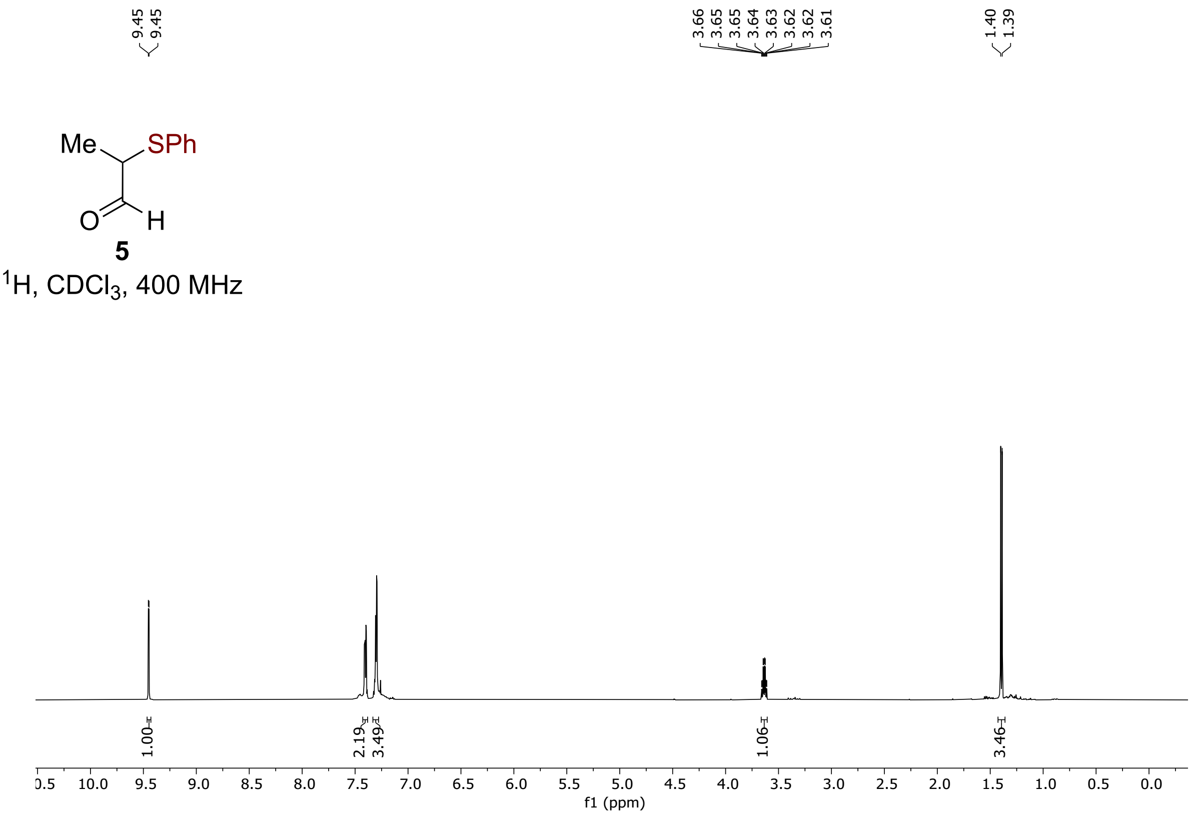
9.45
9.45
3.66
3.65
3.65
3.64
3.63
3.62
3.62
3.61
1.40
1.39
Me
SPh
O
H
5
1H, CDCl3, 400 MHz
1.00
2.19
3.49
1.06
3.46
10.0 9.5 9.0 8.5 8.0 7.5 7.0 6.5 6.0 5.5 5.0 4.5 4.0 3.5 3.0 2.5 2.0 1.5 1.0 0.5 0.0
f1 (ppm)

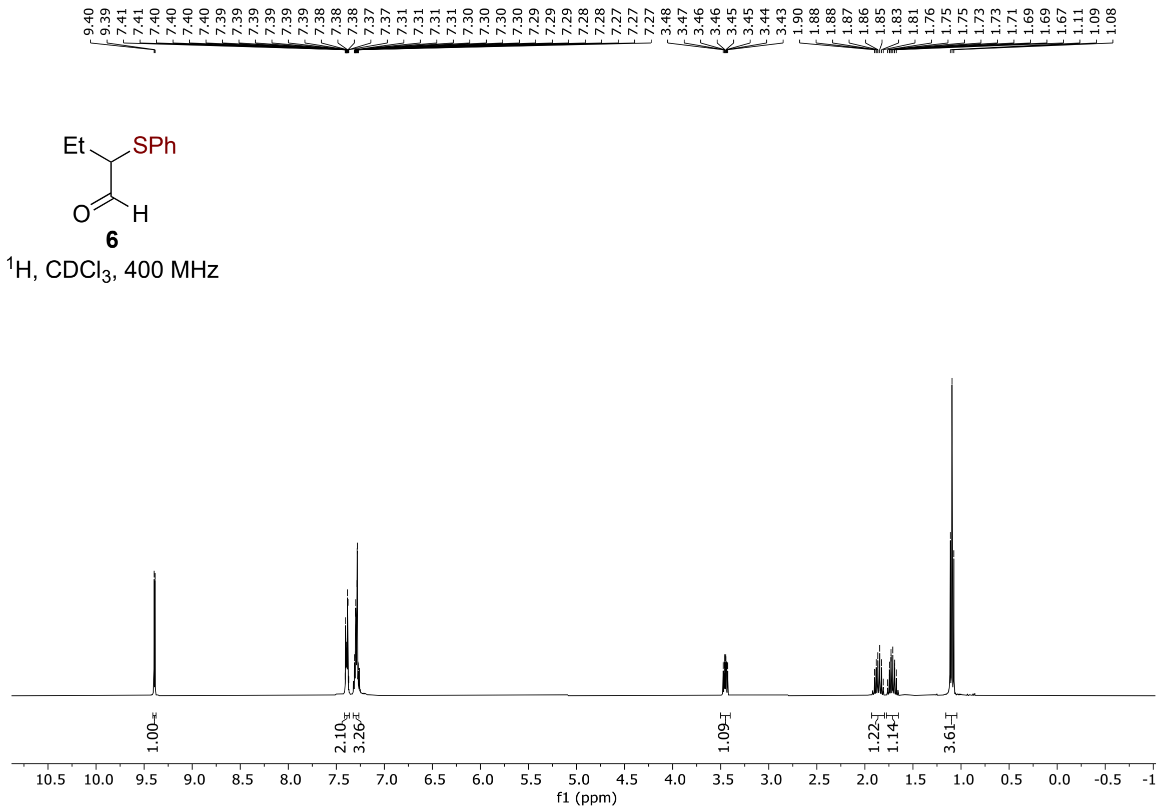
9.40 9.39 7.41 7.41 7.40 7.40 7.40 7.40 7.39 7.39 7.39 7.39 7.39 7.39 7.38 7.38 7.38 7.37 7.37 7.31 7.31 7.31 7.31 7.30 7.30 7.30 7.30 7.29 7.29 7.29 7.28 7.28 7.27 7.27 7.27 3.48 3.47 3.46 3.46 3.45 3.45 3.44 3.43 1.90 1.88 1.88 1.87 1.86 1.85 1.83 1.81 1.76 1.75 1.75 1.73 1.73 1.71 1.69 1.67 1.11 1.09 1.08
Et
SPh
O
H
6
1H, CDCl3, 400 MHz
1.00
2.10
3.26
1.09
1.22
1.14
3.61
10.5 10.0 9.5 9.0 8.5 8.0 7.5 7.0 6.5 6.0 5.5 5.0 4.5 4.0 3.5 3.0 2.5 2.0 1.5 1.0 0.5 0.0 -0.5 -1
f1 (ppm)

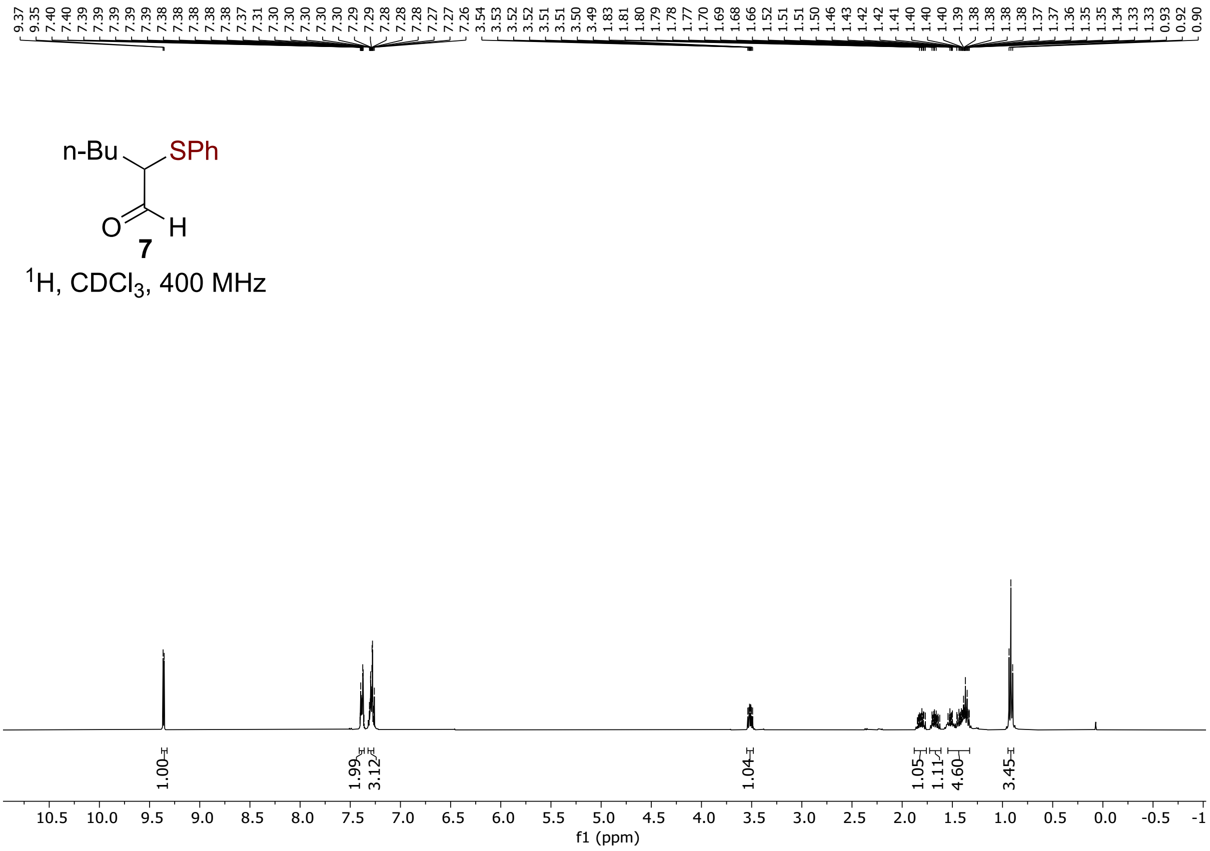
9.37 9.35 7.40 7.40 7.39 7.39 7.39 7.39 7.39 7.38 7.38 7.38 7.38 7.38 7.37 7.31 7.30 7.30 7.30 7.30 7.29 7.29 7.28 7.28 7.28 7.27 7.26 3.54 3.53 3.52 3.51 3.51 3.50 3.49 1.83 1.81 1.80 1.79 1.78 1.77 1.70 1.69 1.68 1.66 1.52 1.51 1.51 1.50 1.46 1.43 1.42 1.42 1.41 1.40 1.40 1.40 1.39 1.38 1.38 1.38 1.38 1.37 1.36 1.35 1.35 1.34 1.33 0.93 0.92 0.90
n-Bu
SPh
O
H
7
1H, CDCl3, 400 MHz
1.00
1.99
3.12
1.04
1.05
1.11
4.60
3.45
10.5 10.0 9.5 9.0 8.5 8.0 7.5 7.0 6.5 6.0 5.5 5.0 4.5 4.0 3.5 3.0 2.5 2.0 1.5 1.0 0.5 0.0 -0.5 -1
f1 (ppm)

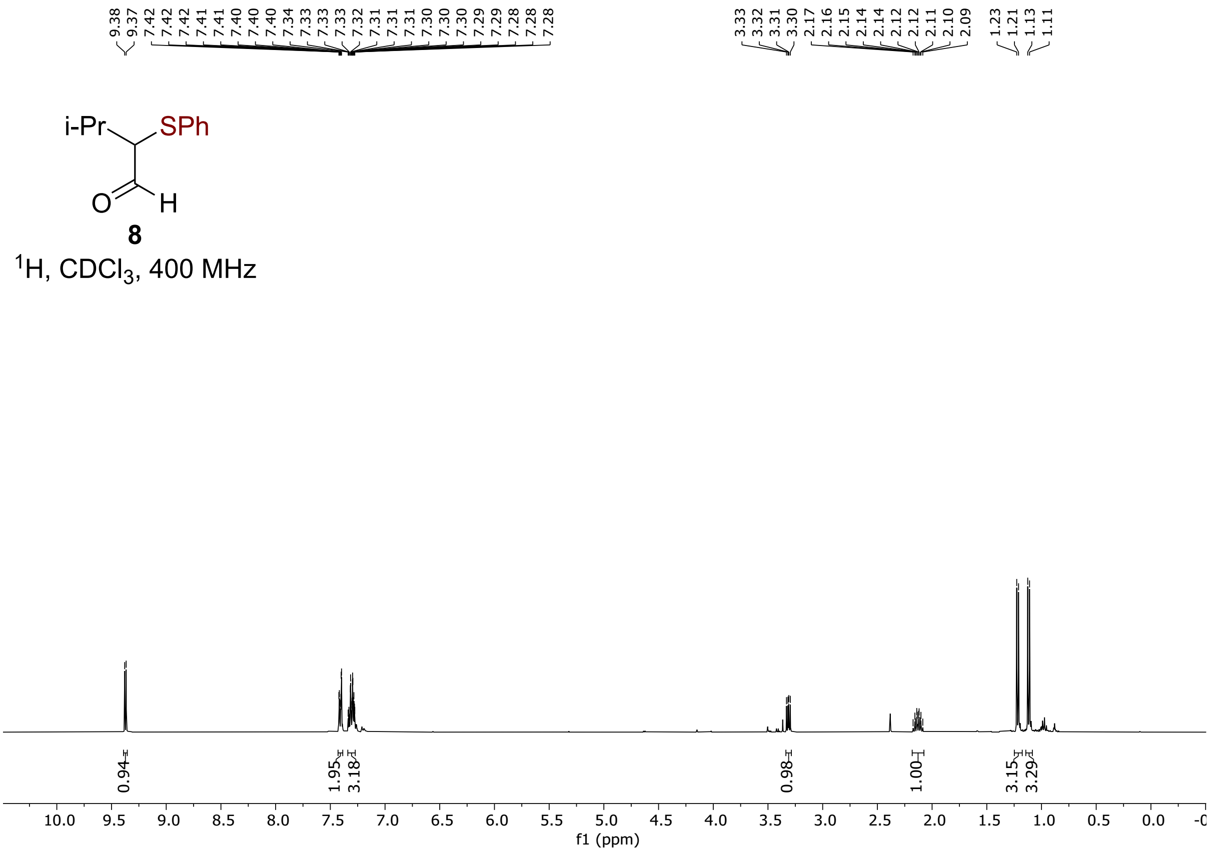
9.38 9.37 7.42 7.42 7.41 7.41 7.40 7.40 7.40 7.34 7.33 7.33 7.33 7.32 7.31 7.31 7.30 7.30 7.30 7.29 7.29 7.28 7.28 7.28
3.33 3.32 3.31 3.30 2.17 2.16 2.15 2.14 2.14 2.12 2.12 2.11 2.10 2.09 1.23 1.21 1.13 1.11
i-Pr
SPh
O
H
8
^{1}H, CDCl$_3$, 400 MHz
0.94
1.95
3.18
0.98
1.00
3.15
3.29
f1 (ppm)

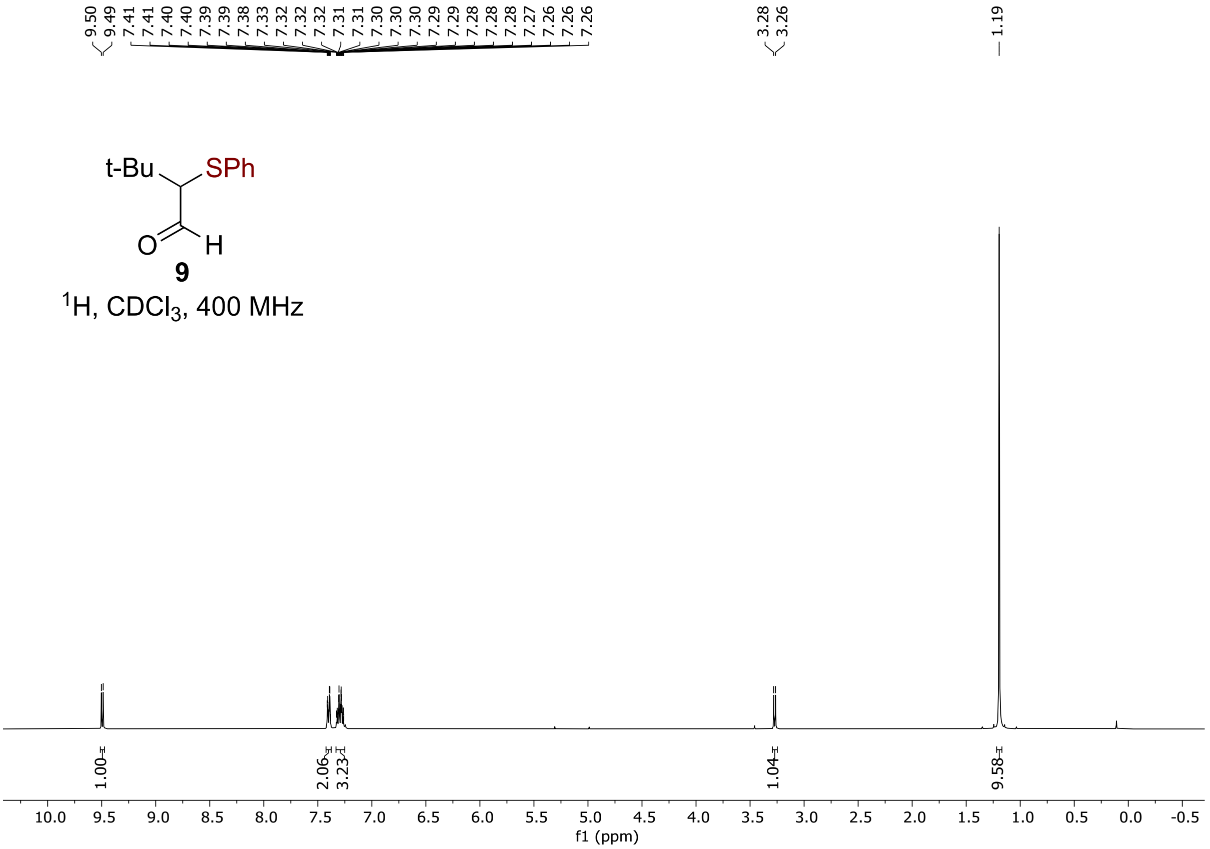
9.50 9.49 7.41 7.41 7.40 7.40 7.39 7.39 7.38 7.33 7.32 7.32 7.32 7.31 7.31 7.30 7.30 7.30 7.29 7.29 7.28 7.28 7.28 7.27 7.26 7.26 7.26
3.28 3.26
1.19
t-Bu
SPh
O
H
9
^{1}H, CDCl$_3$, 400 MHz
1.00
2.06
3.23
1.04
9.58
f1 (ppm)

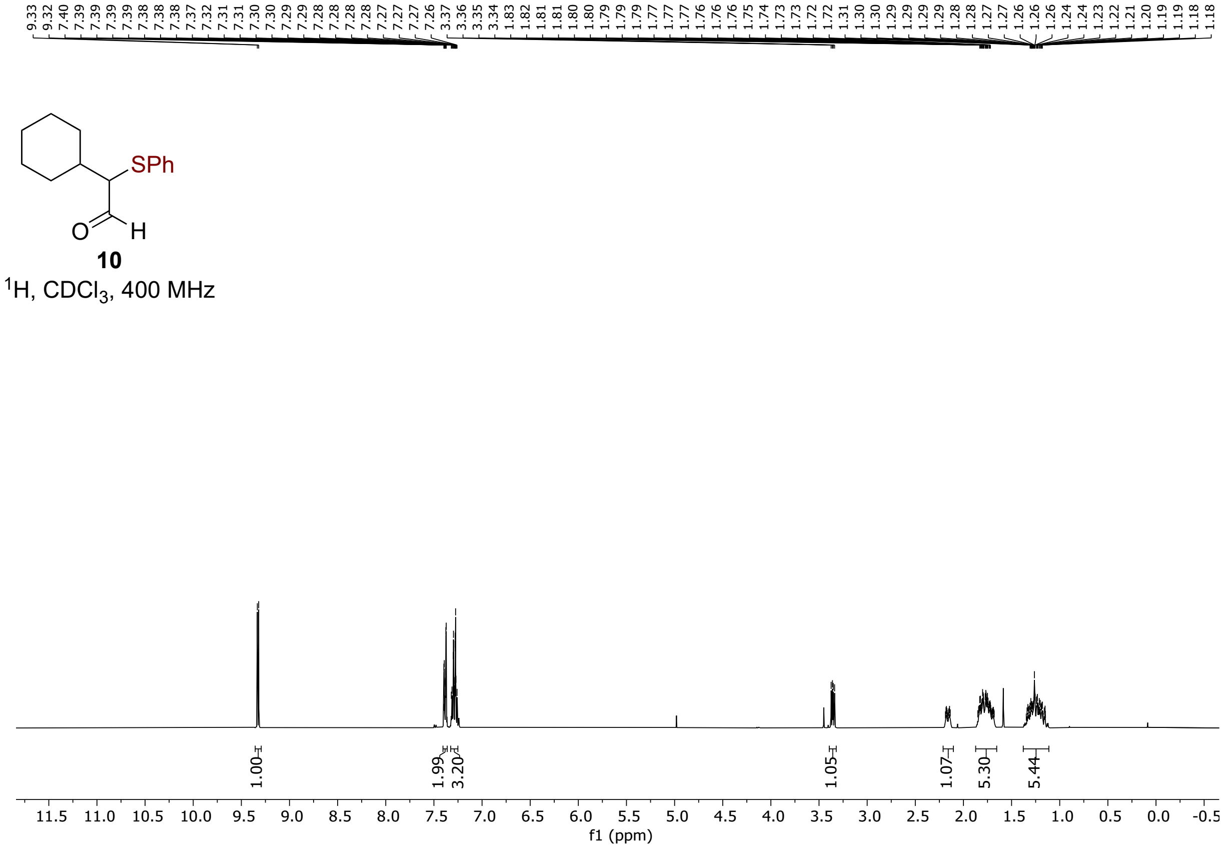
9.33 9.32 7.40 7.39 7.39 7.39 7.39 7.38 7.38 7.38 7.37 7.32 7.31 7.31 7.30 7.30 7.29 7.29 7.28 7.28 7.28 7.28 7.27 7.27 7.27 7.26 3.37 3.36 3.35 3.34 1.83 1.82 1.81 1.81 1.80 1.80 1.79 1.79 1.79 1.77 1.77 1.77 1.76 1.76 1.76 1.75 1.74 1.73 1.73 1.72 1.72 1.31 1.30 1.30 1.29 1.29 1.29 1.29 1.29 1.28 1.28 1.27 1.27 1.26 1.26 1.26 1.24 1.24 1.23 1.22 1.21 1.20 1.19 1.18 1.18
SPh
O
H
10
^{1}H, CDCl$_3$, 400 MHz
1.00
1.99
3.20
1.05
1.07
5.30
5.44
11.5 11.0 10.5 10.0 9.5 9.0 8.5 8.0 7.5 7.0 6.5 6.0 5.5 5.0 4.5 4.0 3.5 3.0 2.5 2.0 1.5 1.0 0.5 0.0 -0.5
f1 (ppm)

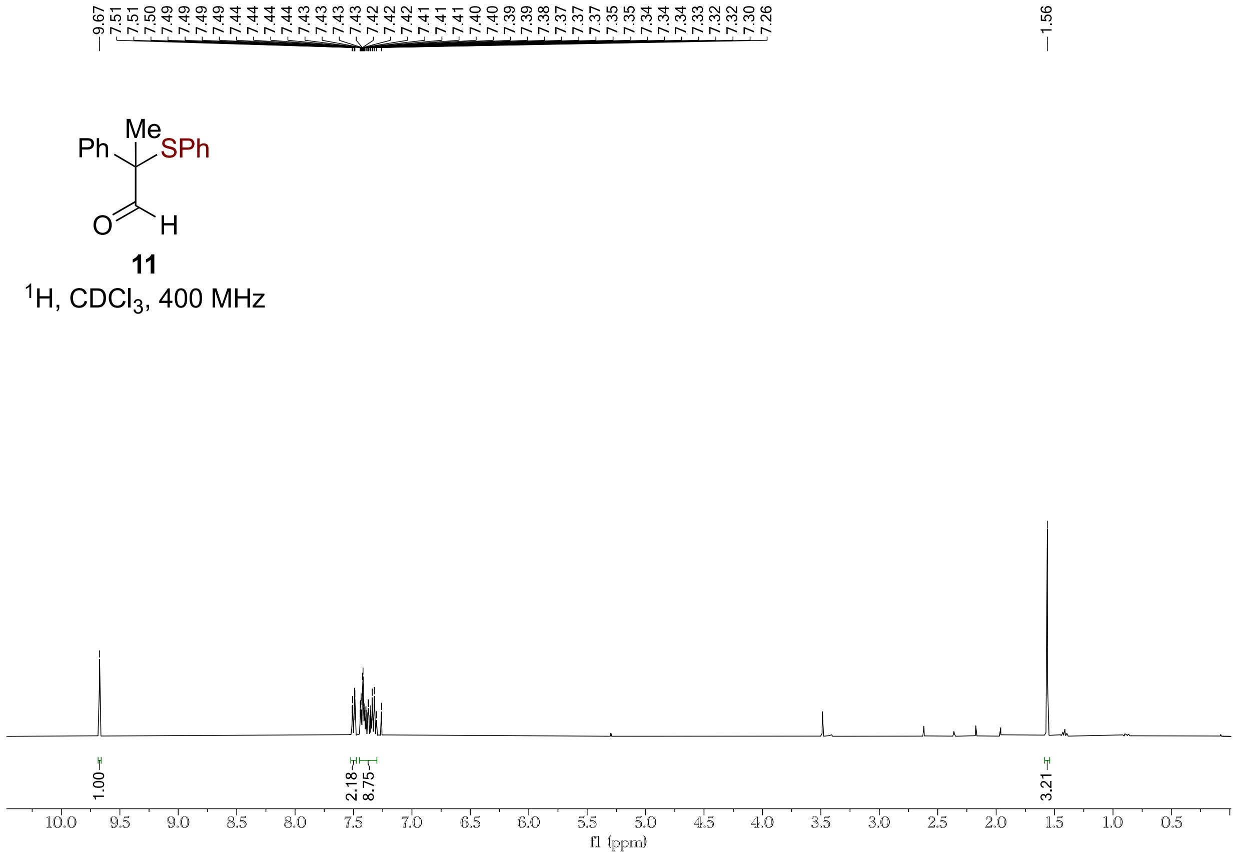
9.67 7.51 7.51 7.50 7.49 7.49 7.49 7.49 7.44 7.44 7.44 7.44 7.43 7.43 7.43 7.43 7.42 7.42 7.42 7.41 7.41 7.40 7.39 7.39 7.38 7.37 7.37 7.37 7.35 7.34 7.34 7.33 7.32 7.32 7.30 7.26
1.56
Me
Ph
SPh
O
H
11
^{1}H, CDCl$_3$, 400 MHz
1.00
2.18
8.75
3.21
10.0 9.5 9.0 8.5 8.0 7.5 7.0 6.5 6.0 5.5 5.0 4.5 4.0 3.5 3.0 2.5 2.0 1.5 1.0 0.5
f1 (ppm)

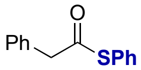

12

^{1}H, $CDCl_3$, 400 MHz

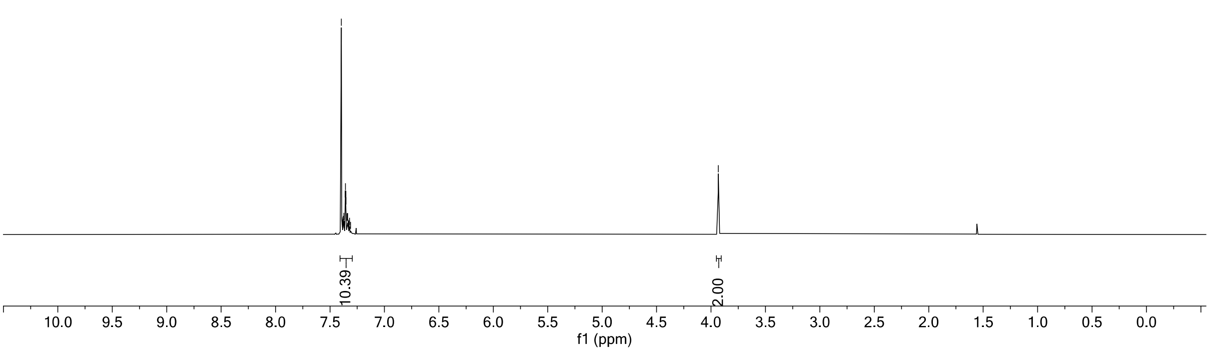

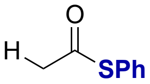

17

^{1}H, $CDCl_3$, 400 MHz

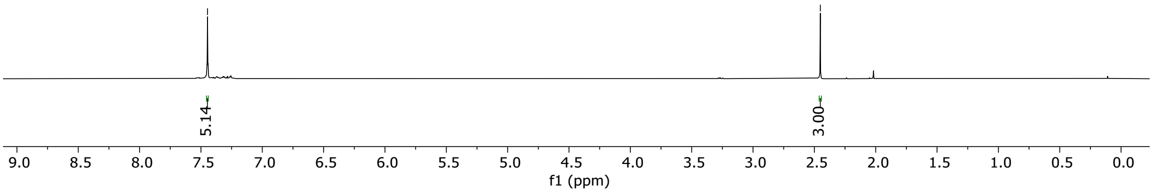

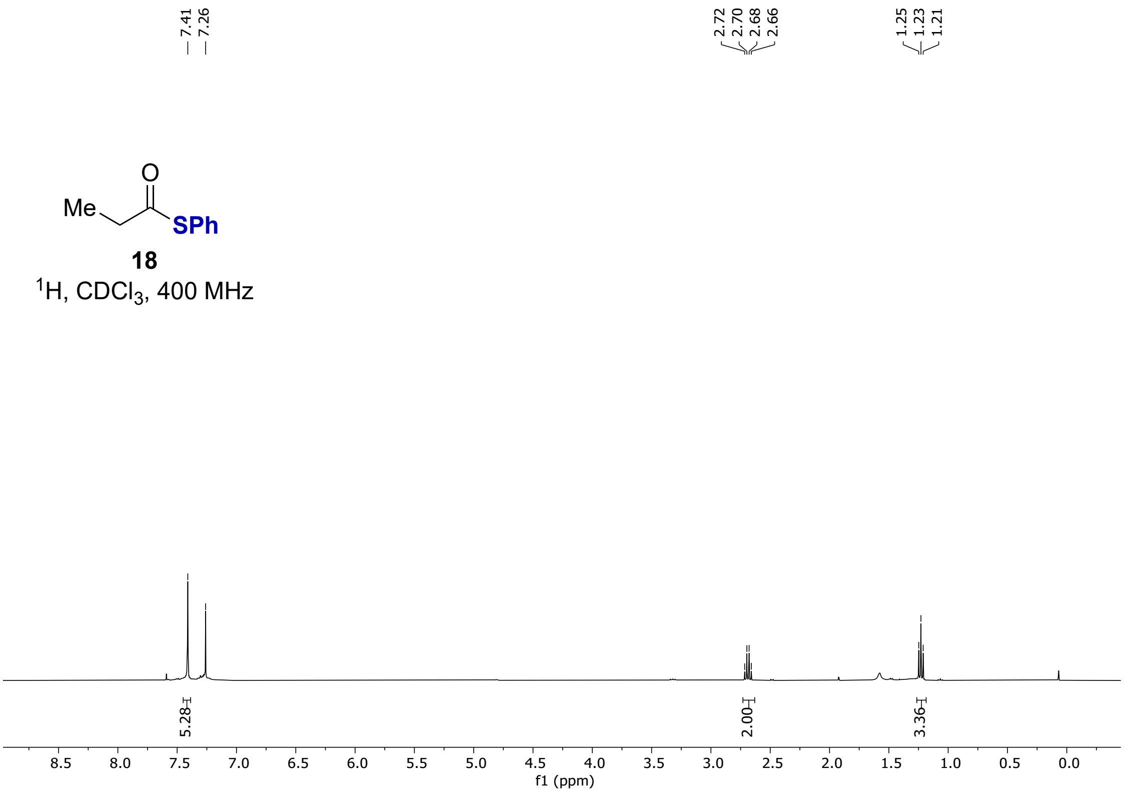
7.41
7.26
2.72
2.70
2.68
2.66
1.25
1.23
1.21
O
Me
SPh
18
^{1}H, $CDCl_3$, 400 MHz
5.28
2.00
3.36
f1 (ppm)

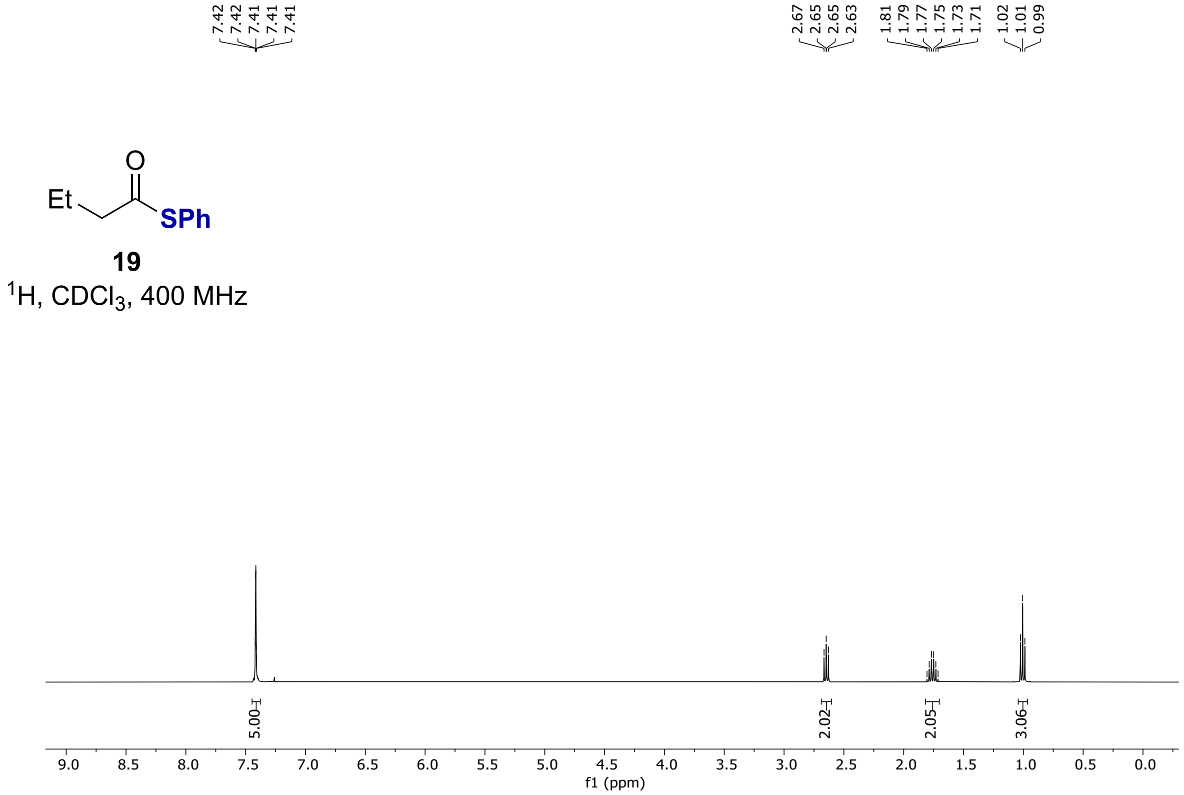
7.42
7.42
7.41
7.41
7.41
2.67
2.65
2.65
2.63
1.81
1.79
1.77
1.75
1.73
1.71
1.02
1.01
0.99
O
Et
SPh
19
^{1}H, $CDCl_3$, 400 MHz
5.00
2.02
2.05
3.06
f1 (ppm)

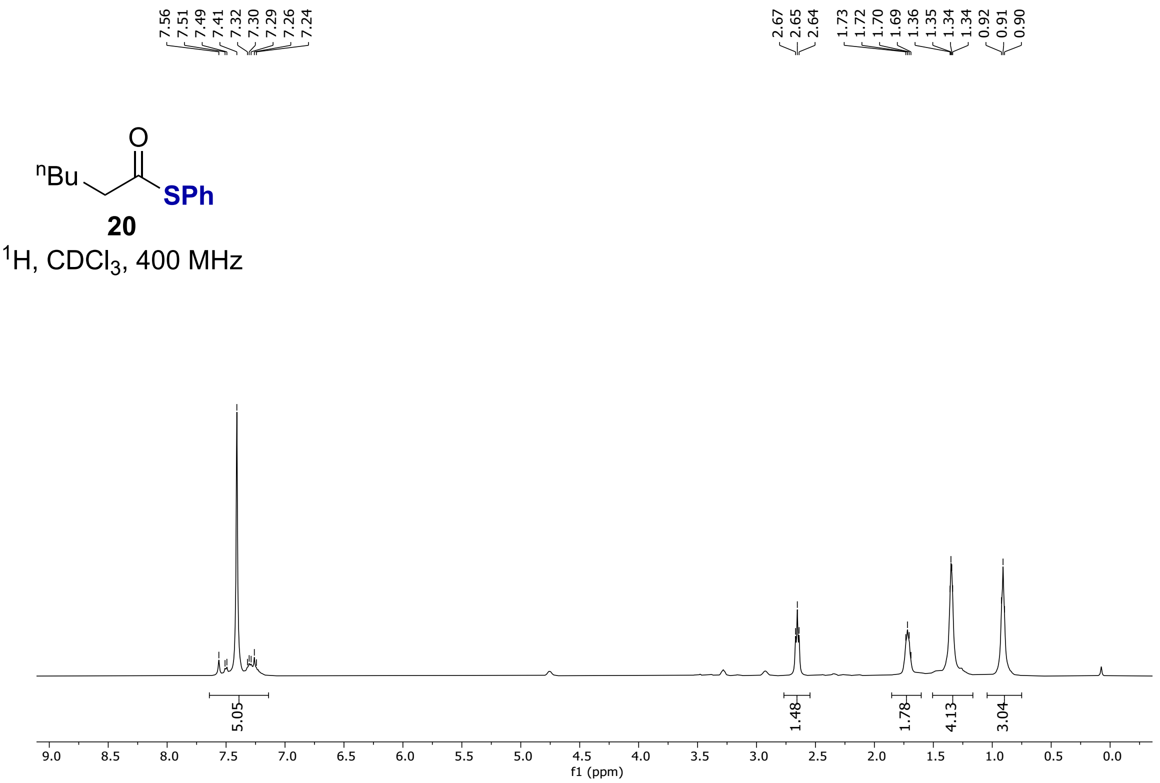
nBu
SPh
20
1H, CDCl3, 400 MHz
f1 (ppm)

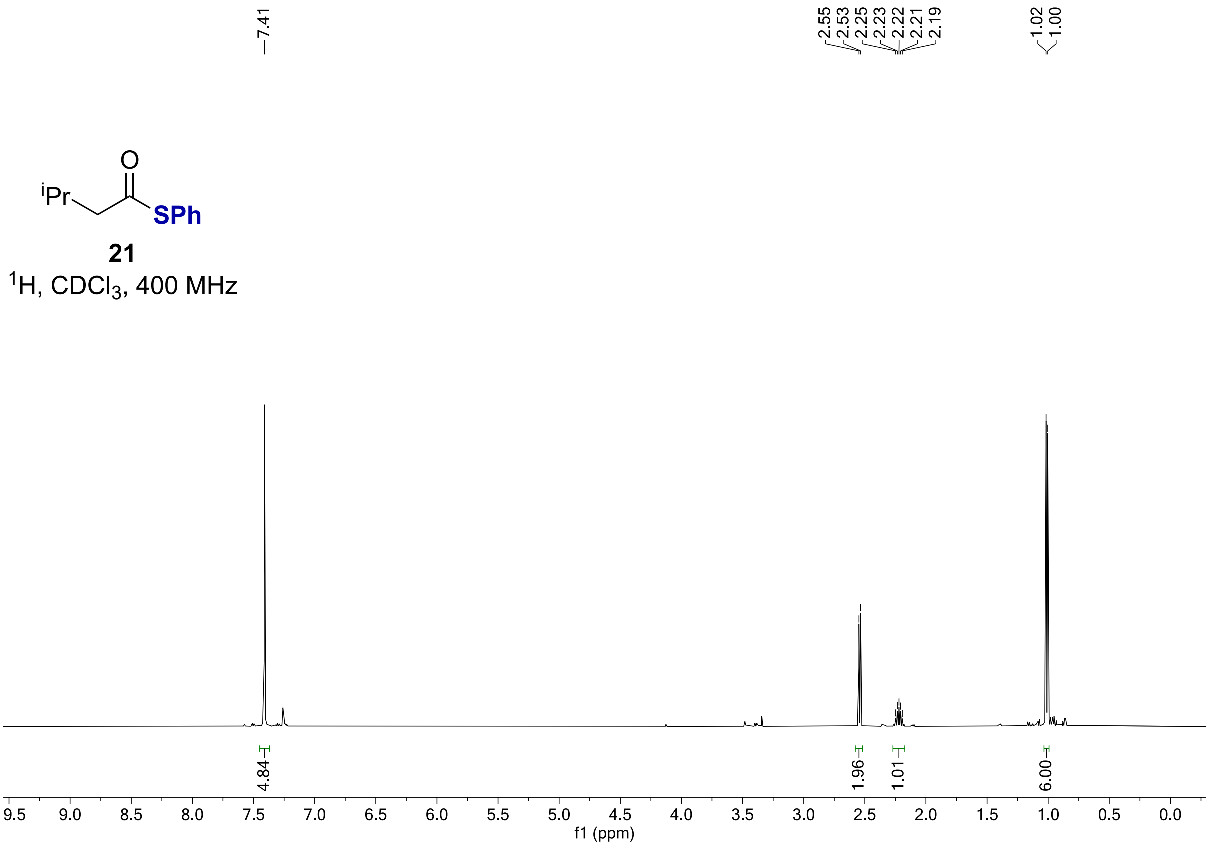
iPr
SPh
21
1H, CDCl3, 400 MHz
f1 (ppm)

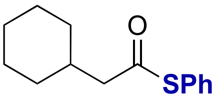

22

^{1}H, $CDCl_3$, 400 MHz

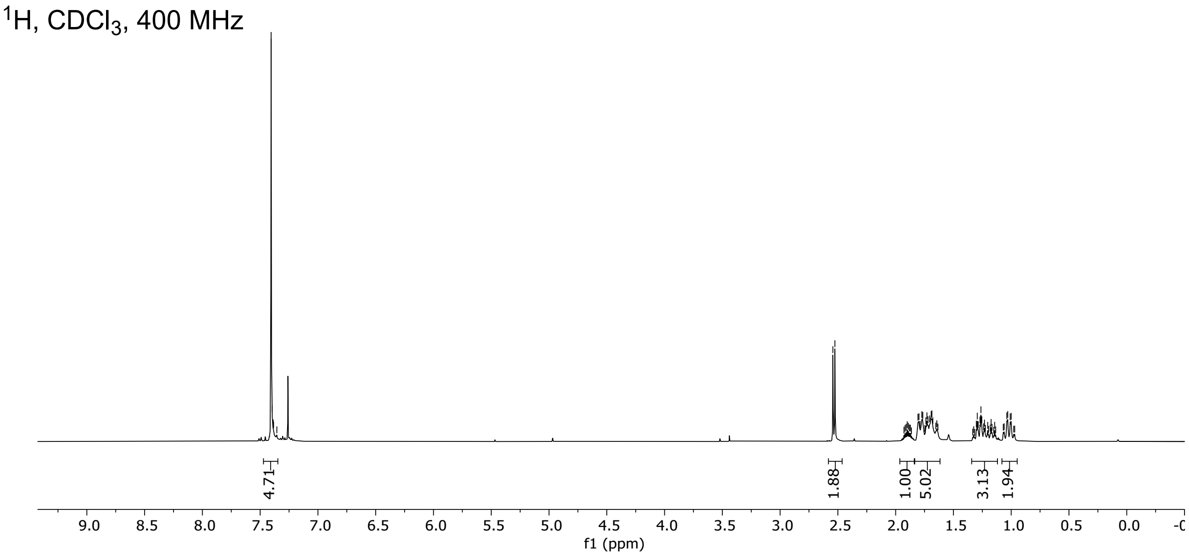

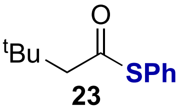

23

^{1}H, $CDCl_3$, 400 MHz

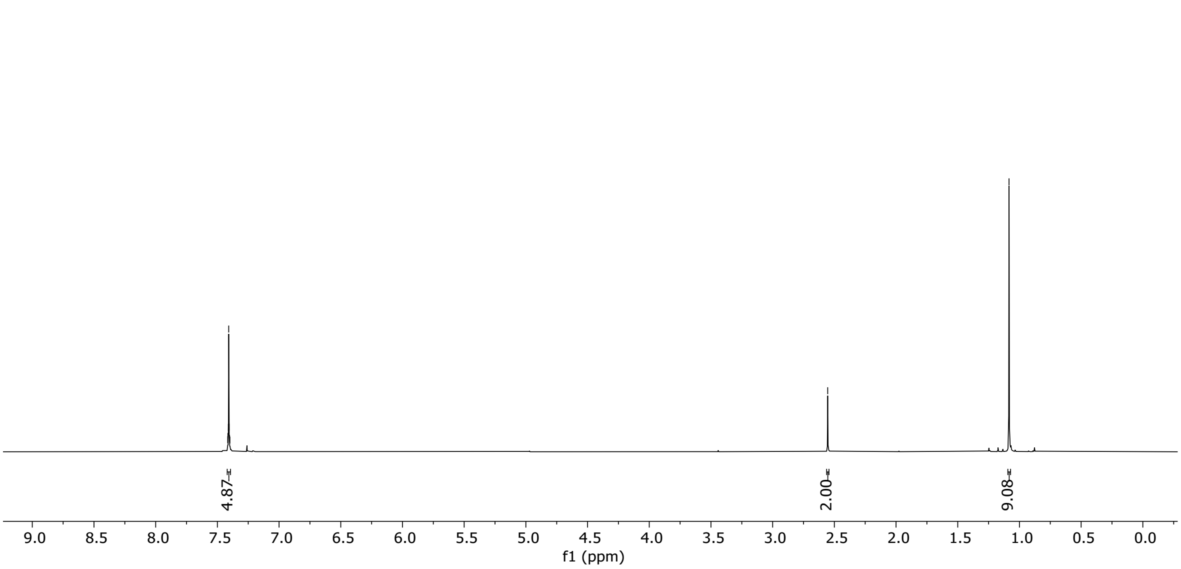

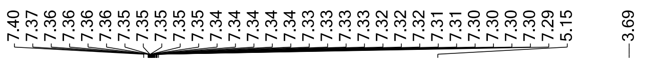

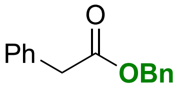

24

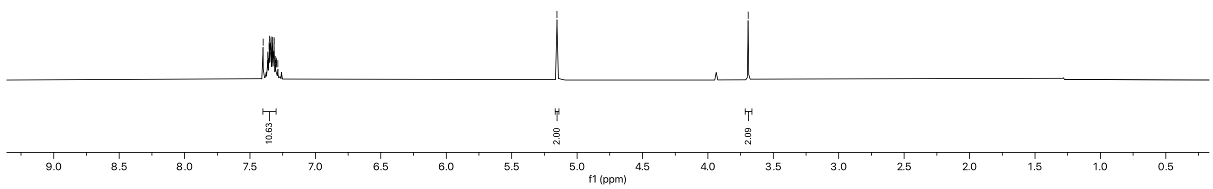

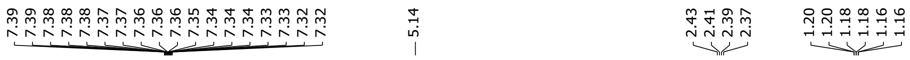

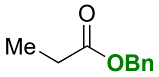

25

^1H, $CDCl_3$, 400 MHz

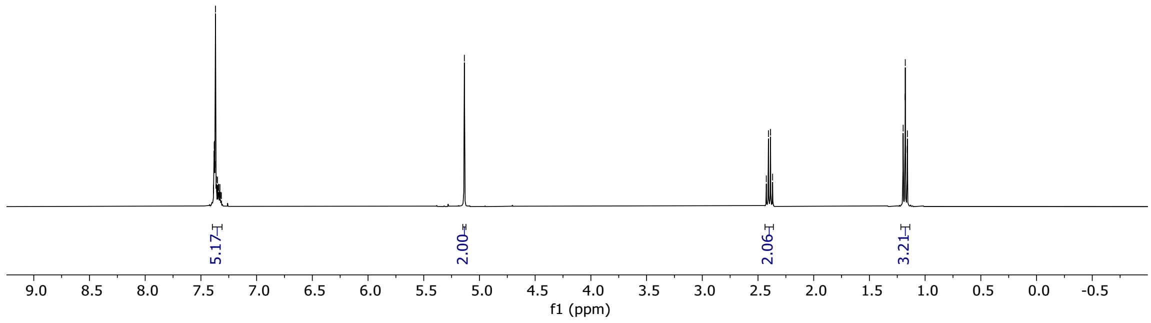

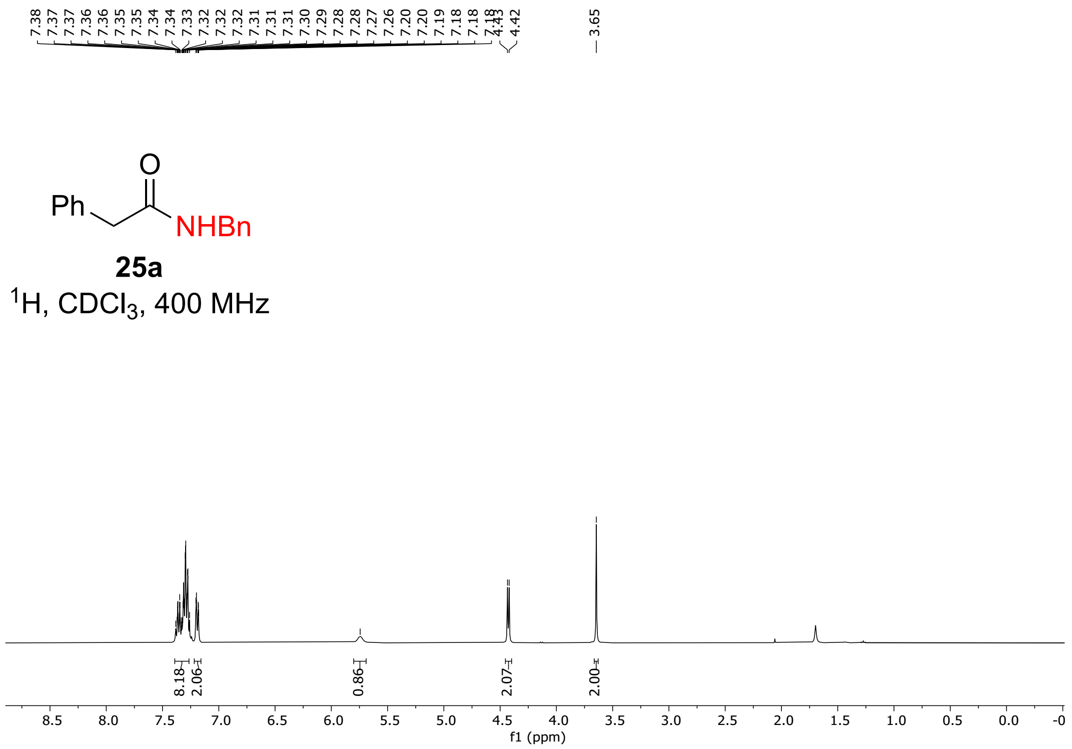

25a
^1H, $CDCl_3$, 400 MHz

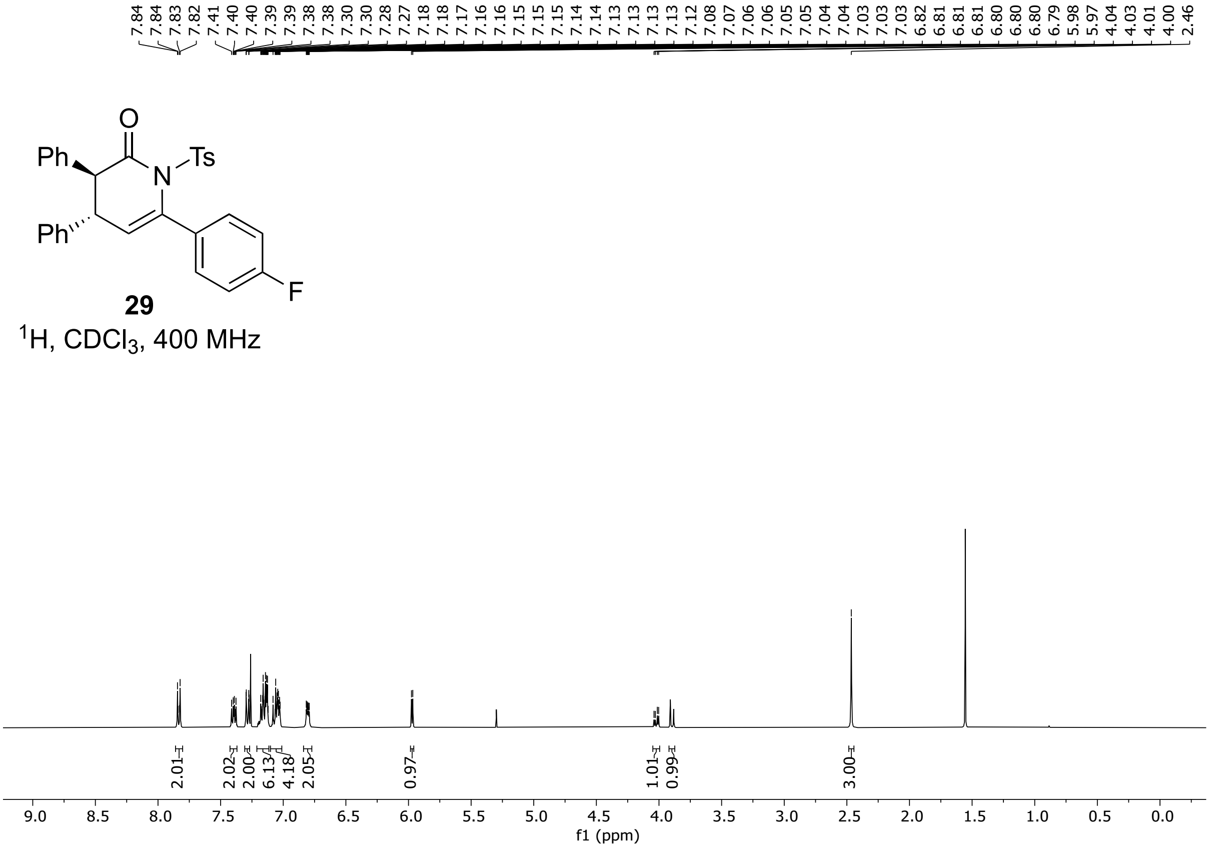

29
^1H, $CDCl_3$, 400 MHz

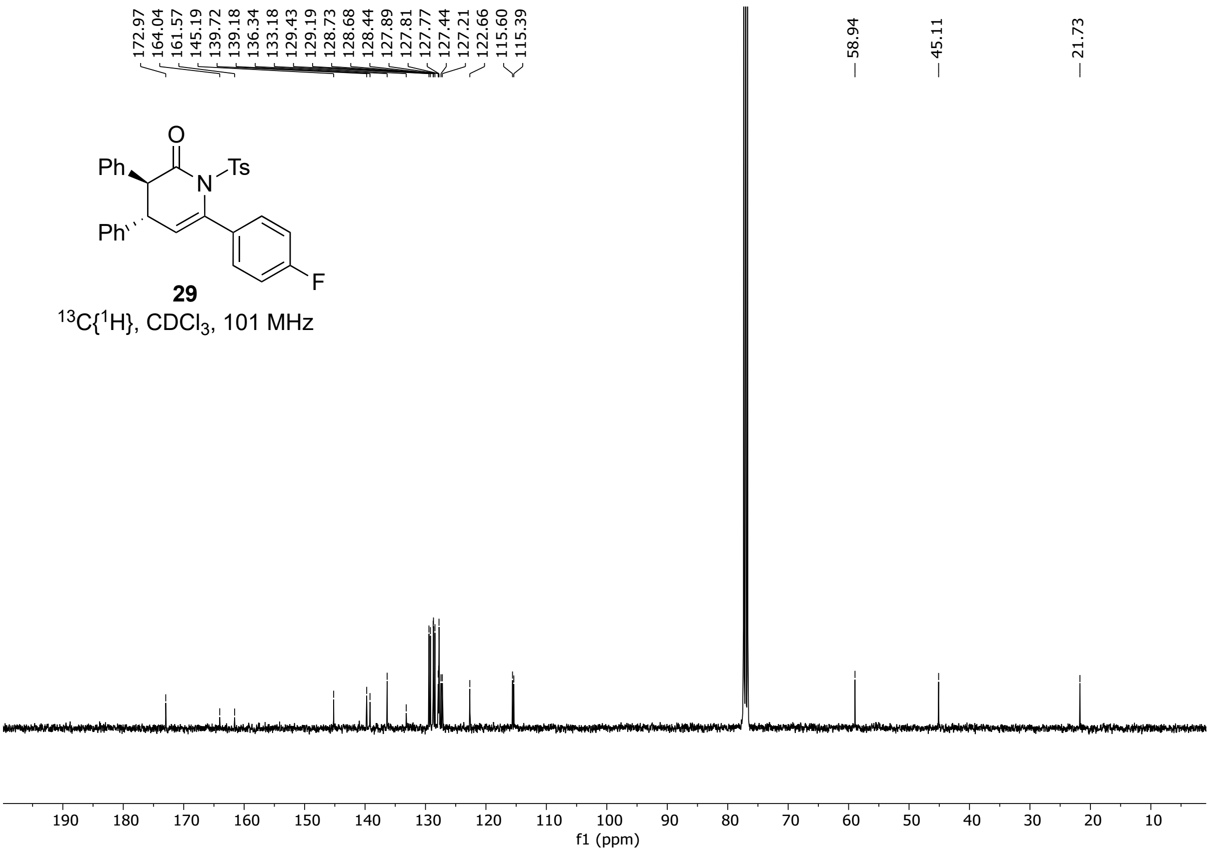
172.97
164.04
161.57
145.19
139.72
139.18
136.34
133.18
129.43
129.19
128.73
128.68
128.44
127.89
127.81
127.77
127.44
127.21
122.66
115.60
115.39
58.94
45.11
21.73
O
Ph
N
Ts
Ph
F
29
$^{13}C\{^1H\}$, $CDCl_3$, 101 MHz
190 180 170 160 150 140 130 120 110 100 90 80 70 60 50 40 30 20 10
f1 (ppm)

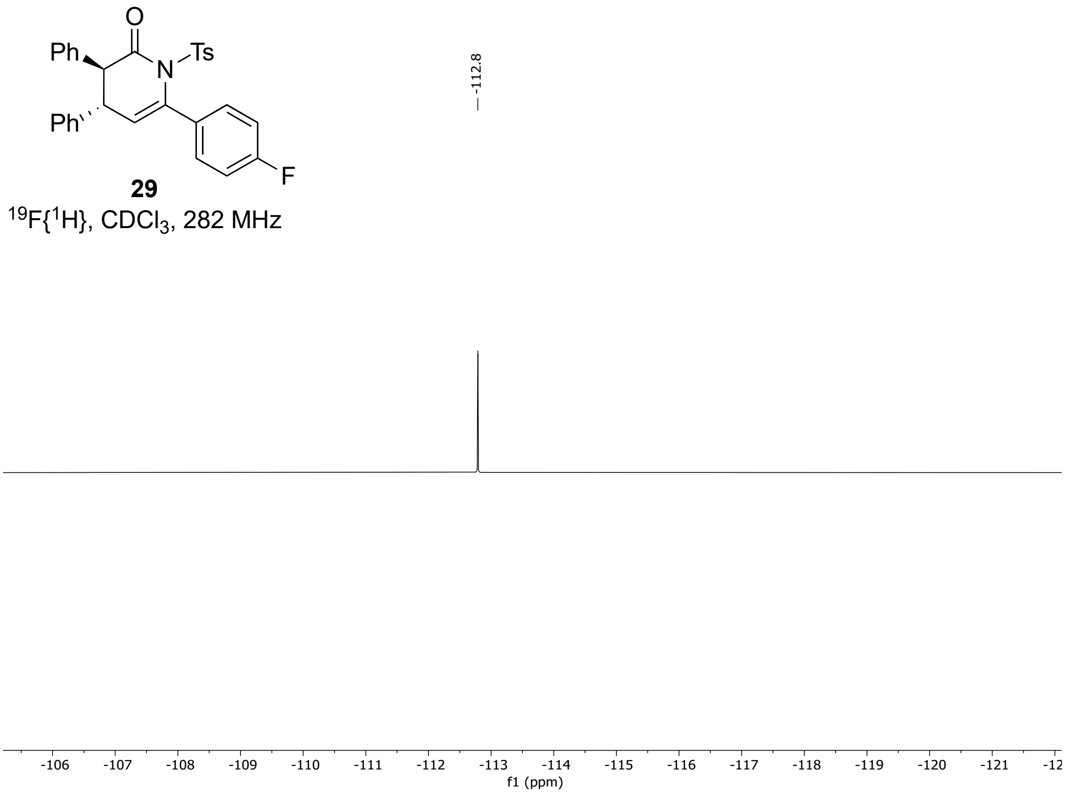
O
Ph
N
Ts
Ph
F
29
$^{19}F\{^1H\}$, $CDCl_3$, 282 MHz
-112.8
-106 -107 -108 -109 -110 -111 -112 -113 -114 -115 -116 -117 -118 -119 -120 -121 -12
f1 (ppm)

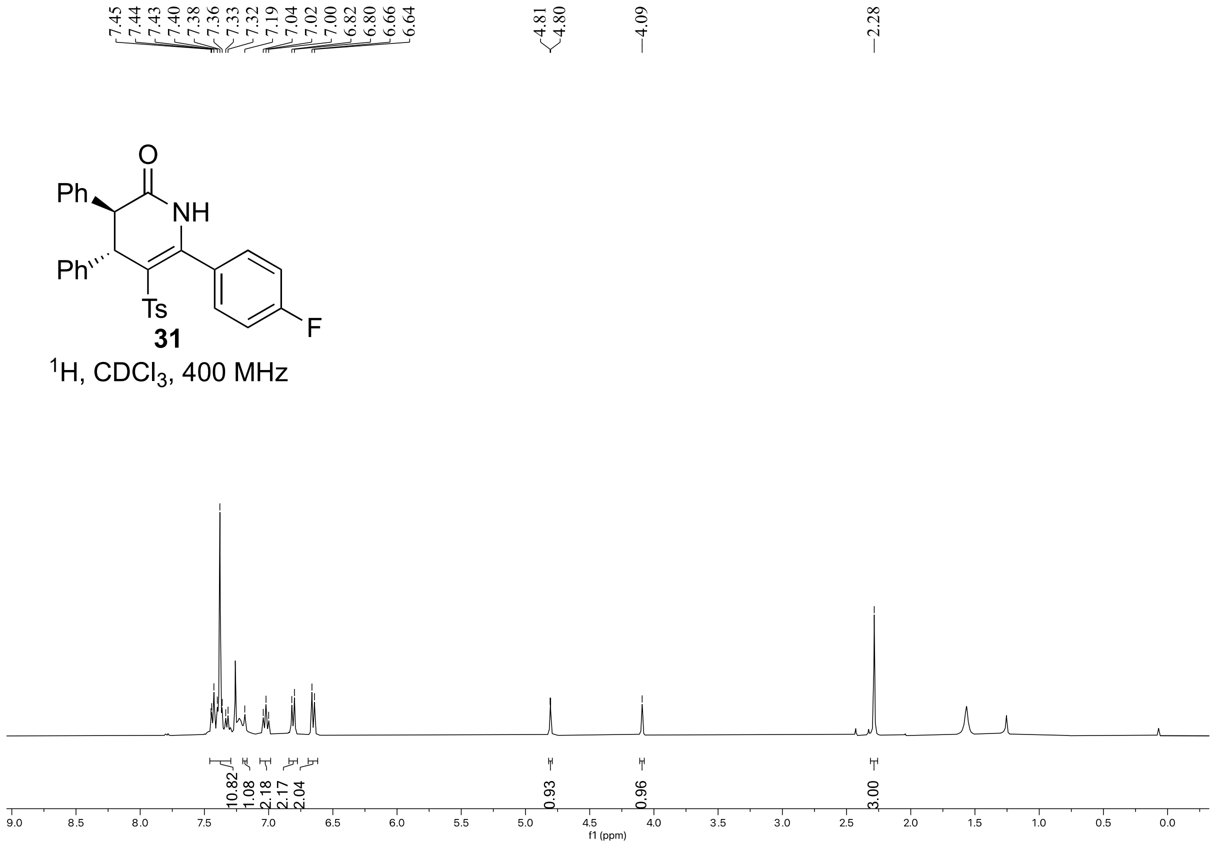
7.45
7.44
7.43
7.40
7.38
7.36
7.33
7.32
7.19
7.04
7.02
7.00
6.82
6.80
6.66
6.64
4.81
4.80
4.09
2.28
O
Ph
NH
Ph
Ts
F
31
1H, CDCl3, 400 MHz
10.82
1.08
2.18
2.17
2.04
0.93
0.96
3.00
f1 (ppm)

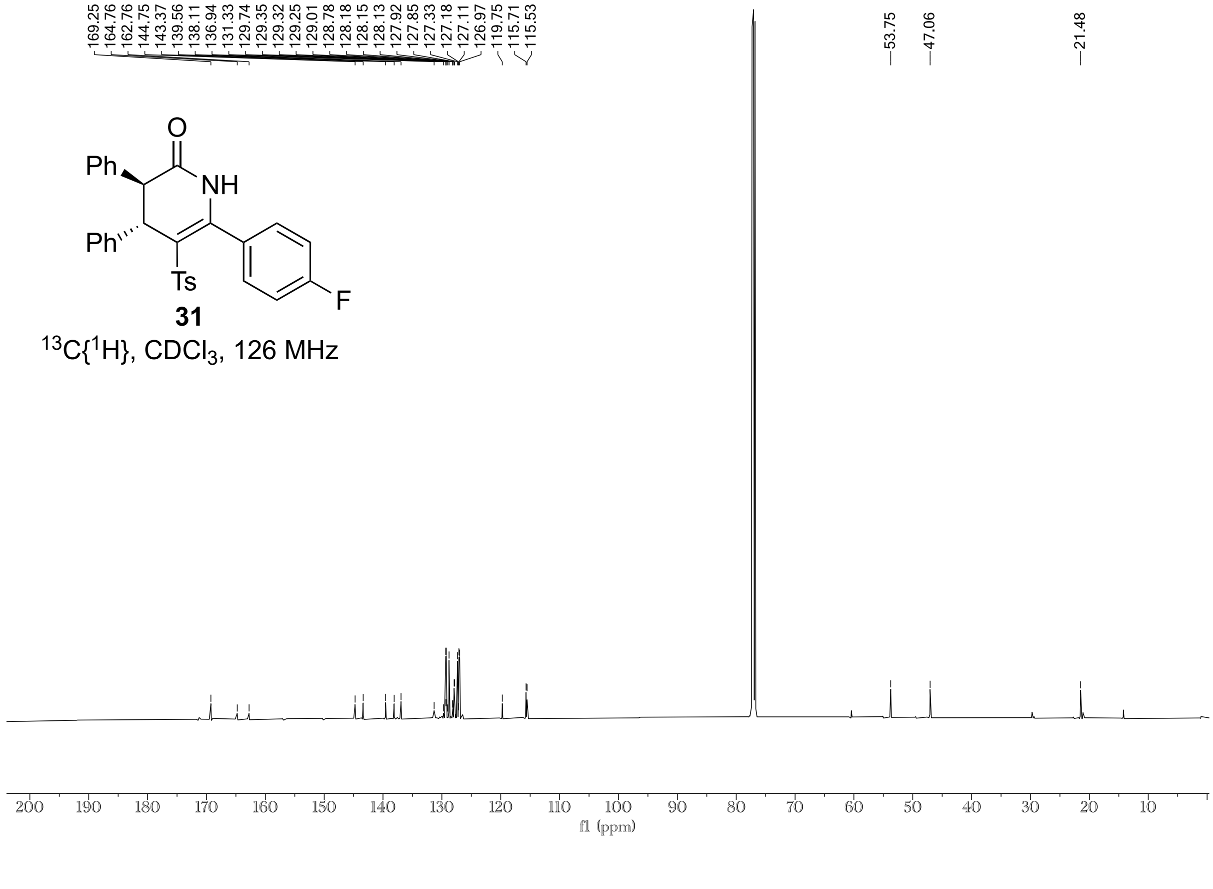
169.25
164.76
162.76
144.75
143.37
139.56
138.11
136.94
131.33
129.74
129.35
129.32
129.25
129.01
128.78
128.18
128.15
128.13
127.92
127.85
127.33
127.18
127.11
126.97
119.75
115.71
115.53
53.75
47.06
21.48
O
Ph
NH
Ph
Ts
F
31
13C{1H}, CDCl3, 126 MHz
f1 (ppm)

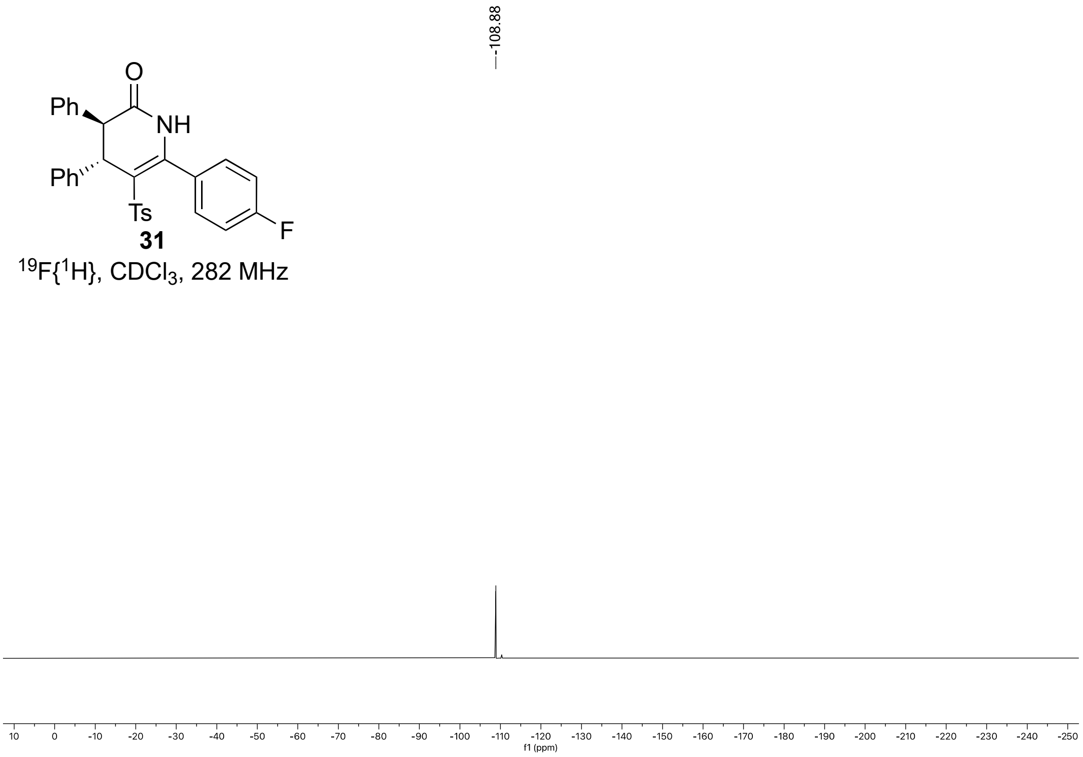

31
$^{19}F\{^1H\}$, $CDCl_3$, 282 MHz

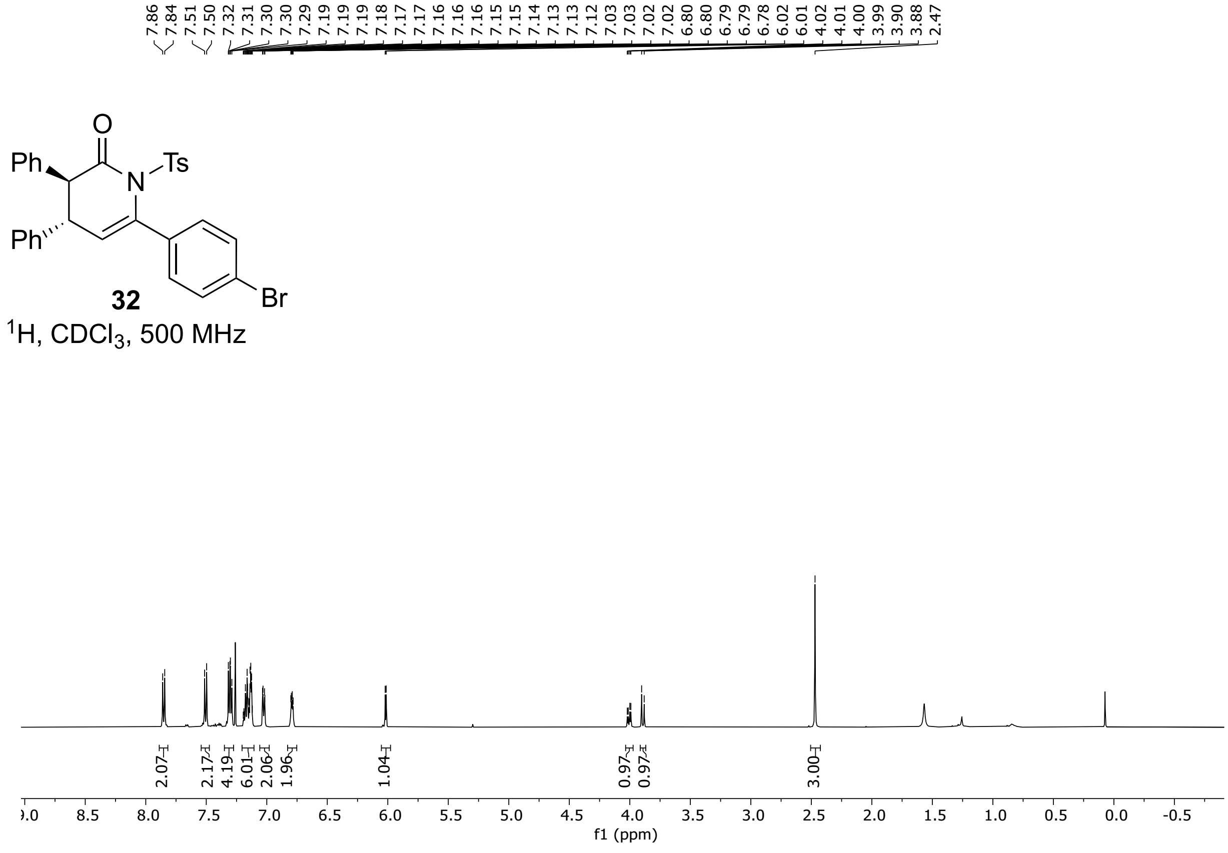

32
^1H, $CDCl_3$, 500 MHz

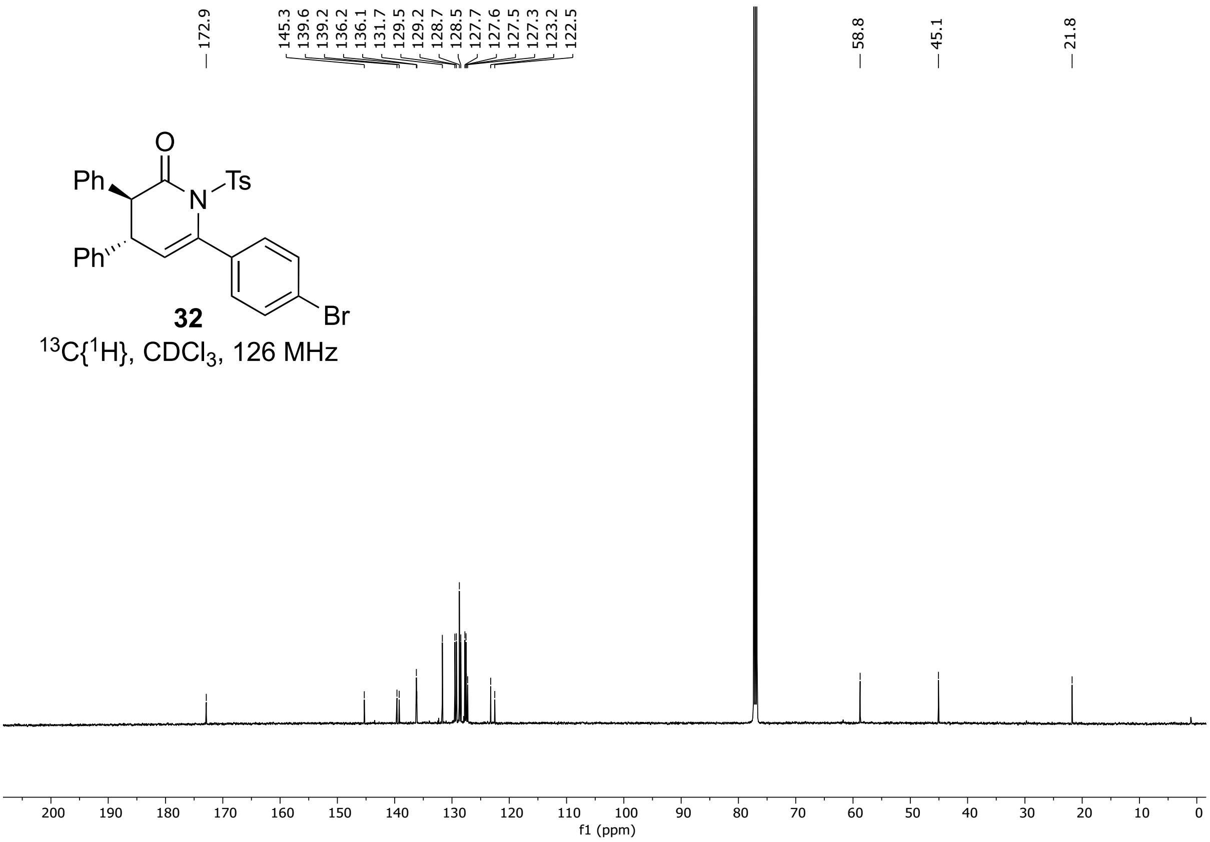
172.9
145.3
139.6
139.2
136.2
136.1
131.7
129.5
129.2
128.7
128.5
127.7
127.6
127.5
127.3
123.2
122.5
58.8
45.1
21.8
Ph
O
N
Ts
Ph
Br
32
$^{13}C\{^1H\}$, $CDCl_3$, 126 MHz
200 190 180 170 160 150 140 130 120 110 100 90 80 70 60 50 40 30 20 10 0
f1 (ppm)

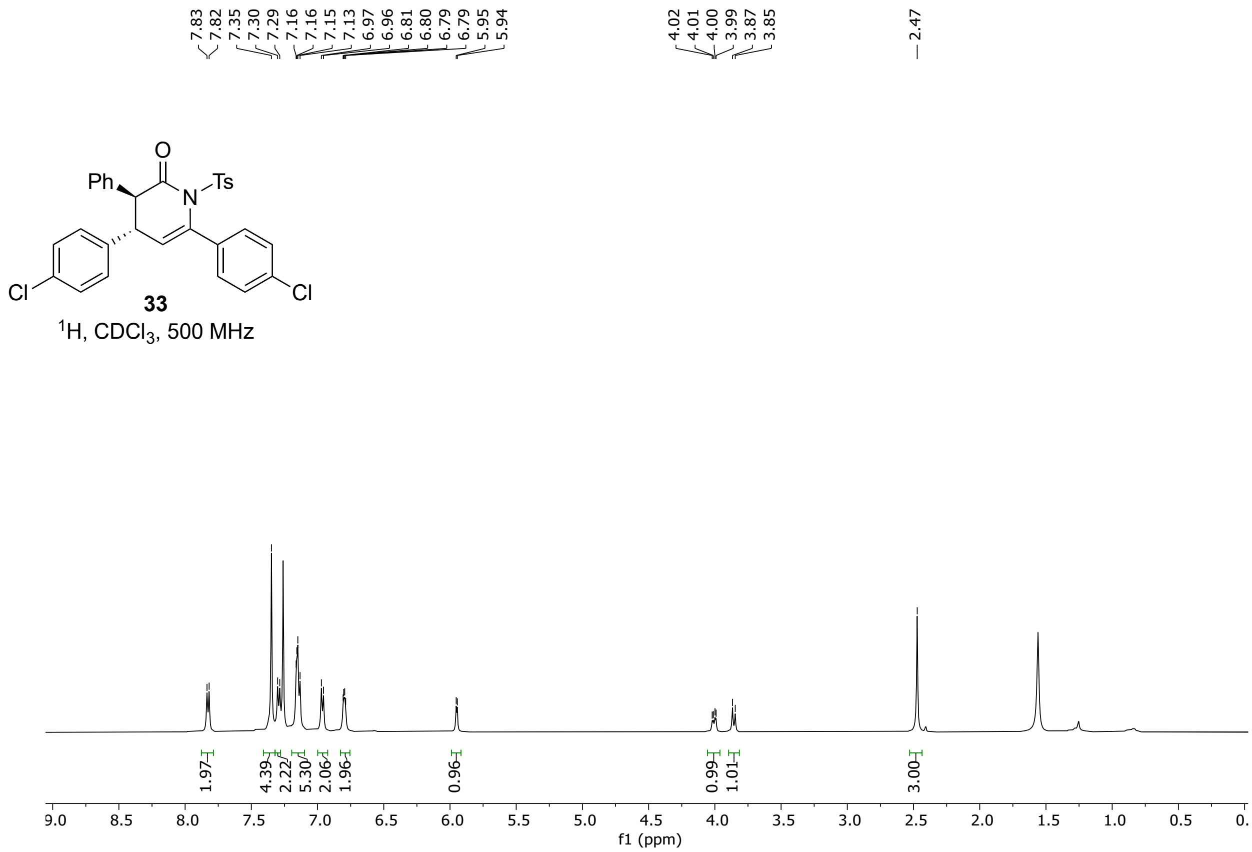
7.83
7.82
7.35
7.30
7.29
7.16
7.16
7.15
7.13
6.97
6.96
6.81
6.80
6.79
6.79
5.95
5.94
4.02
4.01
4.00
3.99
3.87
3.85
2.47
Ph
O
N
Ts
Cl
Cl
33
^1H, $CDCl_3$, 500 MHz
1.97
4.39
2.22
5.30
2.06
1.96
0.96
0.99
1.01
3.00
9.0 8.5 8.0 7.5 7.0 6.5 6.0 5.5 5.0 4.5 4.0 3.5 3.0 2.5 2.0 1.5 1.0 0.5 0.
f1 (ppm)

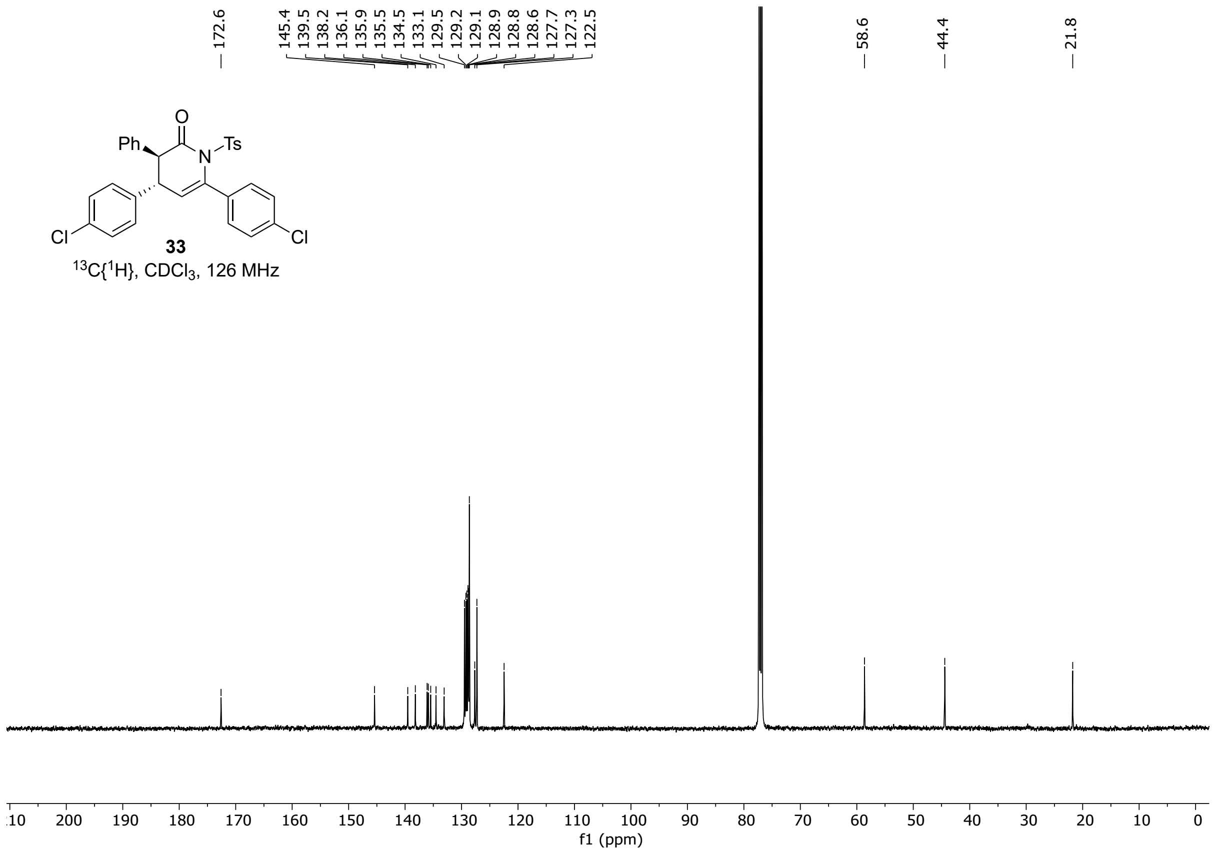
172.6
145.4
139.5
138.2
136.1
135.9
135.5
134.5
133.1
129.5
129.2
129.1
128.9
128.8
128.6
127.7
127.3
122.5
58.6
44.4
21.8
Ph
O
N
Ts
Cl
Cl
33
$^{13}C\{^{1}H\}$, $CDCl_3$, 126 MHz
f1 (ppm)

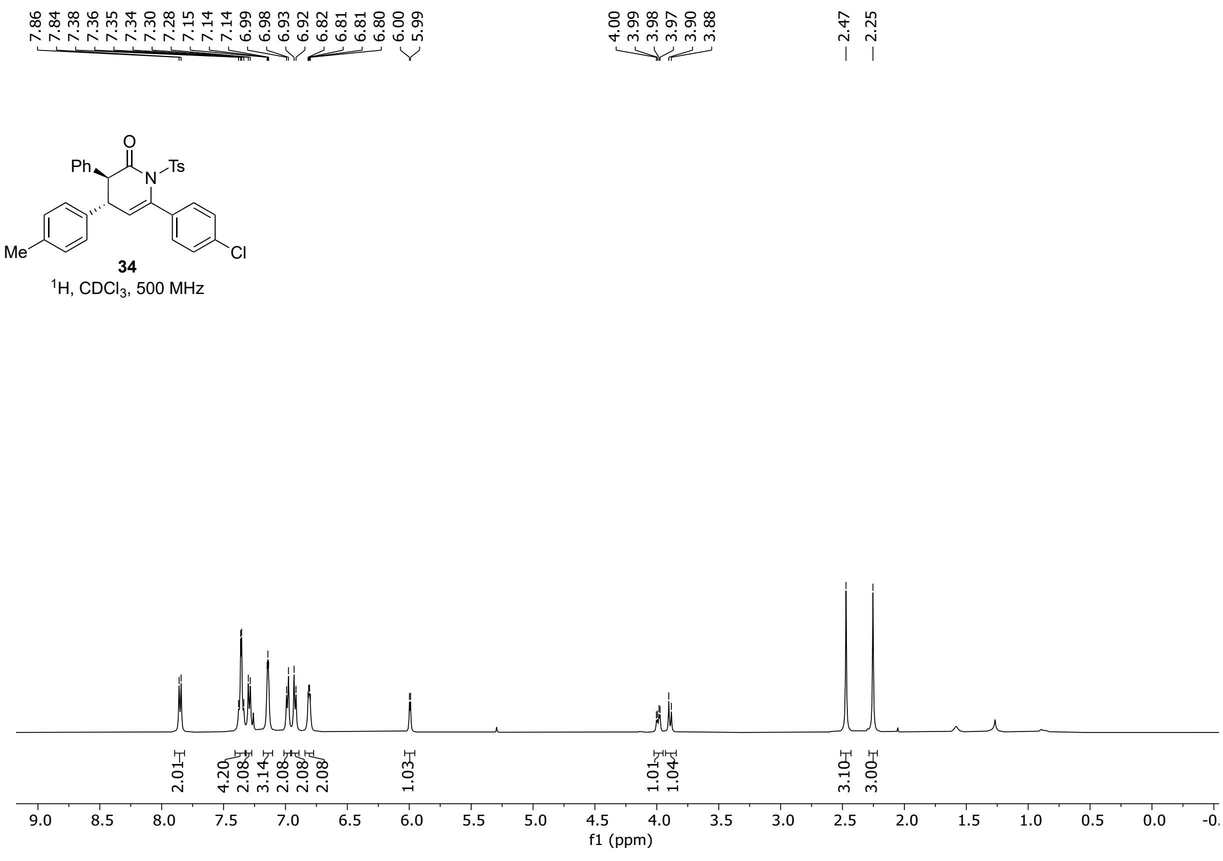
7.86
7.84
7.38
7.36
7.35
7.34
7.30
7.28
7.15
7.14
7.14
6.99
6.98
6.93
6.92
6.82
6.81
6.81
6.80
6.00
5.99
4.00
3.99
3.98
3.97
3.90
3.88
2.47
2.25
Ph
O
N
Ts
Me
Cl
34
^{1}H, $CDCl_3$, 500 MHz
2.01
4.20
2.08
3.14
2.08
2.08
2.08
1.03
1.01
1.04
3.10
3.00
f1 (ppm)

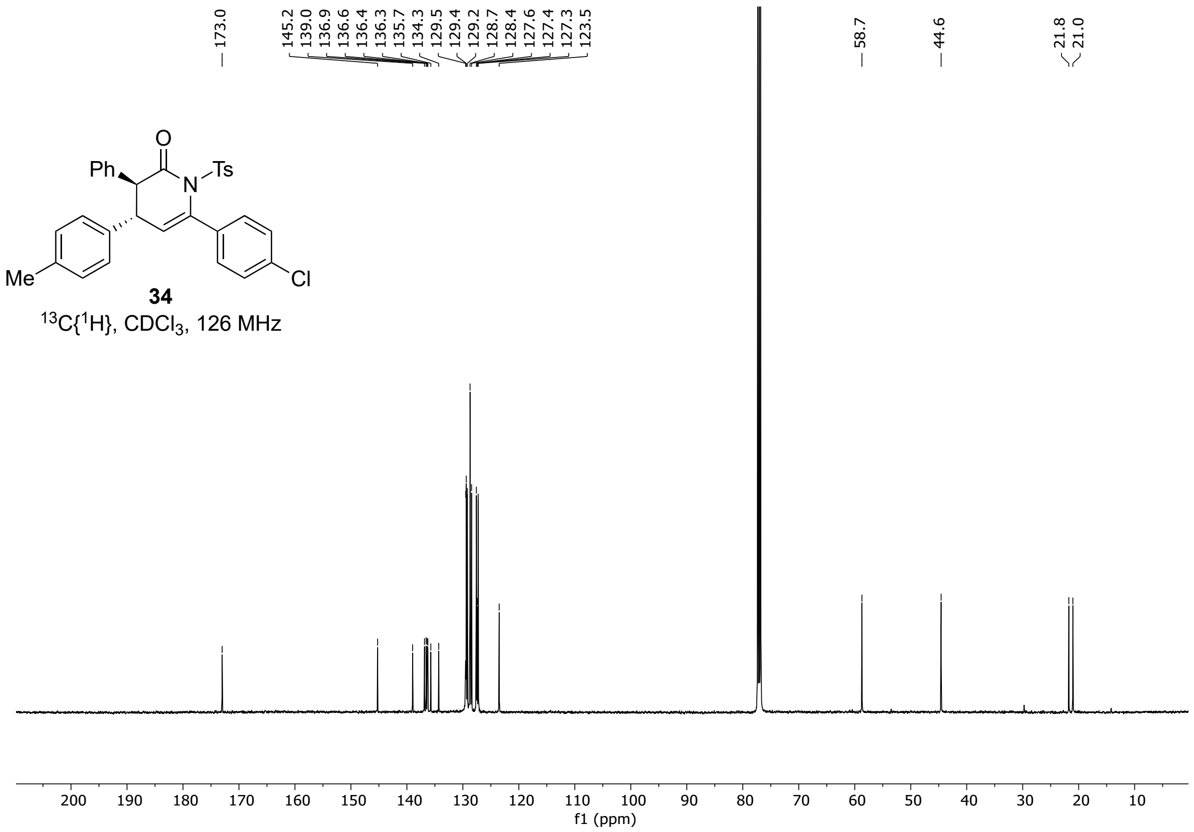

34

$^{13}C\{^{1}H\}$, $CDCl_3$, 126 MHz

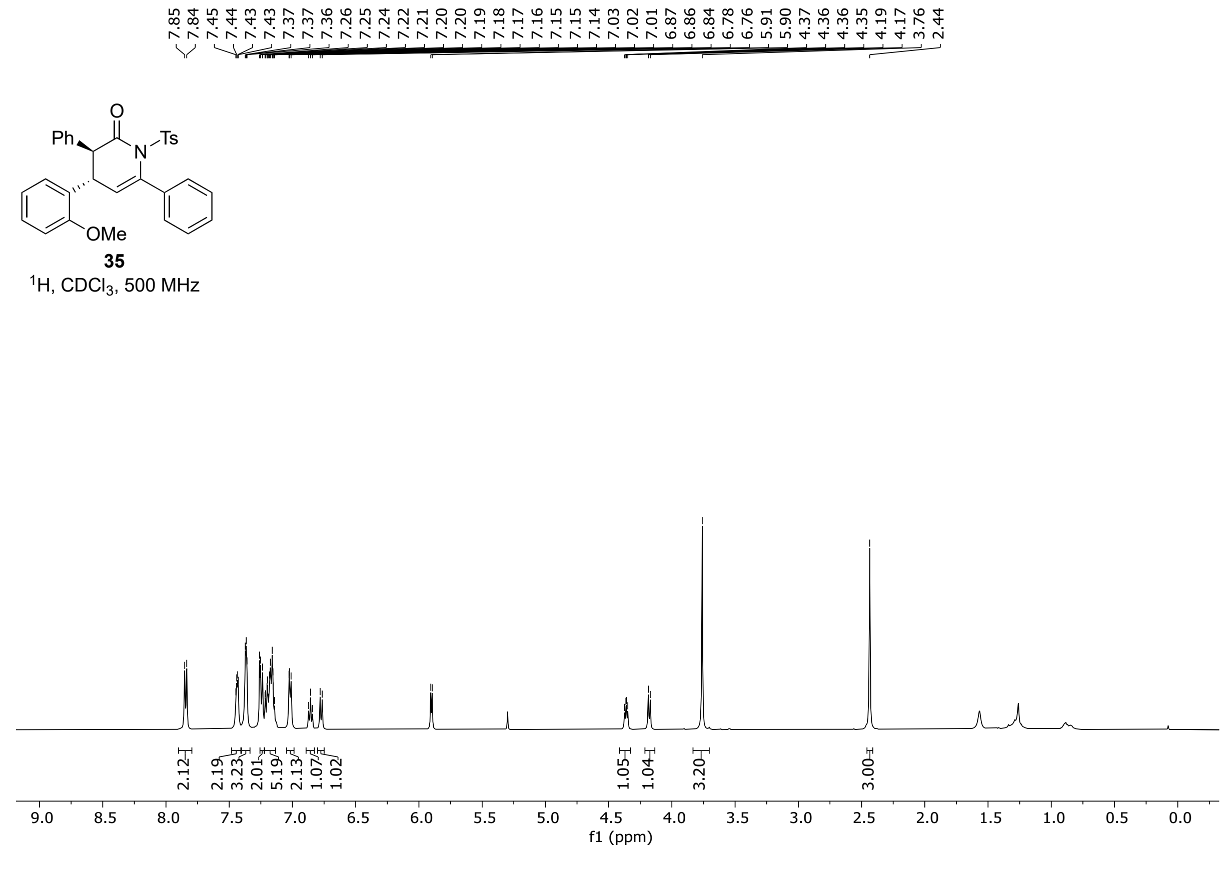

35

^{1}H, $CDCl_3$, 500 MHz

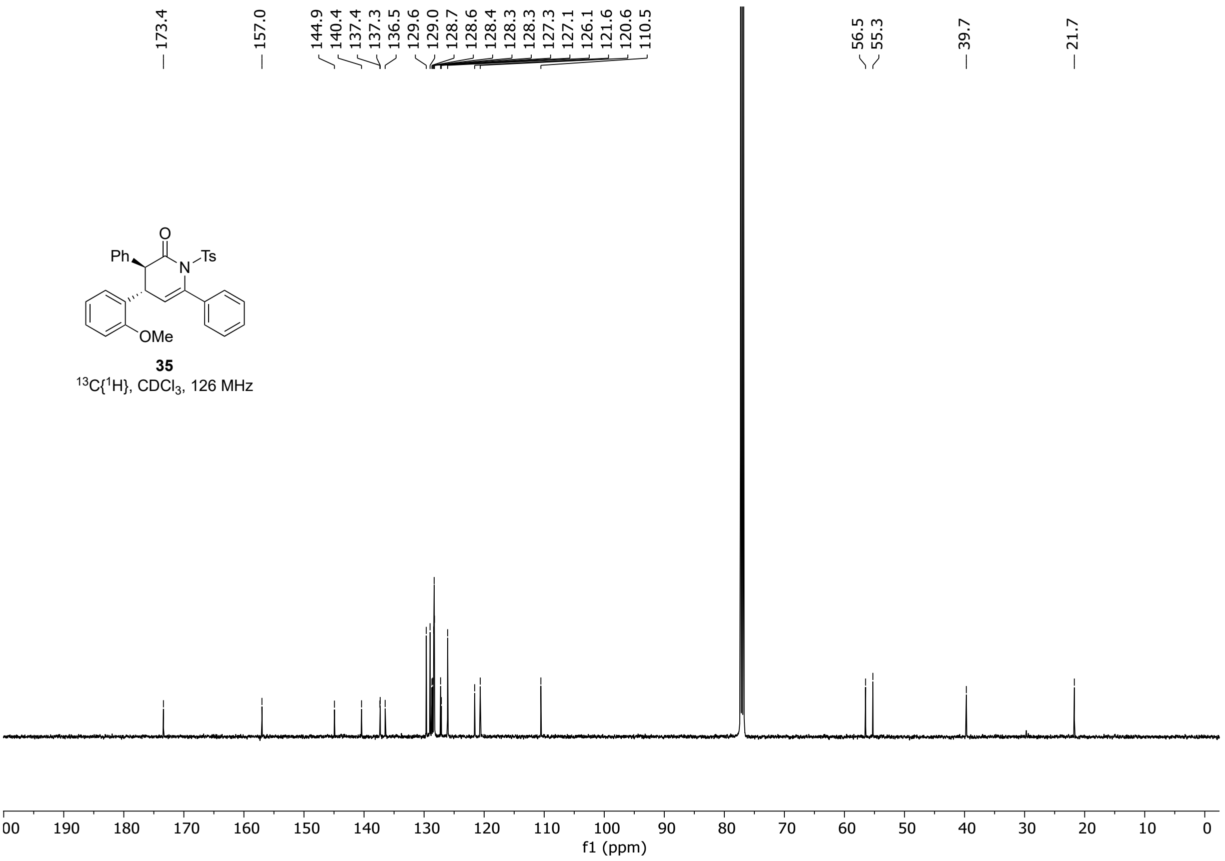
173.4
157.0
144.9
140.4
137.4
137.3
136.5
129.6
129.0
128.7
128.6
128.4
128.3
128.3
127.3
127.1
126.1
121.6
120.6
110.5
56.5
55.3
39.7
21.7
Ph
O
N
Ts
OMe
35
$^{13}C\{^1H\}$, $CDCl_3$, 126 MHz
f1 (ppm)

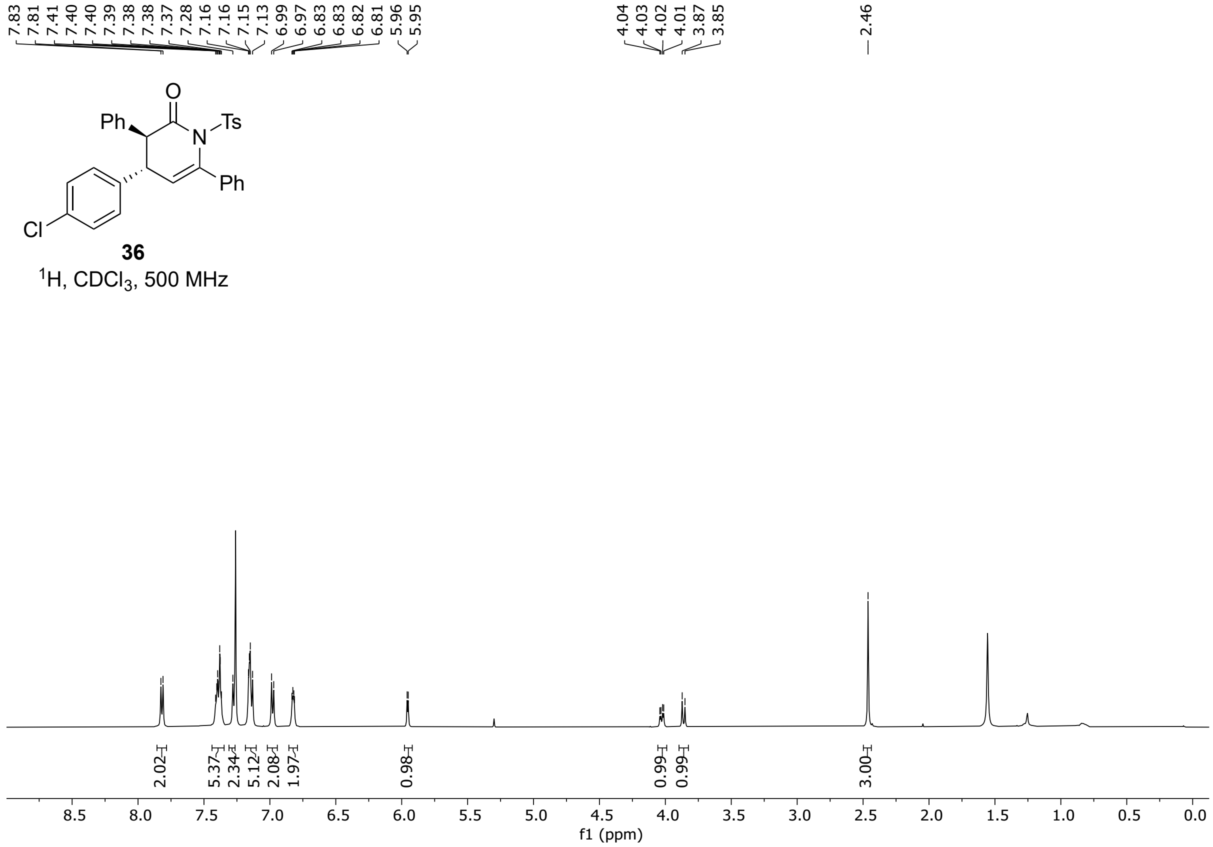
7.83 7.81 7.41 7.40 7.40 7.39 7.38 7.38 7.37 7.28 7.16 7.16 7.15 7.13 6.99 6.97 6.83 6.83 6.82 6.81 5.96 5.95
4.04 4.03 4.02 4.01 3.87 3.85
2.46
O
Ph
N
Ts
Ph
Cl
36
^{1}H, CDCl$_3$, 500 MHz
2.02 5.37 2.34 5.12 2.08 1.97 0.98 0.99 0.99 3.00
f1 (ppm)

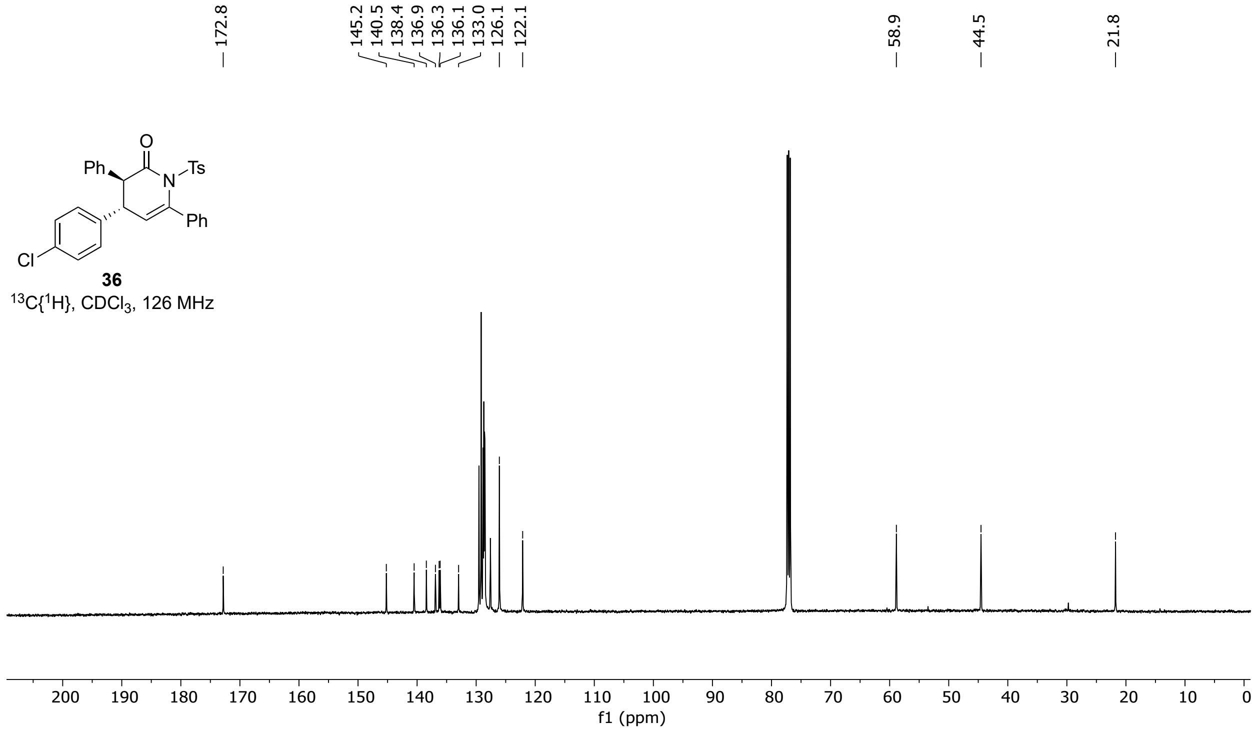
172.8
145.2 140.5 138.4 136.9 136.3 136.1 133.0 126.1 122.1
58.9
44.5
21.8
O
Ph
N
Ts
Ph
Cl
36
^{13}C{^{1}H}, CDCl$_3$, 126 MHz
f1 (ppm)

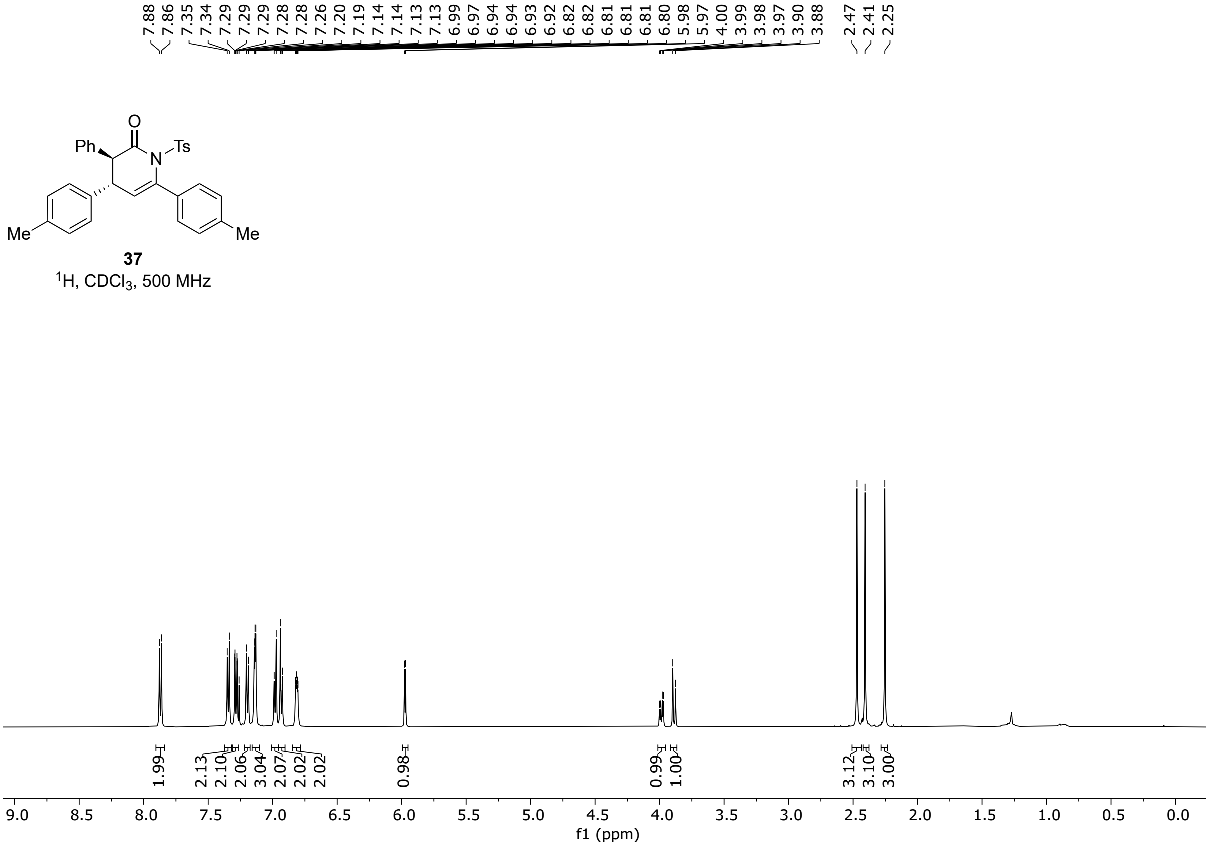
7.88 7.86 7.35 7.34 7.29 7.29 7.29 7.28 7.28 7.26 7.20 7.19 7.14 7.14 7.13 7.13 6.99 6.97 6.94 6.94 6.93 6.92 6.82 6.82 6.81 6.81 6.81 6.80 5.98 5.97 4.00 3.99 3.98 3.97 3.90 3.88 2.47 2.41 2.25
Ph
O
N
Ts
Me
Me
37
1H, CDCl3, 500 MHz
1.99 2.13 2.10 2.06 3.04 2.07 2.02 2.02 0.98 0.99 1.00 3.12 3.10 3.00
f1 (ppm)

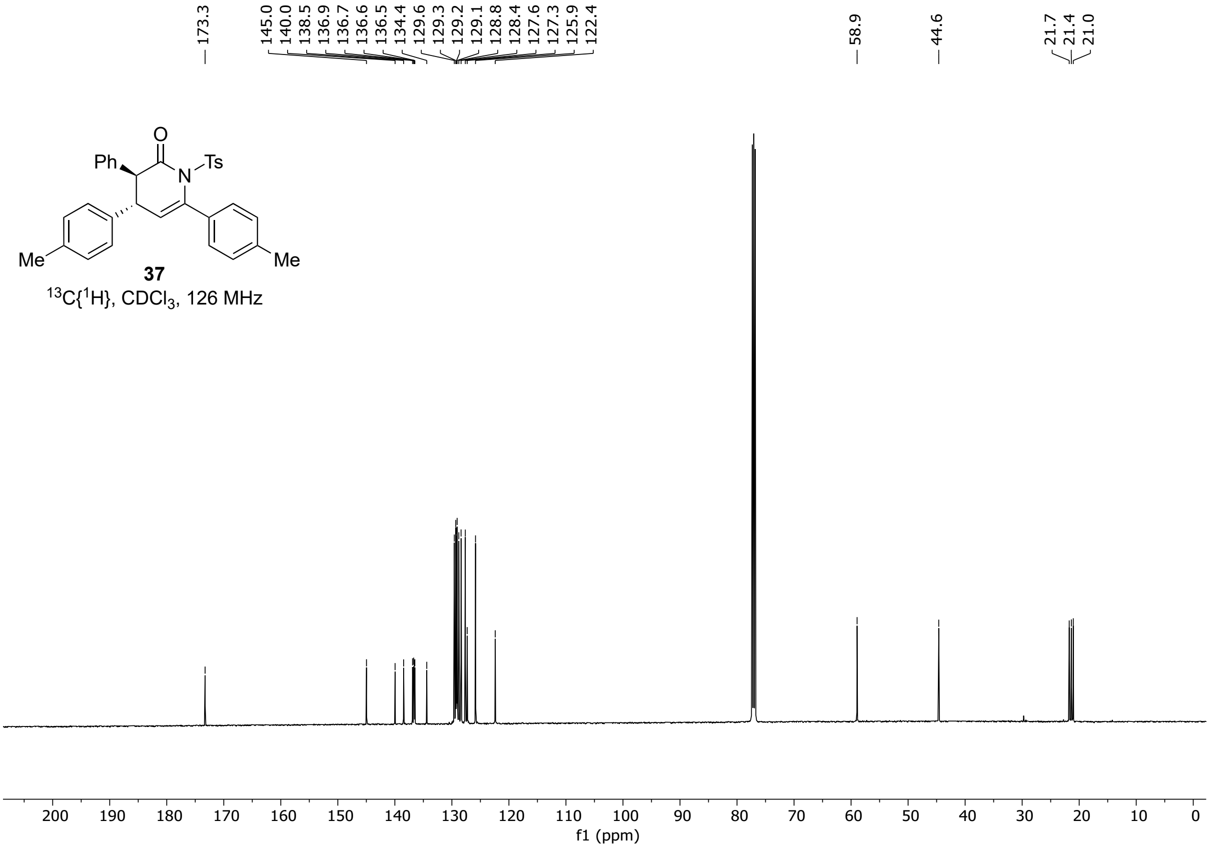
173.3
145.0 140.0 138.5 136.9 136.7 136.6 136.5 134.4 129.6 129.3 129.2 129.1 128.8 128.4 127.6 127.3 125.9 122.4
58.9
44.6
21.7 21.4 21.0
Ph
O
N
Ts
Me
Me
37
13C{1H}, CDCl3, 126 MHz
f1 (ppm)

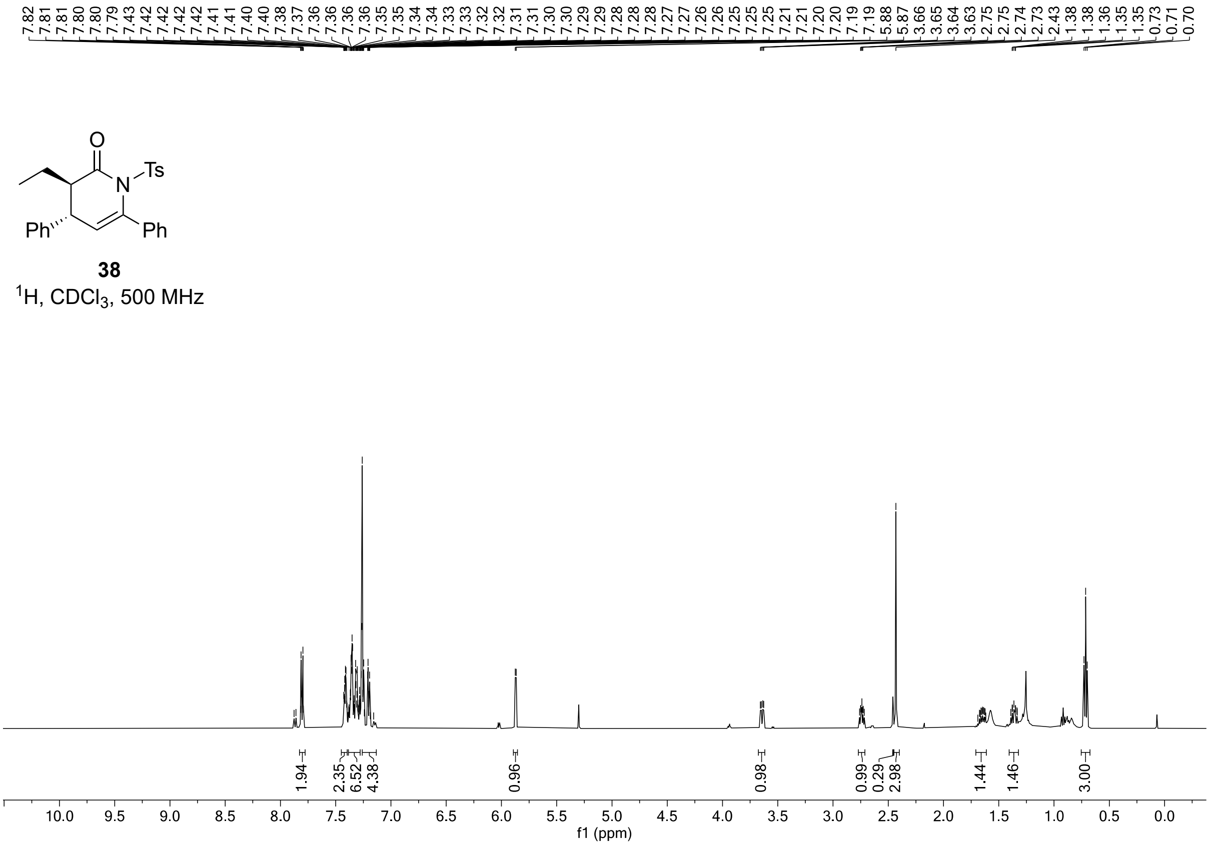

38
^{1}H, CDCl$_3$, 500 MHz
f1 (ppm)

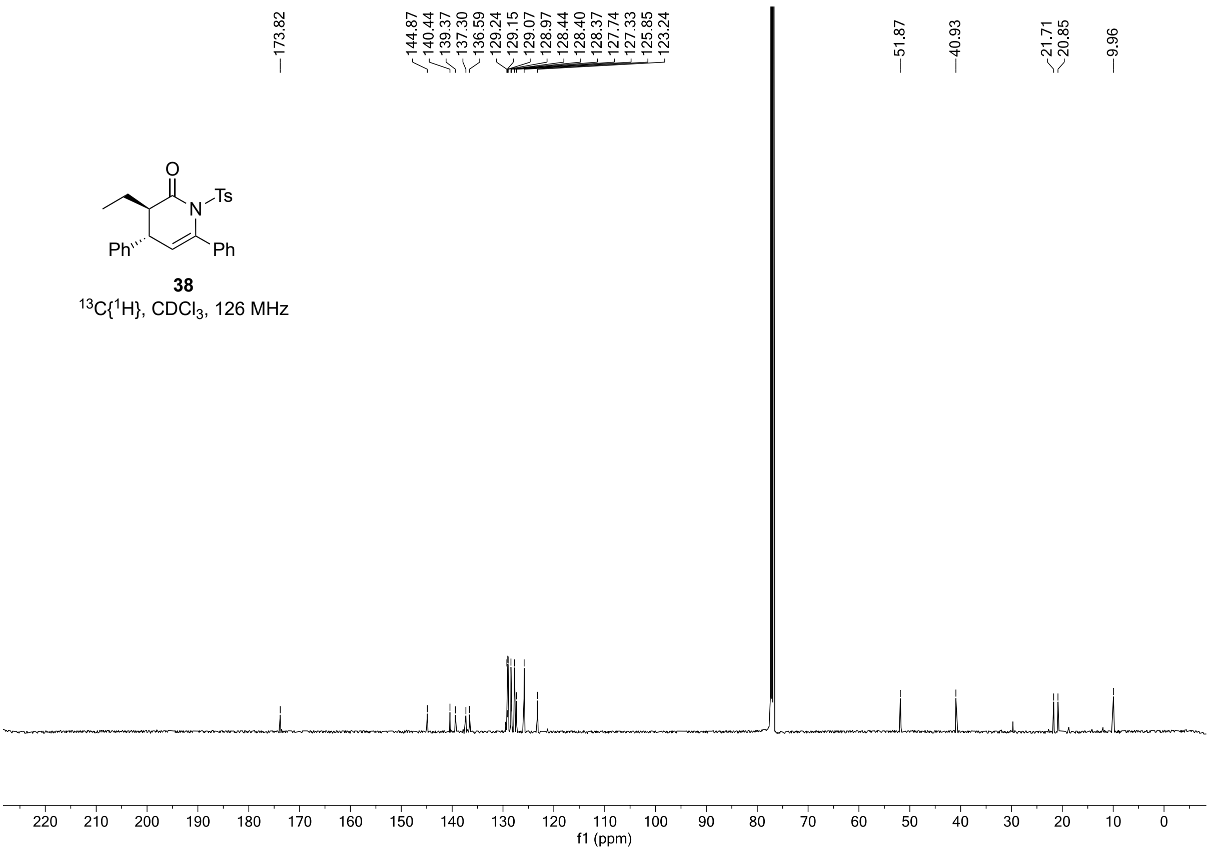

38
^{13}C{^{1}H}, CDCl$_3$, 126 MHz
f1 (ppm)

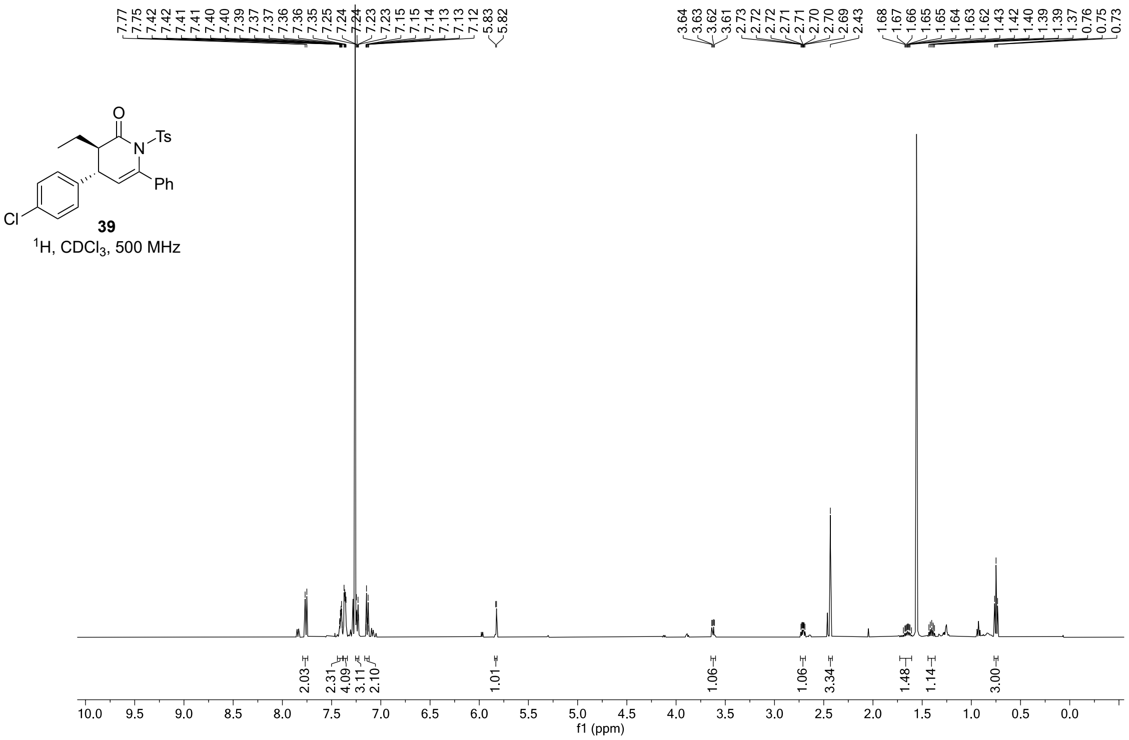

39

^{1}H, $CDCl_3$, 500 MHz

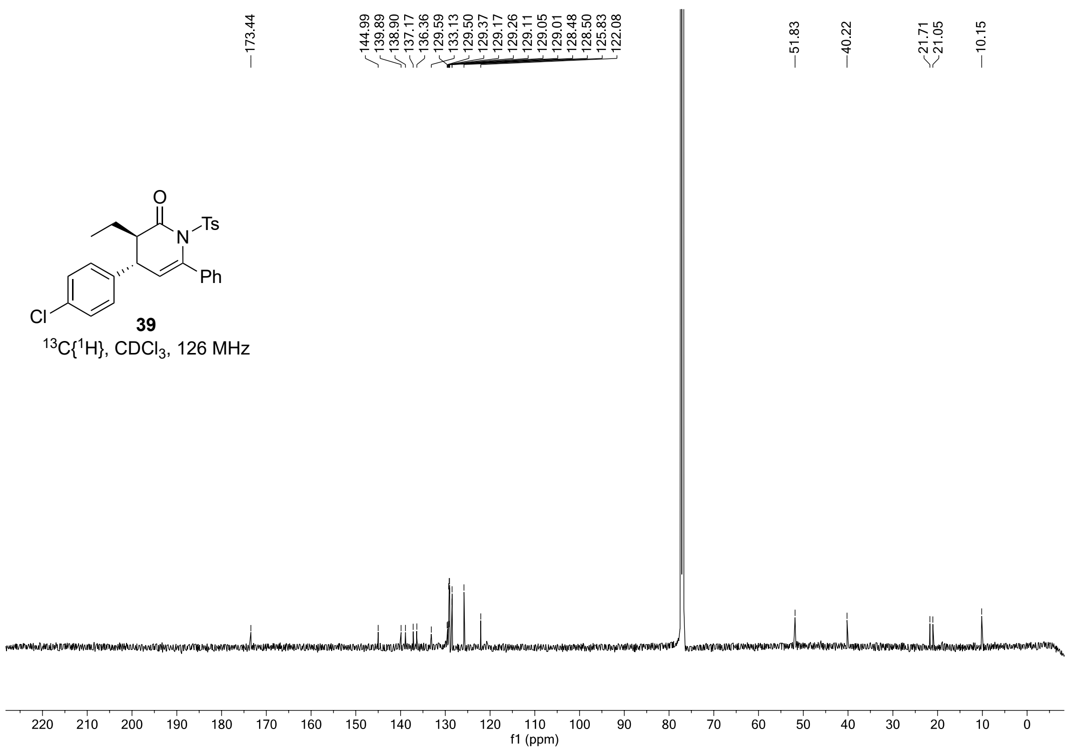

39

$^{13}C\{^{1}H\}$, $CDCl_3$, 126 MHz

1. HPLC Spectra

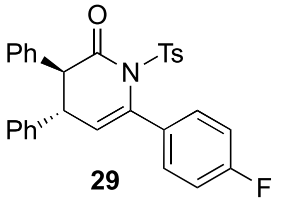

29

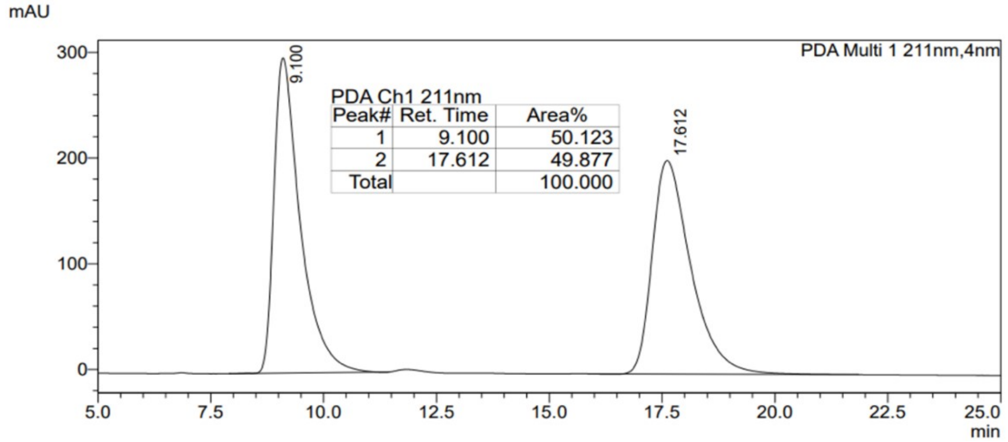

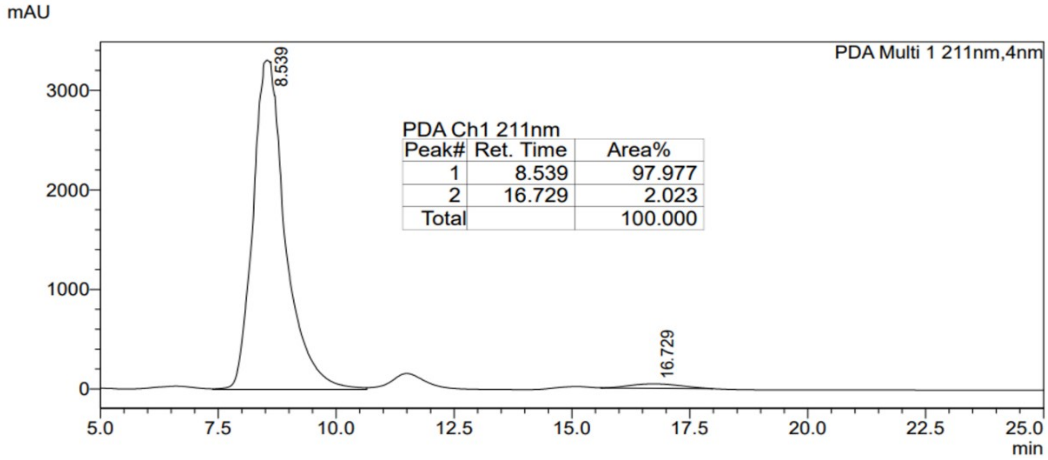

PDA Ch1 211nm

Peak#	Ret. Time	Area%
1	8.539	97.977
2	16.729	2.023
Total		100.000

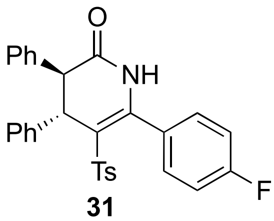

31

<Chromatogram>

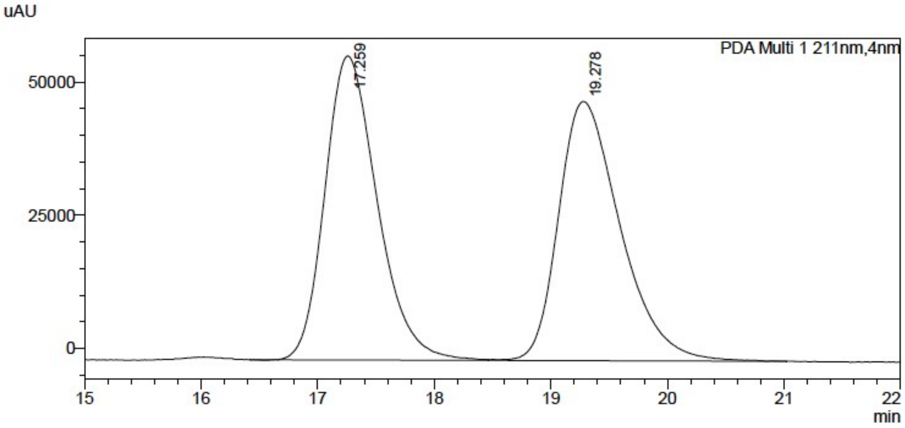

PDA Ch1 211nm

Peak#	Ret. Time	Area%
1	17.259	49.865
2	19.278	50.135
Total		100.000

<Chromatogram>

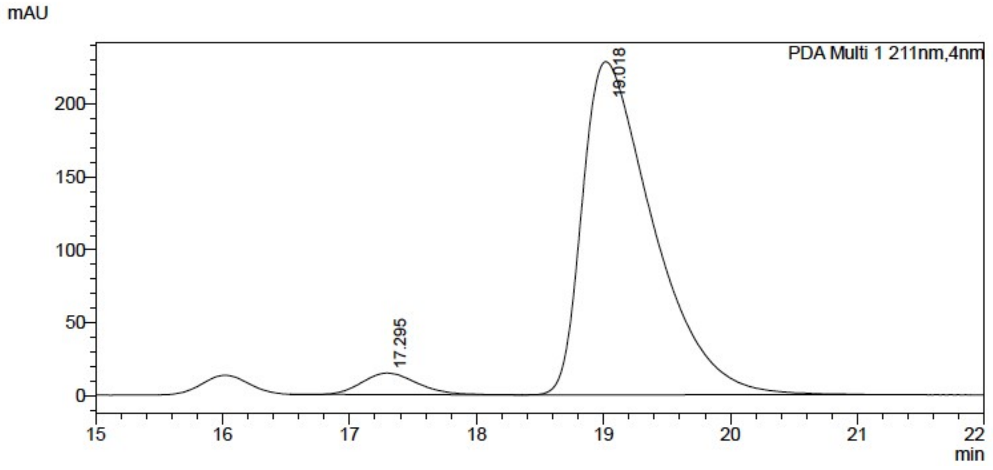

PDA Ch1 211nm

Peak#	Ret. Time	Area%
1	17.295	4.805
2	19.018	95.195
Total		100.000

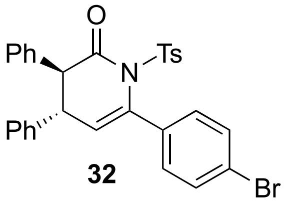

32

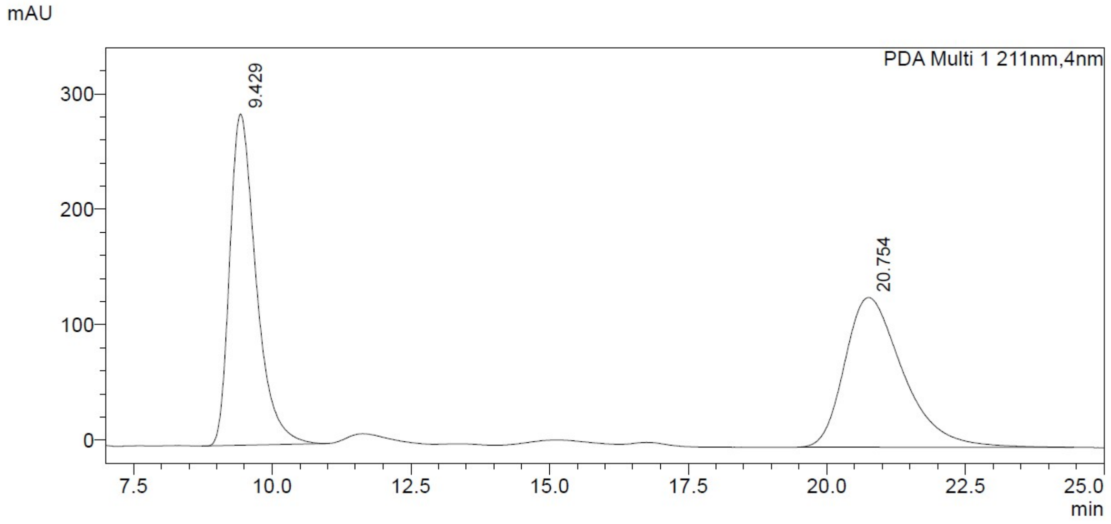

PDA Ch1 211nm

Peak#	Ret. Time	Area%
1	9.429	50.119
2	20.754	49.881
Total		100.000

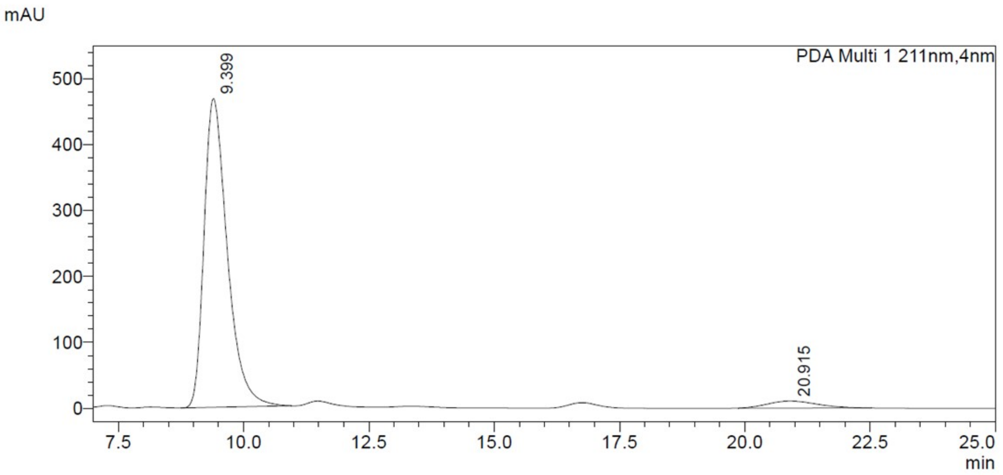

PDA Ch1 211nm

Peak#	Ret. Time	Area%
1	9.399	95.375
2	20.915	4.625
Total		100.000

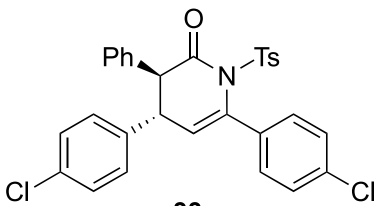

33

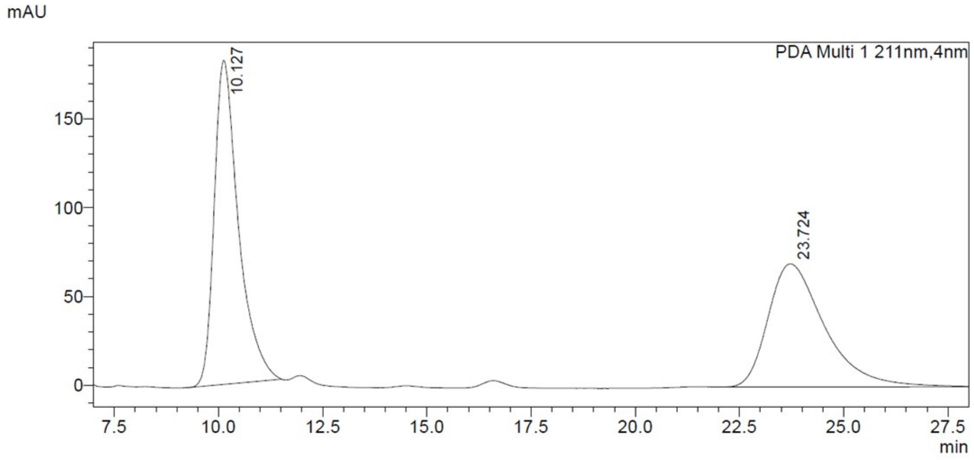

PDA Ch1 211nm

Peak#	Ret. Time	Area%
1	10.127	52.966
2	23.724	47.034
Total		100.000

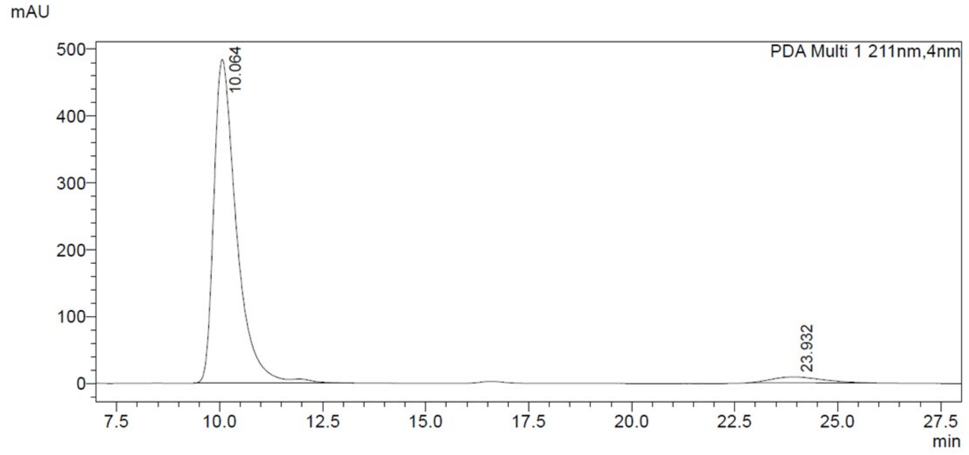

PDA Ch1 211nm

Peak#	Ret. Time	Area%
1	10.064	96.157
2	23.932	3.843
Total		100.000

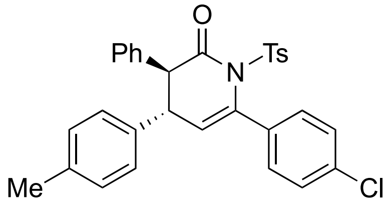

34

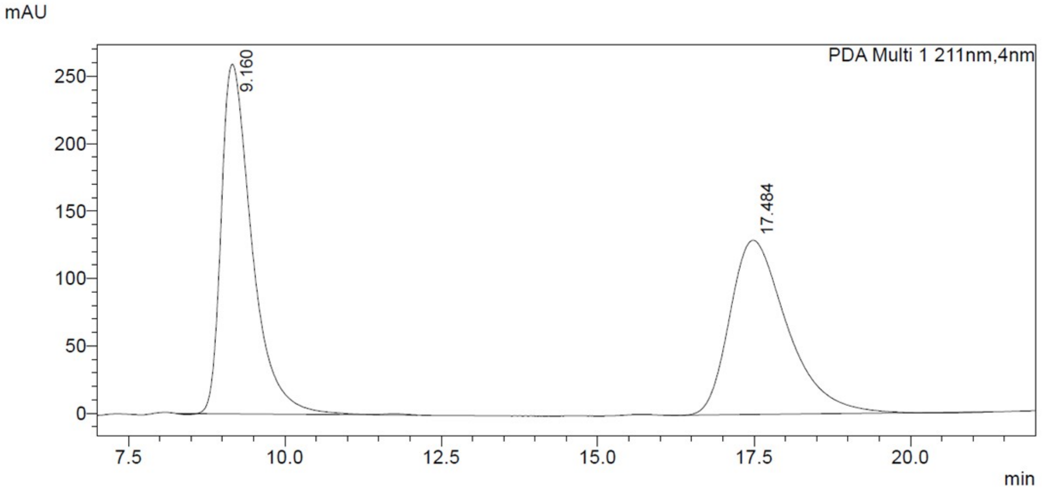

PDA Ch1 211nm

Peak#	Ret. Time	Area%
1	9.160	51.771
2	17.484	48.229
Total		100.000

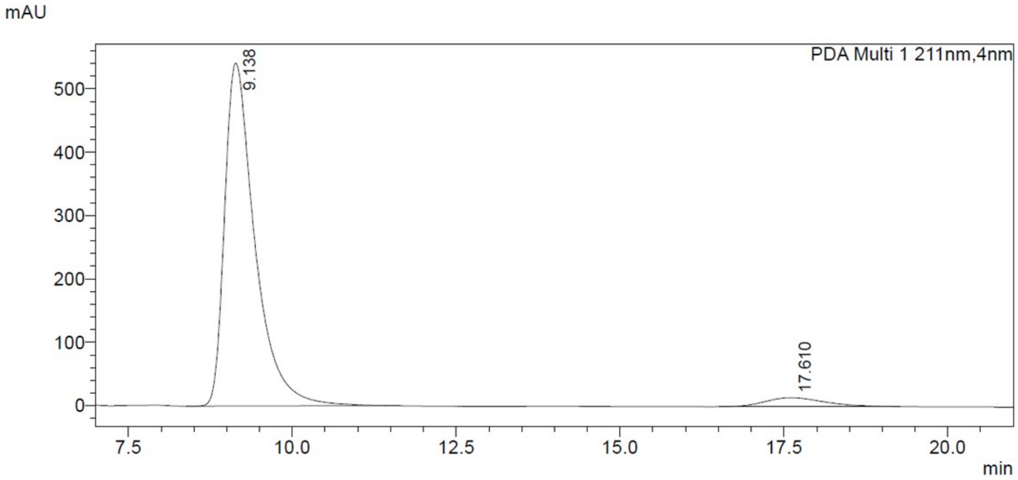

PDA Ch1 211nm

Peak#	Ret. Time	Area%
1	9.138	95.078
2	17.610	4.922
Total		100.000

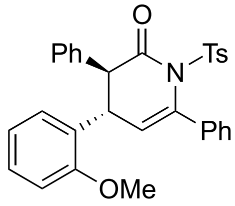

35

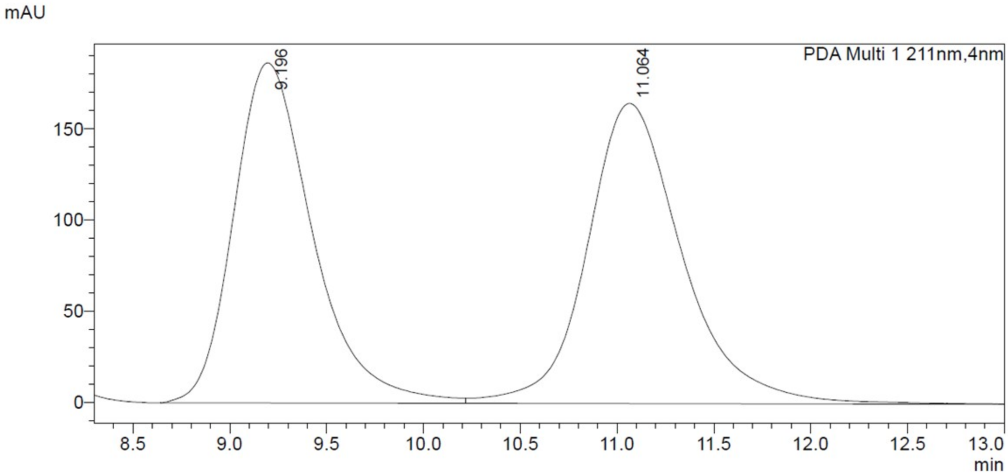

PDA Ch1 211nm

Peak#	Ret. Time	Area%
1	9.196	48.346
2	11.064	51.654
Total		100.000

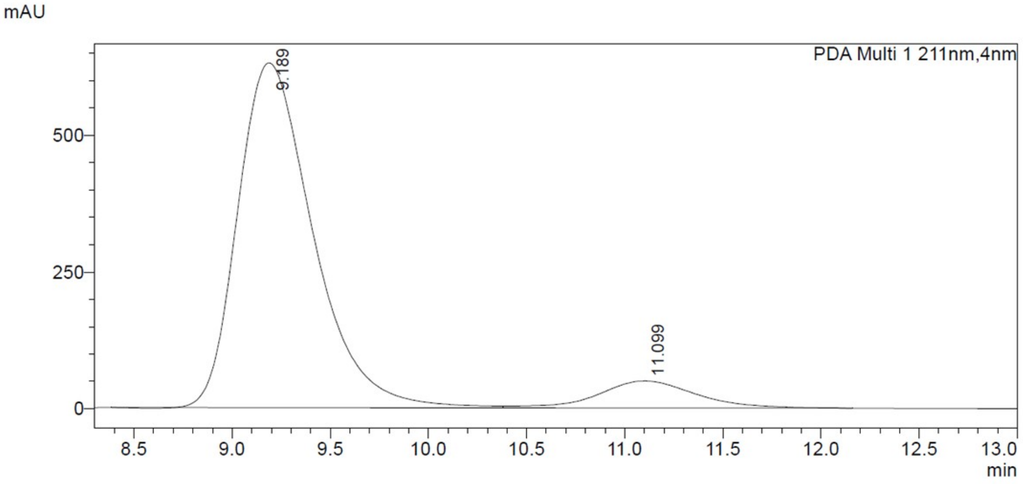

PDA Ch1 211nm

Peak#	Ret. Time	Area%
1	9.189	90.757
2	11.099	9.243
Total		100.000

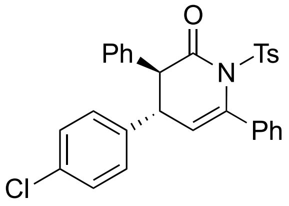

36

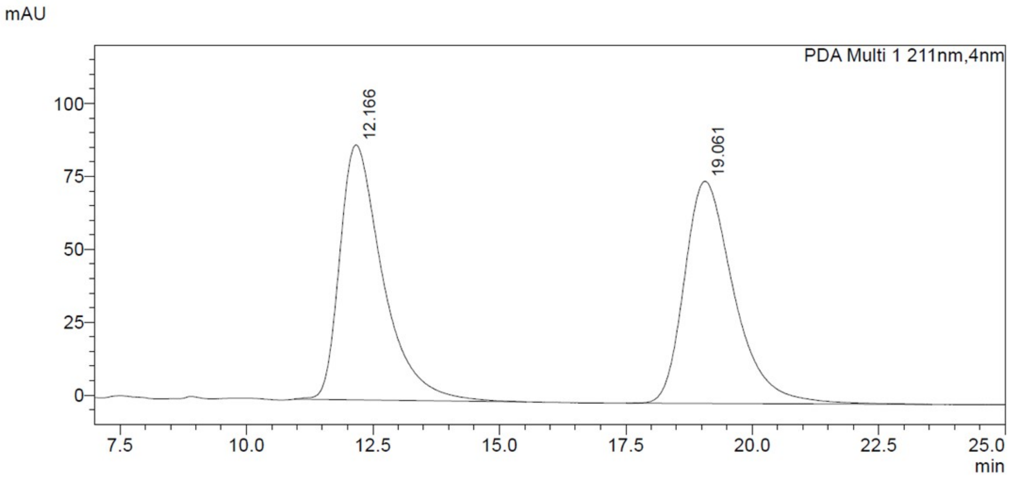

PDA Ch1 211nm

Peak#	Ret. Time	Area%
1	12.166	49.798
2	19.061	50.202
Total		100.000

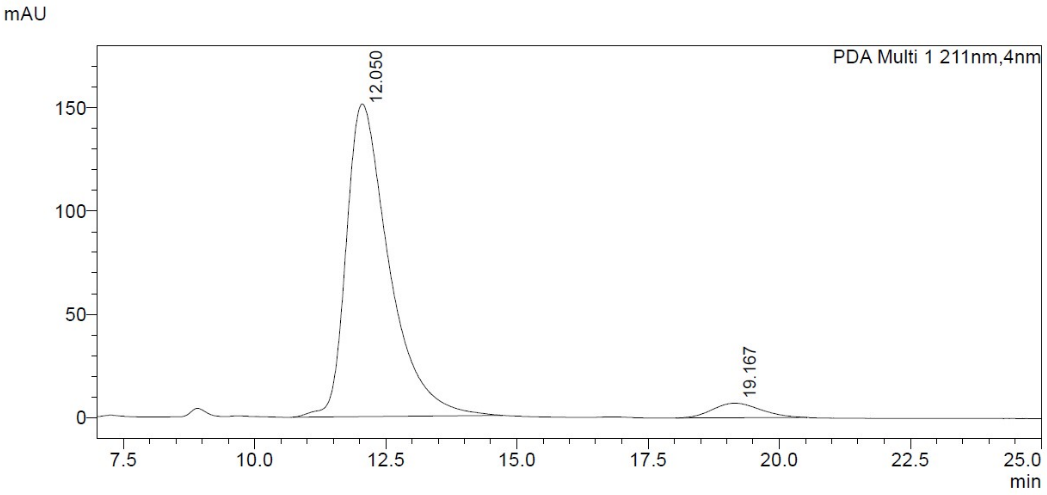

Peak#	Ret. Time	Area%
1	12.050	95.055
2	19.167	4.945
Total		100.000

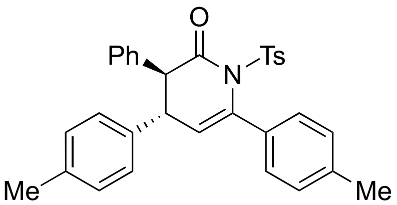

37

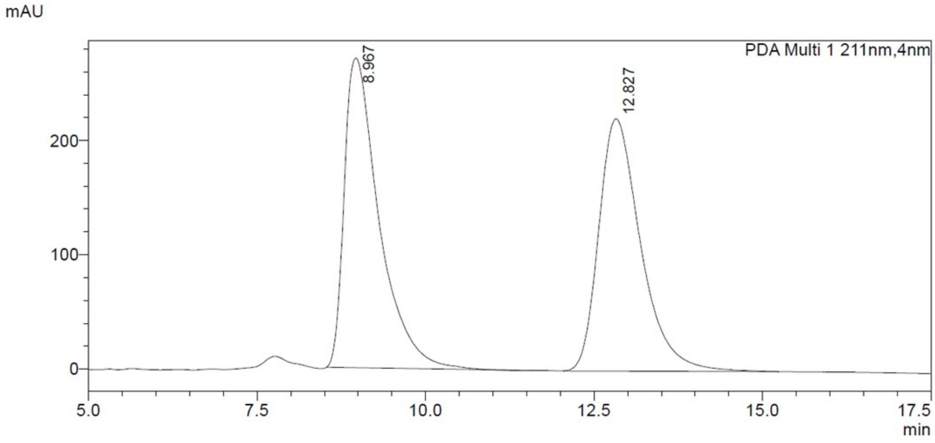

PDA Ch1 211nm

Peak#	Ret. Time	Area%
1	8.967	50.445
2	12.827	49.555
Total		100.000

<Chromatogram>

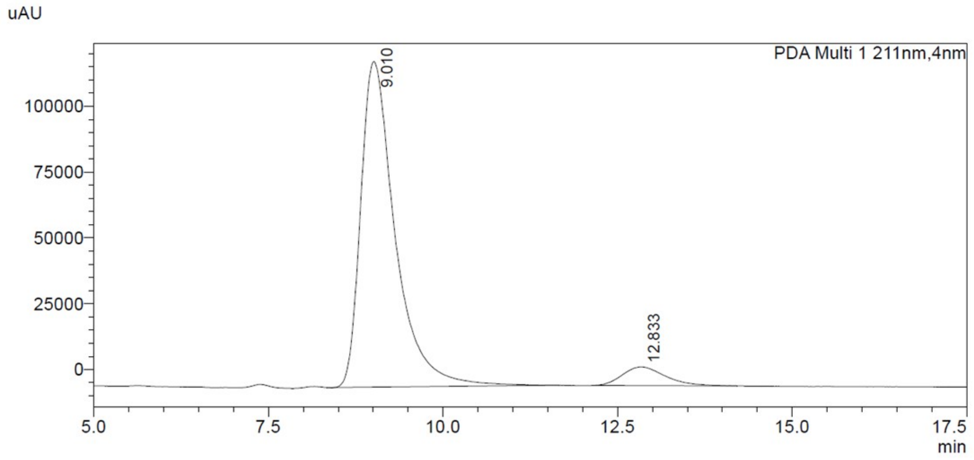

PDA Ch1 211nm

Peak#	Ret. Time	Area%
1	9.010	93.371
2	12.833	6.629
Total		100.000

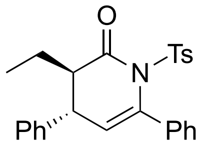

38

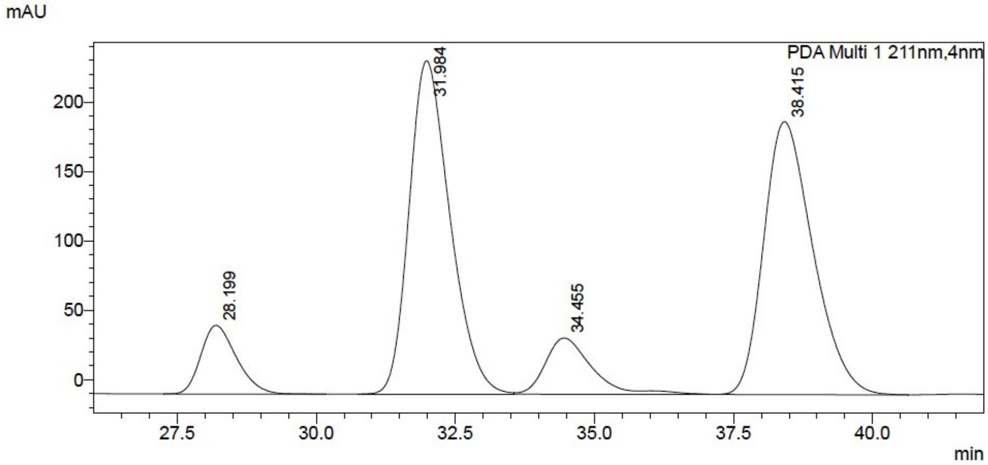

PDA Ch1 211nm

Peak#	Ret. Time	Area%
1	28.199	7.613
2	31.984	42.453
3	34.455	8.190
4	38.415	41.743
Total		100.000

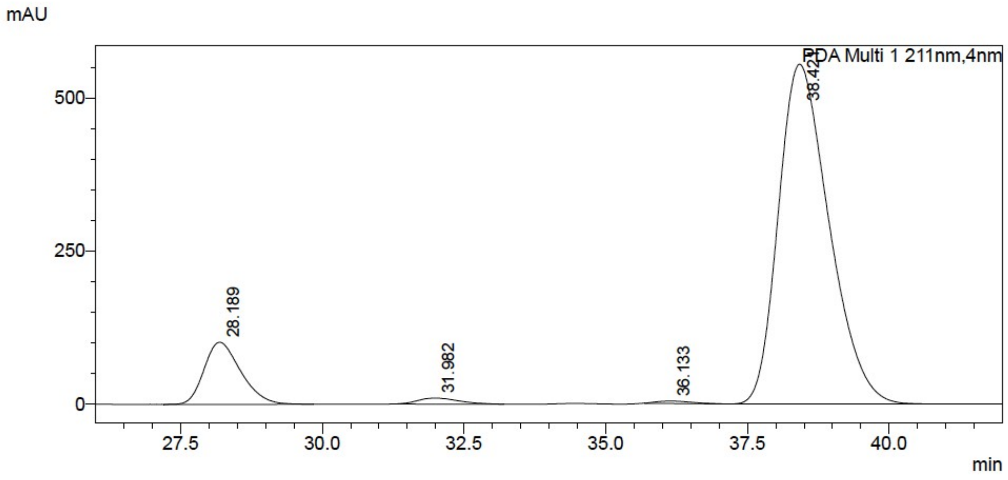

PDA Ch1 211nm

Peak#	Ret. Time	Area%
1	28.189	11.507
2	31.982	1.244
3	36.133	0.437
4	38.421	86.812
Total		100.000

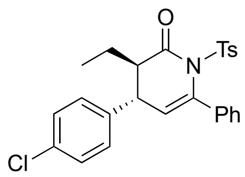

39

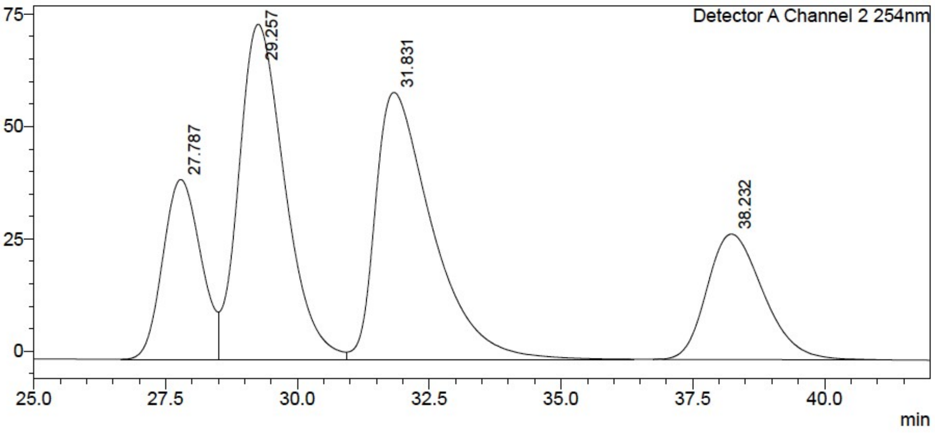

Detector A Channel 2 254nm

Peak#	Ret. Time	Area%
1	27.787	15.391
2	29.257	34.595
3	31.831	34.117
4	38.232	15.896
Total		100.000

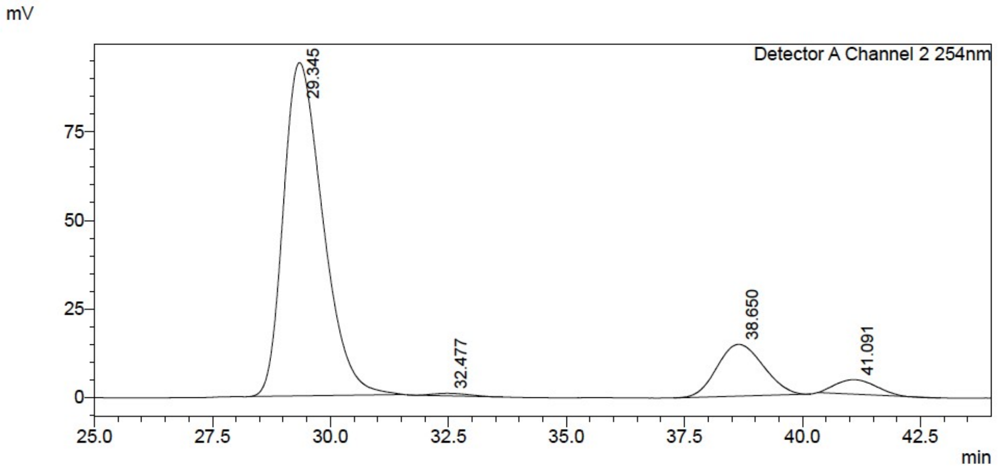

Detector A Channel 2 254nm

Peak#	Ret. Time	Area%
1	29.345	81.093
2	32.477	0.554
3	38.650	14.727
4	41.091	3.625
Total		100.000
